# The effectiveness of interventions to reduce cardio-metabolic risk factors among regular street food consumers in Dar es Salaam, Tanzania: The pre-post findings from a cluster randomized trial (Registered by Pan African clinical trial registry with trial # PACTR202208642850935)

**DOI:** 10.1371/journal.pone.0289289

**Published:** 2023-11-15

**Authors:** Gibson B. Kagaruki, Michael J. Mahande, Mary T. Mayige, Katharina S. Kreppel, Esther S. Ngadaya, Daniel Haydon, Godfather D. Kimaro, Sayoki G. Mfinanga, Bassirou Bonfoh

**Affiliations:** 1 Research Programs, National Institute for Medical Research, Tukuyu Medical Research Centre, Mbeya, Tanzania; 2 Department of Epidemiology & Biostatistics, Institute of Public Health, Kilimanjaro Christian Medical University College, Moshi, Tanzania; 3 Research Programs, National Institute for Medical Research, Headquarters, Dar es Salaam, Tanzania; 4 Department of Public Health, Institute of Tropical Medicine, Antwerp, Belgium; 5 Research Programs, National Institute for Medical Research, Muhimbili Centre, Dar es Salaam, Tanzania; 6 Schoool of Biodiversity One Health & Veterinary Medicine, College of Medical, Veterinary and Life Sciences, University of Glasgow, Glasgow, United Kingdom; 7 Centre Suisse de Recherches Scientifiques en Côte d’Ivoire, Abidjan, Côte d’Ivoire; University of Montenegro-Faculty of Medicine, MONTENEGRO

## Abstract

**Introduction:**

The healthy plate model (HPM) is a practical guide to modulate the portion of staple food in main meals, subsequently affecting the risks associated with Non-communicable Diseases include type2 diabetes mellitus (T2DM).

**Objective:**

This study investigated the effectiveness of health information and the healthy plate model on cardio-metabolic risk factors, knowledge and attitude towards T2DM prevention measures.

**Methods:**

A pre-post analysis, as part of a cluster randomized trial with street food vendors and their customers, was implemented in three randomly selected districts in Dar es Salaam, Tanzania. Two vendor-customer clusters each with 15 and more vendors from each district were randomly assigned to receive either T2DM health information only (Intervention package1 [IP1]) or IP1 plus a subsidized meal with vegetables and fruits, following the principles of the HPM (Intervention package2 [IP2]). Within the clusters the participants were informed on the importance of the intervention they received. An intervention period lasted for three months from 1^st^ April to 31^st^ June 2019. We applied Generalized Linear Mixed Models and Bayesian Modelling (for sensitivity analysis) to assess the effectiveness of the interventions.

**Results:**

Overall, 336 (IP2 = 175 and IP1 = 161) out of 560 (280/arm) previous study participants participated in evaluation. Diastolic BP was lower among IP2 participants in the evaluation than baseline AβC = -4.1mmHg (95%CI:-5.42 to -2.76). After adjusting for the interaction between IP2 and age of the consumers, the BMI was significantly lower among IP2 in the evaluation than baseline AβC = -0.7kg/m^2^ (95%CI: -1.17 to -0.23). With interaction between IP2 and income, BMI was higher in the IP2 in the evaluation than baseline AβC = 0.73kg/m^2^ (95%CI: 0.08 to 1.38). Systolic and diastolic BP were significantly lower among IP1 in the evaluation than baseline AβC = -3.5mmHg (95%CI:-5.78 to -1.24) and AβC = -5.9mmHg (95%CI:-7.34 to -4.44) respectively. Both the knowledge scores and positive attitudes towards T2DM prevention measures were higher in the evaluation than baseline in both interventions arms.

**Conclusion:**

The positive effects on cardio-metabolic risk factors, knowledge and attitude were observed in both intervention arms. Due to interactions between IP2, age and income; designing interventions relating to food and cardio-metabolic risk factors, should consider combining socio-economic factors.

## Introduction

In urban areas, a rising number of people consume meals outside their homes, most often unhealthy foods, i.e., food high in fat, sugar, and salt, hence increasing their risks for developing non-communicable diseases (NCDs) including type 2 diabetes [1–5]. This change in landscape has resulted in a transition of disease epidemiology, as developing countries are facing a growing double burden of infectious diseases and NCDs [6, 7]. Presently, in Tanzania, NCDs are responsible for 33% of all deaths [8]. The cardio-metabolic risk factors, overweight and obesity, raised blood glucose, raised blood pressure (BP), raised triglycerides, and low high-density lipoprotein (HDL), are intermediate predictors of type 2 diabetes [9]. Intermediate risk factors, to a large extent, can be influenced by behavioural risk factors including unhealthy eating practices, physical inactivity, smoking and harmful alcohol consumption [10–14]. Findings from the baseline data collected among regular street food consumers in Dar es Salaam in a previous study linked to the current analysis, revealed high levels of cardio-metabolic risk factors including raised BP (42%), overweight/obesity (62%); raised triglycerides (13%) and raised blood glucose (6.6%) [15]. Healthy plate model (HPM) is a practical guide to modulate the portion of staple food in main meals [16]. Meals designed after the HPM have a protective effect from NCDs including type2 diabetes and cardiovascular diseases [16]. However, getting a healthy plate from street food vendors remains a challenge. Evidence shows that street food meals are served with only small amounts of vegetables and fruits or none at all. Servings of street food are also associated with larger amounts of carbohydrate and protein, thus exposing the consumers to excess calorie intake [17, 18] and increasing cardio-metabolic risks [19–22]. Despite an increase in cardio-metabolic risks factors, in Tanzania, effectiveness of available interventions on cardio-metabolic risks, knowledge, and attitude of the populations at risk of NCDs, including regular consumers of street food, have not been fully explored. These potential interventions include the HPM, which was validated by nutrition experts from the Harvard School of Public Health [23], and a type 2 Diabetes Mellitus (T2DM) health information package currently promoted by the Ministry of Health of Tanzania [24].

The current study involved implementation of two interventions namely: i) intervention package 1 (IP1) consisting of T2DM health information only and ii) Intervention Package 2 (IP2) consisting of T2DM health information and serving of a meal subsidized with vegetables and fruits following the principles of the HPM. Later, the study evaluated if IP1 and or IP2 are effective in reducing cardio-metabolic risk factors, increasing knowledge on T2DM and affecting attitudes towards T2DM prevention measures, as compared to a baseline (pre-post analysis).

## Material and methods

### Study design and setting

This pre-post analysis as part of a cluster randomized trial study was implemented from April 2018 to September 2019 in three out of five randomly selected districts in Dar es Salaam city. The study setting has been previously described in detail in our baseline study [15]. Dar es Salaam is one of the fastest growing urban centres in Africa [25, 26] and with a large population dependent on street food [15, 17, 27], it presented an ideal study site. Another selection criteria was the presence of the headquarters of all the country’s food regulatory authorities, including the Tanzania Food and Nutrition Centres (TFNC), Tanzania Bureau Standards (TBS) and Tanzania Medicines & Medical Devices Authority (TMDA) during the time of the study. In each district, two street markets were chosen for the study. These included: Kawe and Tandale in the Kinondoni district, Mbezi and Manzese in the Ubungo district and Ilala Boma and Kisutu in the Ilala district.

### Study population and sample size calculation

A street food consumer is defined as a person who buys, and eats food or drink prepared in the street or at a supplier’s home, which is then sold in the street/public areas [28, 29]. Using its own operationalized definition, the study defines a regular street food consumer as a person who consumes at least three street vended lunches per week. The sample size for regular street food consumers was calculated by considering the following statistical parameters: level of significance (Z_1-α_) of 95%CI, power of the test (Z_1-β_) of 80, population standard deviation (δ) and population variance (δ^2^). The baseline indicator for the mean waist hips ratio of μ_0_ = 0.82 and a target of reducing the mean waist ratio by 5% after the intervention i.e., from 0.82 to μ_a_ = 0.78. A design effect (DE) of 1.5 to address variance between sites was applied and a non-response rate of 20% was estimated. With the above statistical parameters, and the applied sample size (n) calculation formula [30], the desired sample size was 280 participants. This sample size was doubled to include both arms (IP1 and IP2).

### Eligibility criteria for market site, street food consumers and vendors

A market site was eligible for the study if it had at least 15 street food vendors. Regular street food consumers: A consumer was eligible for the study if he/she met the following criteria: being aged 25–64 years; consuming at least three lunches per week at the same street food vendor; having no plans to move out of the study area in the next 12 months; and have been consuming street food for not less than a year. Lactating mothers and pregnancy women were not eligible. Eligibility criteria for street food vendors included being aged 18 or above; has been vending food at the current site for at least 12 months; has at least seven customers who have been consuming at least three lunch meals per week for at least 3 months; is ready to implement the components required for an intervention; and will continue vending food at the same market site for at least another 12 months.

### Allocation of study market sites/clusters

Three out of five districts included Kinondoni, Ubungo and Ilala, which were randomly selected from Dar es Salaam city. From each sampled district, the study mapped all markets sites (clusters) then two markets were selected from each district randomly. In each district, one cluster was randomly assigned to receive IP1 and the second cluster to receive IP2 using a simple randomization technique [1] by an independent statistician.

### Recruitment of vendors and consumers

**Fig 1:**
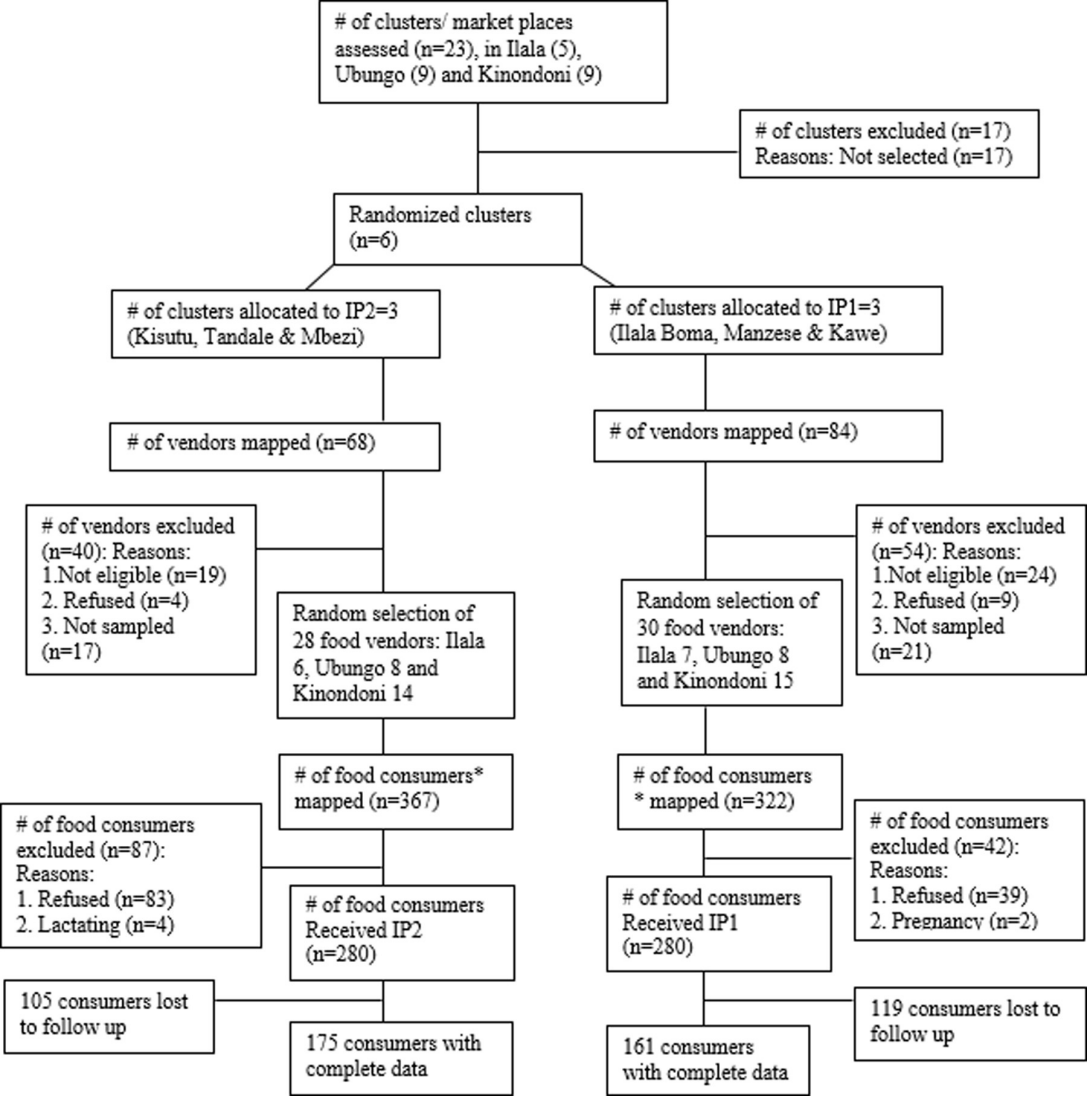
CONSORT chart showing the phases of a parallel cluster randomised trial of two groups include enrolment, intervention allocation, follow-up and data analysis. *Number of food consumers considered were the regular street food consumers i.e. those who consumed three or more lunches per week from the same food vendors; # stands for “number”.

Street food vendors were mapped at each selected market site. From 152 food vendors in 6 selected markets (sites), 58 food vendors were randomly selected; Ilala (13), Ubungo (23) and Kinondoni (22). In each selected market, 90 regular street foods consumers were recruited with a minimum of 7 regular consumers per vendor. Finally, 280 consumers were allocated into IP1 market sites and 280 were allocated to IP2 market sites. Allocation of both the clusters and study participants in the two study arms was at a ratio of 1:1 (**Fig 1**).

The recruitment of study participants including seeking consent took place from November 2018 to March 2019. The process involved awareness creation and sensitization of street food consumers and vendors with information education communication (IEC) such as brochures and banners. In this phase the principle investigator together with the food vendors and their leaders were also screening eligible participants. Eligible vendors were asked to provide the names and contact details of their regular clients. This was possible because regular consumers typically call vendors to have food delivered to their workplace. Vendors were asked to list all of their regular customers to avoid selection bias; vendors were informed in advance that only consented customers will be included in the study. With the customers’ list, the principal researcher approached each of the customers and gave information about the study and sought informed consent. Finally, a list of eligible and consented participants per cluster and vendor was obtained. All eligible and consenting participants were informed about the start and end date of the intervention. Specifically, the intervention period lasted for three months from 1^st^ April to 31^st^ June 2019.

### Intervention packages

#### Intervention package 1 –IP1 (T2DM health information)

This study used the One Health approach [31] for delivering T2DM health information to participants; the interdisciplinary team involved a physiologist from the National Hospital-Muhimbili, an epidemiologist from the National Institute for Medical Research and a nutritionist from the Dar es Salaam Regional Office. The participants were informed on the behavioural, familial, and metabolic risk factors for type2 diabetes and on prevention measures of diabetes. These measures included the recommended level of metabolic equivalent task per week (MET/Week) of physical activities, the recommended limit of alcohol to drink per week for each sex, and the recommended amount of fruit and vegetable servings to be consumed each day. Furthermore, the participants were informed of the risk of smoking and eating excess fats, carbohydrates, and fruit high in calories. The signs, symptoms, and complications of type2 diabetes were also explained to the participants. The channels for delivering T2DM key health information included group meetings, text messages on their mobile phones, and the distribution of brochures and banners.

#### Intervention package 2-IP2 (T2DM health information and healthy plate model)

This package comprised two components, T2DM health information and a subsidized plate model, enabling vendors to sell subsidized meals following the plate model principles. The study adopted a plate model validated by nutrition experts from the Harvard School of Public Health [23]. The pictorial healthy plate model illustrates the recommended portions of the main food groups namely carbohydrate (1/4 of the plate), protein (1/4 of the plate), fruits and vegetables (1/2 of the plate), per person/meal [23]. To ensure optimal adherence to the plate model, the project subsidized costs (0.45USD/consumer/day) of fruits and vegetables in the three market sites receiving IP2. The consumers and vendors from the IP2 arm were informed on how to prepare the recommended food plate, and on its potential role in reducing cardio-metabolic risk factors. It was envisaged that knowledge of the healthy plate model would influence practice and increase uptake of an intervention among consumers. The benefits of serving dishes corresponding to the healthy plate model were explained to the vendors, and these benefits included serving smaller amounts of staple food commonly available in Tanzania [32], these groups include carbohydrate (1/4 of the plate of rice, banana, stiff porridge/ugali, sweet or round potatoes etc.) and balancing other food groups including fruit (sweet bananas, mangoes, oranges, pineapples, water melon, pawpaw etc.), vegetables (spinach, amaranth, pumpkin and cassava leaves etc. (1/2 of the plate) and protein such as fish, white and red meat, beans etc. (1/4 of the plate).

#### Implementation steps

Implementation of this study involved four steps. In the first step, baseline data was collected to serve as control from August to September 2018. This phase involved collection of information of socio-economic and demographic characteristics, NCDs’ behavioural risks, cardio-metabolic risks, familial risks, knowledge on T2DM and attitude towards T2DM prevention measures. Phase 2 ran from November 2018 to March 2019, which involved awareness creation and sensitization of street food consumers and vendors with information education communication (IEC) such as brochures and banners. Awareness creation also involved group meetings, training of street food vendors on how to prepare a balanced diet and serving a “model plate” to the IP2 group. The messages included in the IEC materials were adapted from information and education communication materials developed and validated by TANCDA in collaboration with the Ministry of Health (MoH) of Tanzania TANCDA et al. [Unpublished]”) [24]. The third step lasted for three months from April to June 2019. In this phase, the interventions were implemented, with the health information provided to street food consumers on T2DM for one group, and health information on T2DM and the healthy plate model served to the other. The last step ran from August to September 2019 and involved the collection of evaluation data among study participants.

#### Study conceptual framework

**Fig 2:**
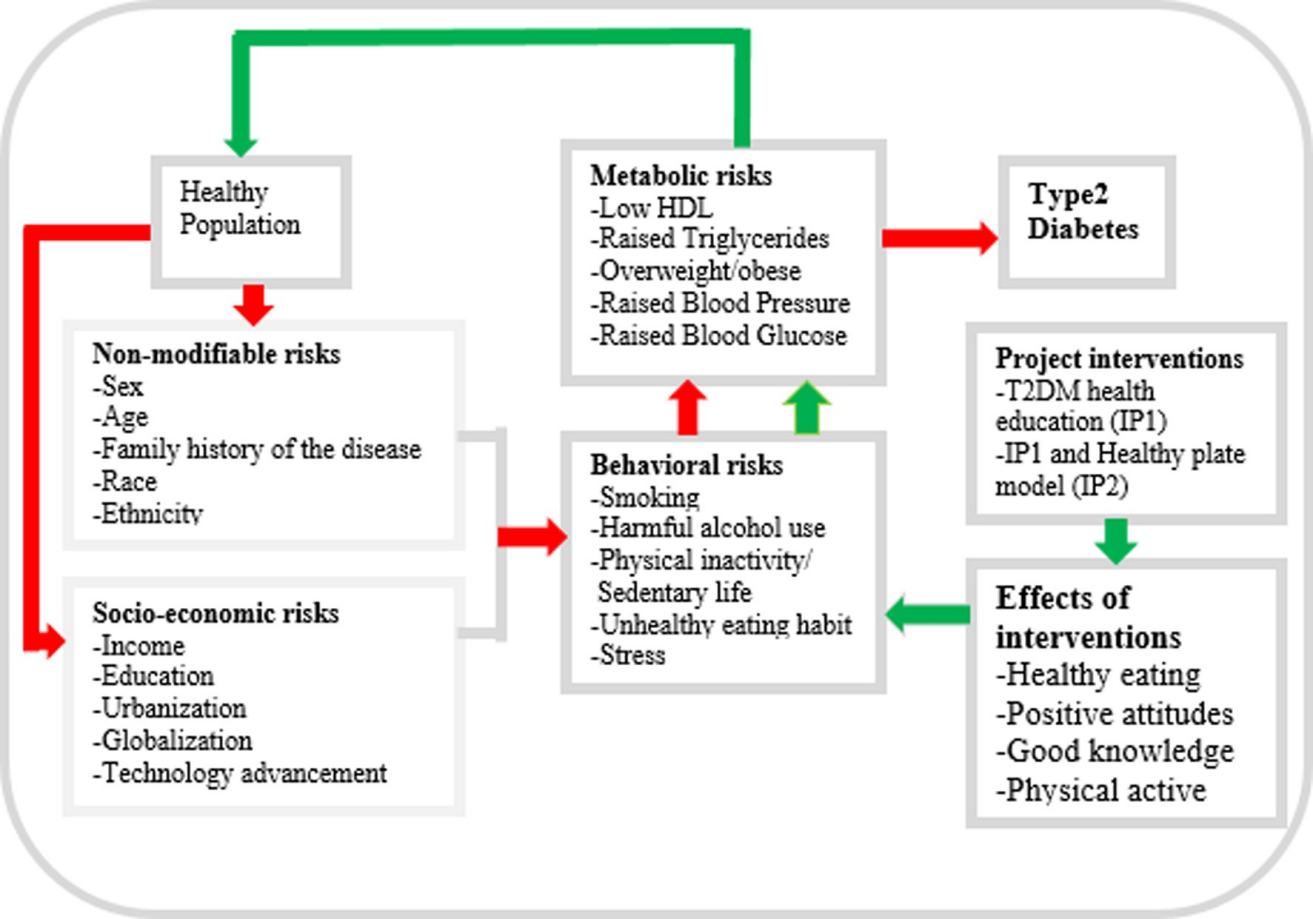
Conceptual framework showing connections between behavioural and metabolic risk factors and pathogenesis of Type2 diabetes and the role of interventions on causality; Modified from Roman-urrestarazu et al. and Budreviciute et al [3, 4].

The study summary concept is described in **Fig 2**. The assumption is that the population in general is healthy. However, after being exposed to socio-economic and non-modifiable risks factors, that population becomes vulnerable to behavioural risks. These include smoking, excessive alcohol consumption, physical inactivity, and unhealthy eating practices, among others. The hypothesis is that if no strategic prevention measures are put in place to reduce these risks, behavioural risks trigger intermediate risks such as low HDL, raised triglycerides, overweight/obesity, raised blood pressure and blood glucose, and as a result the population becomes vulnerable to developing T2DM. The intervention packages (IP1 and IP2) aim to increase positive attitude towards behavioural prevention measures and improve knowledge on diabetes so as to reduce risk behaviours. This will work toward the ultimate goal to reduce intermediate risks factors and in turn aid the population in preventing the development of T2DM.

### Study variables

#### Dependent variables

In this study, the primary outcome was cardio metabolic risk factors (i.e., BMI, blood glucose, triglycerides, and waist circumference, systolic and diastolic BP). Correct knowledge on T2DM and positive attitude scores towards prevention measures of T2DM were the secondary outcomes of interest. All dependent variables were modelled as continuous variables.

#### Independent variables

The independent variables include age, sex, marital status, income, education level and exposer status to intervention (baseline and IP1 or IP2); these were considered as fixed independent variables. The two random effects included were market sites and participant identification numbers.

#### Effectiveness/impact of intervention

The goal was to run pre-post analysis to assess if there was a significant difference in primary and secondary outcomes between IP1 and baseline and IP2 against baseline (pre-post comparative analysis).

#### Data collection and management

The data collection team involved three laboratory technicians, two nurses, two sociologists, one demographer, three medical doctors and a statistician. They had similar levels of experience on the studied topic. However, they participated in a three-day training conducted by principal investigator before the baseline and evaluation phase, which involved briefing them on the study protocol, data collection tools and ethical considerations. In the evaluation phase, the same procedures as for the baseline data collection were adhered to [15]. Information collected included socio-economic and demographic characteristics, cardio-metabolic risk factors: weight and height for body mass index, blood pressure (BP), fasting blood glucose and triglycerides. All tests and measurements were undertaken as per recommended standards and operating procedures [9, 33–40]. Blood samples were taken and stored in a cool box and shipped immediately after collection to St. Laurent Diabetes Centre, Dar-es-Salaam, for analysis on the same day by trained laboratory technicians. Data was collected electronically using the Open Data Kit (ODK) system [41]. Data was cleaned and later synchronized on the main server at the National Institute for Medical Research Muhimbili Centre in real-time. The anonymity of the participants was ensured by assigning a unique identifier to each participant. Further details regarding data collection procedures are published elsewhere [15].

Twenty-four (24) items were adopted from different grey literature and used to assess the knowledge level of each regular street food consumer [37, 42–46]. All items have been used previously in similar studies that were conducted with different populations (HIV positive clients and the general population) [39, 47] in Dar es Salaam. Possible responses for knowledge assessment under each sub domain were listed and if mentioned by the respondent, a “yes” response was recorded and “no” if not mentioned in each respective domain. A weight of “1” was given to each mentioned item and “0” if not mentioned. Participants were also assessed on their knowledge on the daily-recommended quantity of foods from different food groups (protein, fats, carbohydrates, fruits and vegetables, high-fibre food, red meat, glycaemic foods, and salted foods). They were asked to judge the quantity of food to be consumed per person per meal as “large,” “average,” “small” or “not sure.” A correct response was given a score of “1” and a wrong or absent response received “0”. This study also assessed the attitude of study participants towards prevention measures for cardio-metabolic and type2 diabetes. This study also involved reviewing of different published articles [37, 42–46] to generate 12 points to assess the attitude of street food consumers towards available prevention measures for NCDs including T2DM, cardiovascular diseases, and heart disease. The participants who agreed with a positive statement and those who disagreed with a negative statement were considered as having positive attitude and were given a score of “1” while those who reported disagreeing with a positive statement and agree with a negative statement were given a score of “0”. Individual absolute scores for both knowledge and attitude absolute scores were summed and converted into percentages. The items used to assess knowledge and attitude have been summarized in **Appendices I & II of S2 File.**

### Statistical analysis

Cleaning and descriptive data analysis were performed using Stata version 15.0 (StataCorp. 2017. Stata Statistical Software: Release 15. College Station, TX: StataCorp LLC). Comparisons of both explanatory and outcome variables between intervention arms was performed using both Chi-Square tests for categorical variables and T-tests for continuous variables to assess differences between IP1 and IP2 at the baseline phase. Explanatory variables were categorized into random and fixed variables, to overcome dependence problem resulting from repeated measures. The study applied Generalized Linear Mixed Models (GLMM) to accommodate fixed and random variables [48–50]. Assessment was done for the interaction between intervention(s) and age, sex, income, and education. Sensitivity analysis was also conducted to assess if variables which were statistically significant in the GLMM model, were also supported using a Bayesian modelling technique fitted with Markov Chain Monte Carlo (MCMC) but without consider prior information. The results reported in detail in this paper are those generated from the GLMM model while the Bayesian model results have been reported as in the Appendix V in S2 File. All advanced analyses were conducted using R-Software. Both lme4 and MCMCglmm were R packages used while lmer and glmer were the commands used to fit the mixed effects models. These packages and commands assume the random effects to be Gaussian distributed, and the random effects were modelled as independent [49, 50]. Model selection; to select the most parsimonious models, likelihood ratio tests were undertaken [51–53]. Correlation between the baseline measure and end time measures repeated on the same sites and the same subjects were expected, so this study also reported between site and within-subject Interclass Correlation Coefficients (ICCs) [54, 55]. In this study, low and high variability in the outcomes results was observed among sites and subjects, respectively (**Appendix III in S2 File**). To ensure reliable inference, residual analysis was performed and outputs are included as Appendix IV in S2 File, the findings from that analysis showed that there are not substantive deviations from the model assumptions that need concern this study.

## Results

### Characteristics of the participants

Recruitment and seeking consent from study participants started from November 2018 to March 2019. This stage was followed by intervention and follow up phase which lasted for three months from 1st April to 31st June 2019. Of the 560 enrolled participants, 60% (n = 336) participated in the evaluation. The comparison between the IP1 and IP2 arm by socio-demographic at baseline is shown in **Table 1**. There were no significant differences for compared variables between arms. The mean age of both IP1 and IP2 participants was equal (45 years) and more than half of the participants in both arms were aged 41–64 years. More than half of the participants in both arms were male and more than 88% had primary level of education. Above three quarters of participants in both arms were married/cohabiting and above 50%, in both arms their monthly income was above mean values. **Table 2** shows the comparison of both primary (physical and biomedical measurements) and secondary (correct knowledge and positive attitude scores) outcomes between IP2 and IP1, again there was no significant difference between IP1 and IP2.

**Table 1:** Distribution of socio-economic and demographic characteristics of regular street food consumers in Dar es Salaam, by intervention arm (n = 336)-Using Chi-square test.

Characteristics	Interventions	
	T2DM Health information only (IP1), n (%)	T2DM Health information & Plate (IP2), n (%)
Population	161 (47.9)	175 (52.1)
Age (years)		
Mean (SD)	45.2(10.4)	45.4(10.6)
Age group		
25–40	61 (37.9)	50 (28.6)
41–64	100 (62.1)	125 (71.4)
Sex		
Male	92 (57.1)	111 (63.4)
Female	69 (42.9)	64 (36.6)
Education		
Primary	142 (88.2)	160 (91.4)
Secondary/college	19 (11.8)	15 (8.6)
Marital status		
Married/cohabiting	126 (78.3)	141 (80.6)
Others(single, divorced, widowed, separated)	35 (21.7)	34 (19.4)
Household monthly income		
Low(<mean USD 193.4)	73 (45.3)	82 (46.9)
High(mean USD 193.4)	88 (54.7)	93 (53.1)
Household monthly income		
Mean(SD)* in USD	190.9(164.1)	196.0(150.3)

^ɸ^ Exchange Rate = 1$: Tsh 2265

* T-test

**Table 2:** Distribution of cardio-metabolic risks (primary outcomes), knowledge and attitude (secondary outcomes) of regular street food consumers in Dar es Salaam, by intervention arm (n = 336)-Using T-test.

Characteristics	Interventions	
	T2DM Health information only (IP1), n (%)	T2DM Health information & Plate (IP2), n (%)
Waist circumference(cm)	105.9[104.3, 107.6]	105.0[103.2, 106.8]
Body mass index (kg/m2)	27.5[26.6, 28.3]	27.9[27.0, 27.8]
Diastolic BP (mmHg)	85.4[83.3, 87.5]	86.2[84.1, 88.3]
Systolic BP (mmHg)	127.7[124.3, 131.2]	129.4[125.8, 132.9]
Fasting Blood glucose (mmol/L)	4.2[3.9, 4.4]	4.1[4.0, 4.3]
Fasting Triglycerides (mmol/L)	1.1[1.0, 1.2]	1.2[1.0, 1.3]
Attitude scores (%)	48.0[44.8, 51.1]	49.8[47.1, 52.5]
Knowledge scores (%)	27.9[25.6; 30.1]	30.4[28.0; 32.7]

The end term evaluation conducted after three months of follow up ended up with 336 (175 form IP2 and 161 from IP1) consumers has been shown in **Fig 1**, however, at the enrolment each arm enrolled 280 consumers. A total of 224 consumers were registered as lost to follow up due to several reasons including changing business sites, shifting from Dar es Salaam city, and changing food vendors.

### Effectiveness of the IP2 on cardio-metabolic risk factors of type2 diabetes (pre-post analysis)

GLMM results indicate that diastolic BP was significantly lower in the evaluation phase than baseline AβC = -4.09mmHq (95% CI: -5.42 to -2.76). Fasting blood glucose was significantly higher in the evaluation phase than the baseline phase AβC = 0.39mmol/L (95% CI: 0.23 to 0.55). After adjusting for the interaction between IP2 and age of the consumers, the BMI was significantly lower in the evaluation phase than baseline AβC = -0.7kg/m^2^ (95% CI: -1.17 to -0.23). With interaction between IP2 and income, BMI was higher in the evaluation than baseline phase AβC = 0.73kg/m^2^ (95% CI: 0.08 to 1.38). With interaction between IP2 and income, the waist circumference was significantly higher in the evaluation phase than baseline AβC = 2.07cm (95% CI: 0.15 to 4.00). Considering the interaction between IP2 and age, systolic BP was significantly higher in the evaluation phase than baseline AβC = 2.25mmHq (95% CI: 0.09 to 4.41) (**Table 3**).

**Table 3:** Predicted mean difference estimates of the effectiveness of intervention on cardio-metabolic risk factors using Generalized Linear Mixed Models (GLMM) and Bayesian Modelling for sensitivity analysis: Adjusted models.

Model	Outcome variables	Variables	GLMM Model	
	*Intervention package 2 (IP1 &Plate model)*		*AβC, 95%CI	P-value
Model 1	Body Mass Index (kg/m2)	IP2 (Ref Baseline)	-0.65[-1.10, -0.20]	0.002
		IP2 #Age (Ref Baseline)	-0.7[-1.17, -0.23]	0.001
		IP2 #Income (Ref Baseline)	0.73[0.08, 1.38]	0.013
Model 2	Waist Circumference (cm)	IP2 (Ref Baseline)	-0.97[-2.30, 0.36]	0.078
		IP2 #Income (Ref Baseline)	2.07[0.15, 4.00]	0.018
Model 3	Systolic BP(mmHq)	IP2 (Ref Baseline)	-0.98[-3.08, 1.12]	0.182
		IP2#Age (Ref Baseline)	2.25[0.09, 4.41]	0.021
Model 4	Diastolic BP(mmHq)	IP2 (Ref Baseline)	-4.09[-5.42, -2.76]	<0.001
Model 5	Fasting Blood Glucose (mmol/L)	IP2 (Ref Baseline)	0.39[0.23, 0.55]	<0.001
	*Intervention package (IP1 only)*			
Model 1	Body Mass Index (kg/m2)	IP1 (Ref Baseline)	-0.21[-0.68, 0.26]	0.192
		IP1 #Age (Ref Baseline)	-0.24[-0.71, 0.23]	0.157
		IP1#Income (Ref Baseline)	0.27[-0.38, 0.92]	0.201
Model 2	Waist Circumference (cm)	IP1 (Ref Baseline)	-1.06[-2.51, 0.39]	0.074
		IP1 #Income (Ref Baseline)	-0.07[-1.20, 1.83]	0.487
Model 3	Systolic BP(mmHq)	IP1 (Ref Baseline)	-3.51[-5.78, -1.24]	0.001
		IP1# Age (Ref Baseline)	-1.58[-3.76, 0.60]	0.076
Model 4	Diastolic BP(mmHq)	IP1 (Ref Baseline)	-5.89[-7.34, -4.44]	<0.001
Model 5	Fasting Blood Glucose (mmol/L)	IP1 (Ref Baseline)	0.49[0.31, 0.67]	<0.001

*Adjusted Beta Coefficient (AβC); variables adjusted for include Fixed variable namely age, sex, income, and marital status and two random variables marketplace and participants’ identification number; #:Interaction term; results for interaction was reported if was significant at unadjusted analysis level, Baseline data was treated as reference/base category

### Effectiveness of IP2 on knowledge and attitude (pre-post analysis)

Results from GLMM model showed that positive attitude scores towards NCDs/T2DM prevention strategic measures was significantly higher during evaluation compared to baseline AβC = 18.9%(CI 95%: 15.69 to 22.11). Similarly, knowledge scores on T2DM were significantly higher in the evaluation phase as compared to baseline phase AβC = 13.34% (CI 95%:10.75 to 15.93) (**Table 4**).

**Table 4:** Predicted mean difference estimates of effectiveness of intervention arms on correct knowledge scores (%) and positive attitude scores (%) using Bayesian Modelling for sensitivity analysis.

Models	Outcome variables	Variables	GLMM Model	
	*Intervention package 2 (IP1 & IP2)*		AβC*, 95%CI	P-value
Model 6	Positive attitude scores (%)	IP2 (Ref Baseline)	18.9[15.69, 22.11]	<0.001
Model 7	Correct knowledge scores (%)	IP2 (Ref Baseline)	13.34[10.75, 15.93]	<0.001
	*Intervention package 1 (T2DM health information only)*			
Model 6	Positive attitude scores (%)	IP1 (Ref Baseline)	17.73[14.30, 21.16]	<0.001
Model 7	Correct knowledge scores (%)	IP1 (Ref Baseline)	12.63[9.87, 15.39]	<0.001

*Adjusted Beta Coefficient (AβC); variables adjusted for include Fixed variable namely age, sex, income, and marital status and two random variables include marketplaces and participants’ identification number

### Effectiveness of the IP1 on cardio-metabolic risk factors of type2 diabetes (pre-post analysis)

Systolic and diastolic BP were significantly lower among IP1 consumers in the evaluation than in the baseline AβC = -3.5mmHg (95% CI: -5.78 to -1.24) and AβC = -5.9mmHg (95% CI: -7.34 to -4.44) respectively. Further, fasting blood glucose was higher in the evaluation phase than the baseline phase AβC = 0.49 mmol/L (95% CI: 0.31 to 0.67) (**Table 3**).

### Effectiveness of IP1 on knowledge and attitude (pre-post analysis)

Results from GLMM model showed that positive attitude scores towards NCDs/T2DM prevention strategic measures was significantly higher during evaluation as compared to baseline AβC = 17.73%(CI 95%: 14.30 to 21.16). The results also showed that, positive attitude scores towards T2DM prevention measures was significantly higher in the evaluation phase as compared to baseline phase AβC = 12.63% (CI 95%:9.87 to 15.39) (**Table 4**).

### Sensitivity analysis

Results for effectiveness of interventions on cardio-metabolic risks including overweight/obesity, systolic and diastolic BP, waist circumference and blood glucose as well as attitude towards NCDs/T2DM prevention strategic measures and knowledge scores on T2DM observed from GLMMs were consistent with Bayesian techniques **Appendix V** (**Table A and B**) in S2 File.

## Discussion

Findings from the current pre-post analysis study show that both T2DM health information and subsidized meal with vegetables and fruits, following the principles of a healthy plate model (IP2) and T2DM health information only (IP1), had equal effect. It was observed that age and income were modulating BMI and blood pressure outcomes among participants in IP2. Positive attitude on T2DM prevention measures increased significantly among participants of both arms. Knowledge on risk factors, symptoms, prevention measures, complications and recommended amount of food groups to eat per meal, increased significantly among participants of both arms.

The current study shows that T2DM health information only as well as T2DM health information plus subsidized meal with vegetables and fruits, following the principles of a healthy plate model are effective in reducing cardio-metabolic risk factors including blood pressure, waist circumference and body mass index. Similar effect was also observed from South Africa [56]. Consumption of balanced fruits with moderate glucose and adequate amount of vegetables has been linked to reduction of risks of developing cardio-metabolic risk factors, due to high contents of fibre, folic acid, high potassium, magnesium, vitamin C and flavonoid in them [57–59]. These ingredients improve endothelial function, modulate baroreflex sensitivity, cause vasodilation, and increase antioxidant activities [57–59]. However, a study conducted in Nigeria to investigate effect of fruits and vegetable consumption on cardiovascular risks did not result any significant effect on the risk [58].

To demonstrate portion sizes with the right proportions of each food group in every meal and required ingredients, nutritionists developed the “healthy plate model” [23]. The plate model is a new, innovative idea in Africa for the prevention of NCDs, thus limited knowledge on its importance may function as barrier to compliance, therefore, it may take longer to be adopted and it may require multi-sectorial and multi-disciplinary promotion strategies in different settings and at various levels [60]. To encourage uptake of the plate model, incentives to vendors, such as certificates of recognition to those who will consistently continue to offer food following the plate model, and complete trainings on food nutritional values, can serve as positive re-enforcement [61]. Instituting a plate model in Tanzania is feasible since all plate model components, i.e., foods groups, are readily available in the country [32]. However, vendors do not yet comply with the recommended portion sizes due to costs, individual preference, and culture; hence, T2DM health information is highly required to the community and stakeholders [26]. Continued awareness creation on the benefits of a balanced healthy diet is of paramount importance [61].

Findings from this study show that age and income influenced the outcomes such as BMI and raised BP. Previous studies indicate that higher age and income accelerate cardio-metabolic risks due to long-term accumulation and increased control over resources. This in turn leads to increased risks of exposure to cardio-metabolic risk factors, including excessive alcohol consumption, low physical activity, and consumption of fatty and processed foods [15, 62–65]. The current findings confirm the previous study linked to the current pre-post analysis study which indicated that regular street food consumers who consume recommended/above recommended servings of fruit/vegetables per week had a higher prevalence of overweight/obese than those who were not [15]. A systematic meta-analysis that involved 23 cohort studies, documented an inverse association for intake of some fruits such as, apples and pears, blueberries, grapefruit and grapes and raisins against risks for type 2 diabetes mainly be due to the high glycaemic load (GL) and the added sugar in fruit drinks [66]. The review also documented direct effect of type 2 risks against the intake of fruits such as cantaloupe, brussels sprouts, cauliflower and potatoes. It is therefore, argued that, promotion of fruits consumption should consider glycaemic load.

Many studies have established a direct relationship between obesity and hypertension [19–22]. The current study shows that there was a reduction of both systolic and diastolic BP in both arms. Thus, significant reduction of both systolic and diastolic BP in both arms may partly be explained by the level of decrease in body mass index and waist circumference observed. These findings were in line with those reported in a meta-analysis involving 25 studies, to assess fruit and vegetable consumption and risk of hypertension, concluded that consumption of fruits and vegetables can be inversely associated with the risk of hypertension [67].

This study showed an increase in positive attitude towards prevention measures for cardio-metabolic risks. It has also shown increased knowledge regarding risk factors, signs and symptoms and complications of type 2 diabetes as well as knowledge on the recommended amounts of different food groups. It can, therefore, be argued that the observed positive change on diastolic and systolic BP, BMI as well as waist circumference can be explained by an increase in knowledge and positive attitude which may be was translated into practice, hence influencing behavioral change. Similar findings were observed in a study in South Africa [56].

In this study a negative effect was observed on blood glucose; this is contrary from what was observed in a meta-analysis that involved seven (7) cohort studies. Observed negative glucose results from this study also differ from another meta-analysis which concluded that optimal consumption of fruit or green leafy vegetables significantly reduces T2DM risks [68].

However, the findings documented in this study on glucose may be linked to poor adherence to prevention measures especially at home, where it is usually a challenge to prepare a balanced diet, hence accelerating pre-diabetes [69]. Even though there is increased chance of eating balance diet while an individual is at home, available evidence show that many people confirm that it is exceedingly difficult to avoid overfeeding if delicious foods are plenty [37]. To maximize the effect of intervention, future similar study should consider monitoring individual eating practices while at homes since people are more likely to prepare and consume preference food while at their home hence increase chance of overfeeding [70].

### Limitation of the study

Although this study emphasized the importance of the consumption of a balanced diet while at home, no follow up information regarding the quantity and food groups consumed during weekend days and in evening meals was collected, the focus was on lunch meals consumed to the vendors only and not elsewhere, which are always eaten outside homes. It can therefore be hypothesized that follow up on what participants were eating at home, study would have gained further insights. This study had a considerable rate of loss to follow-up, which could have lowered the power of the study. However, based on the nature of the study design, it remained with a large enough sample size in both arms to allow for meaningful results.

## Conclusion

Pre-post analysis shows that both interventions in this study had positive effects on cardio-metabolic risk factors, knowledge and attitude. However, interactions between IP2, age and income was observed in this study. It is therefore, recommended that designing interventions relating to food should consider combining socio-economic factors. Community awareness creation campaigns regarding the impact of unhealthy eating practices on cardio-metabolic risk factors are recommended.

## Supporting information

S1 Checklist CONSORT 2010 checklist of information to include when reporting a randomised trial*. (PDF)

S1 File (DOCX)

S2 File (DOCX)
