# Supplementary material for: The effectiveness of interventions to reduce cardio-metabolic risk factors among regular street food consumers in Dar es Salaam, Tanzania: The pre-post findings from a cluster randomized trial (Registered by Pan African clinical trial registry with trial # PACTR202208642850935)
Source: PLoS One. 2023 Nov 15;18(11):e0289289. doi: 10.1371/journal.pone.0289289 (PMC10650998; doi:10.1371/journal.pone.0289289)
Supplement: S1 File — (DOCX) [file pone.0289289.s002.docx]

**Effectiveness of prevention package and model for predicting metabolic risk factors of type2 diabetes among street cooked food consumers in Dar es Salaam: A cluster randomized control trial**

**Research Proposal**

**By**

**Gibson Benard Kagaruki**

**Other Co-Investigators**

**Dr. Michael Johnson Mahande**

**Kilimanjaro Christian Medical University College (KCMUCo) of Tumaini University (TU), MOSHI, TANZANIA**

**and**

**Afrique one ASPURE Co- Investigators**

**Prof. Sayoki Godfrey Mfinanga**

**Dr. Esther Ngadaya**

**Prof. Daniel Haydon**

**Prof. Bonfoh Bassirou**

# Table of contents *

Table of Contents

[Table of contents iii](#_Toc513192843)

[Certification v](#_Toc513192844)

[Acknowledgement vi](#_Toc513192845)

[Abbreviations: vii](#_Toc513192846)

[List of Tables viii](#_Toc513192847)

[List of figures viii](#_Toc513192848)

[Operational definitions ix](#_Toc513192849)

[Research Group ix](#_Toc513192850)

[Executive structured summary: x](#_Toc513192852)

[1.0 Introduction and Background: 1](#_Toc513192853)

[1.1 Literature review 2](#_Toc513192854)

[1.2 Problem Statement: 3](#_Toc513192855)

[1.3 Justification of the study: 4](#_Toc513192856)

[1.4 Hypotheses: 4](#_Toc513192857)

[1.5 Objectives 4](#_Toc513192858)

[1.5.1 General objective: 4](#_Toc513192859)

[1.5.2 Specific objectives 5](#_Toc513192860)

[2.0 Materials and Methodology 5](#_Toc513192861)

[2.1 Study area: 5](#_Toc513192862)

[2.2 Study population: 6](#_Toc513192863)

[2.3 Study design: 6](#_Toc513192864)

[2.4 Sample size: 6](#_Toc513192865)

[2.5 Sampling procedures: 7](#_Toc513192867)

[**2.6 Material and method for each specific objective:** criteria. 7](#_Toc513192868)

[**2.6.1 Magnitude of type2 diabetes, behaviourial and its risk factors:** 8](#_Toc513192869)

[2.6.2 KAP on type2 diabetes and its risk factors and complications of the disease: 10](#_Toc513192870)

[2.6.3 Factors associated with adoption and compliance to the food plate mode: 10](#_Toc513192871)

[2.6.4 Effectiveness of intervention package in reducing type2 diabetes risk factors: 10](#_Toc513192872)

[2.6.5 Develop a model for predicting metabolic risk factors for type2 diabetes 10](#_Toc513192873)

[2.7 Implementation of intervention package (health education and plate model) and application of onehealth approach 11](#_Toc513192874)

[2.7.1 Health education 11](#_Toc513192875)

[2.7.2 Plate Model: 11](#_Toc513192876)

[2.8 Project monitoring: 12](#_Toc513192877)

[2.9 Controlling bias and confounders: 12](#_Toc513192878)

[2.10 Expected outcomes: 12](#_Toc513192879)

[2.11 Ethical consideration: 13](#_Toc513192880)

[2.12 Publication of three papers in peer reviewed journals 14](#_Toc513192881)

[2.13 Dissemination plan of research findings: 14](#_Toc513192882)

[Reference 14](#_Toc513192883)

[Appendix A 19](#_Toc513192884)

[Appendix B: 20](#_Toc513192885)

[Appendix C: Consent forms 46](#_Toc513192886)

[Appendix D 54](#_Toc513192887)

# Abbreviations:

ART Antiretroviral Treatment

BMI Body Mass Index

BP Blood Pressure

CVDs Cardiovascular Diseases

DBP Diastolic Blood Pressure

DE Design Effects

FBG Fasting Blood Glucose

FGDs Focus Group Discussions

HDL-C High Density Lipoprotein Cholesterol

HIV Human Immunodeficiency Virus

IDF International Diabetes Federation

KCMUCo Kilimanjaro Christian Medical University College

LDL-C Low Density Lipoprotein Cholesterol

MoHCDGEC Ministry of Health, Community Development, Gender, Elderly and Children

NCD Non communicable diseases

NIMR National Institute for Medical Research

SBP Systolic Blood Pressure

SCFC Street Cooked Food Consumers

SCFV Street Cooked Food Vendors

TC Total Cholesterol

TFNC Tanzania Food and Nutrition Centre

TG-C Triglycerides Cholesterol

TU Tumaini University

WHO World Health Organization

WHP Waist Hips Ratio

# List of Tables

[Table 1 Estimated sample size per each risk factor indicator 16](#_Toc512286115)

[Table 2 Summary for sampling procedure 16](#_Toc512286116)

[Table 3 Inclusion and exclusion criteria 17](#_Toc512286117)

[Table 4 Dependent and independent variables 20](#_Toc512286118)

[Table 5 Timeline and Milestones 22](#_Toc512286119)

[Table 6 Budget for the study project 22](#_Toc512286120)

# List of figures

[Figure 1 shows how primary risk factors interact with intermediate risks 11](#_Toc512286146)

[Figure 2 Shows a Healthy eating plate 12](#_Toc512286147)

[Figure 3 Conceptual framework of risk factor for the intermediate risk factors (metabolic) of type 2 diabetes and prevention and management levels 14](#_Toc512286148)

# Operational definitions

**Metabolic risk factors of type2 diabetes:** Is the group of biological risks including high central obesity (Body Mass Index or waist circumference), blood glucose, reduced HDL, raised Triglycerides and raised blood pressure (1–3).

**Prevention package:** In this study this term “prevention package” includes health education (risk factors, prevention strategies and impact of the disease) and healthy food plate (recommended portion of carbohydrate, fruits, protein vegetables and cooking oils) (4,5).

**Street-vended foods:** Foods or beverages sold after being prepared on the street or at home and ready to eat or to be consumed immediately or later on the street/public places without further processing or preparation(6,7).

**Street food consumer:** Is a person who buys and eat foods or drink beverages prepared in the street or at home and sold in the streets/public areas (6,7).

**Regular street food client:** A person who consume at least three street vended lunches per week ***(to be operationalized and validated later)***

**Effectiveness of intervention:** refers to performance of an intervention in producing the intended outputs/outcomes

**Unhealthy diet**: Meals consumed in a day with less than two servings of fruits, less than five servings of vegetables, more than half plate of carbohydrate and protein(8).

**Unhealthy eating behaviours:** A practice of eating foods or drinks to feel full and satisfied without considering the amount of food or drink if it is appropriate or not (9)**.**

**Healthy food plate**: A plate that guides every person on healthy eating habits showing the recommended proportion and types of foods or drink that are to make up a healthy and balanced diet (4,5)**.**

**Components of Healthy plate:** Half meal vegetables and fruits, ¼ whole grains, ¼ proteins, cooked with healthy plant oils and drinking water(4,5)**.**

# Research Group

The research group comprises of me (student), my supervisors and mentors. University College, Dr. Michael Jonson Mahande^1^ Afrique One ASPIRE program’s supervisors namely Prof. Sayoki Godfrey Mfinanga^2^ (PhD), Dr Esther Ngadaya^2*^ (PhD) and Prof. Daniel Haydon^3^, Prof. Bonfoh Bassirou, these also are co-PI of the program. The mentors include Dr Mary Mayige^2^ (PhD) Dr Suleiman Majige^4^ (PhD) and Dr. Akwilina Wendelin Mwanri^5^. Their institutes of affiliation are indicated below by the corresponding superscript number.

**Institutional affiliation of Supervisors and Advisors**

^1^Kilimanjaro Christian Medical College (KCMC) of Tumaini University (TU), Moshi, Tanzania

^2^National Institute for Medical Research (NIMR),

### ^3^[University of Glasgow](https://www.google.co.tz/url?sa=t&rct=j&q=&esrc=s&source=web&cd=1&cad=rja&uact=8&ved=0ahUKEwjFyLfU1OPTAhWCHsAKHUtrAQ8QFgggMAA&url=http%3A%2F%2Fwww.gla.ac.uk%2Fresearchinstitutes%2Fbahcm%2Fstaff%2Fdanielhaydon%2F&usg=AFQjCNGhMlNmj8KIh2DqpQ8fXeuBLKEfmw&sig2=w8IsfFDGkXbyR3-MQAxO5A)-[Institute of Biodiversity, Animal Health and Comparative Medicine](http://www.gla.ac.uk/researchinstitutes/bahcm/)

^4^The Eastern Africa Statistical Training Centre (EASTC)

^5^Sokoine University of Agriculture

# Executive structured summary:

**Background and rationale:** The world is experiencing unprecedented increase of type2 diabetes attributed to unhealthy diet due low income to afford nutritious food, and low knowledge on nutritional values of food. Other drivers of the type2 diabetes include physical inactivity, harmful alcohol drinking, high bood pressure and high blood glucose, overweight/obese, raised cholestoral, aging and family history of the disease. The significant number of people in urban areas relies on food supplied by *Urban Street Food Vendors* who rarely take into account nutritional value of food thus, putting urban dwellers at risk of getting type2 diabetes. The situation is also compromised by the fact that the available food quality control and enforcement mechanisms focus on monitoring risk factors linked to infectious diseases only. Despite substantial evidence on the burden of the type2 diabetes mellitus; little has been invested on possible prevention interventions to alleviate the problem which is currently imposing exorbitant costs to the country health systems and families.

**Objective**: To implement and evaluate the effectiveness of type2 diabetes metabolic risk factors reduction intervention package and to develop a model for predicting metabolic risk factors among urban street cooked food consumers in Tanzania.

**Methodology:** This cluster randomized control trial will be carried out in urban clusters (market place) treated as control and interventional sites A total of 556 consumers and at least 50 vendors of urban street cooked foods will be recruited. The study will involve the national food regulatory bodies, universities and ward and district experts responsible with food security and environmental safety. The intervention package to be applied to urban street consumers and vendors will include education on Type 2 diabetes and guidance on food intake using the food plate model. The package is composed of health education covering issues on risk factors, symptoms, prevention, complications of diabetes and nutritional values of foods. Education will be delivered through brochures, group education and if possible through mobile phone technology. Another component is a healthy plate model; a plate is composed of recommended potion of carbohydrate, protein, fruits and vegetables plus water/milk and safe cooking oil. The package will be evaluated to assess its effectiveness in reducing metabolic risk factors of type2 diabetes among urban street cooked food consumers. Determinants established during the intervention will be used in developing a model for predicting metabolic risk factors of type2 diabetes among urban street cooked food consumers by using Multivariate/Joint Bayesian modelling techniques. The study findings are envisaged to be used to inform appropriate interventions.

**Keywords:** Type2 diabetes metabolic risk factors, urban street cooked food vendors and consumers, type2 diabetes health education and food plate model.

1.0 Introduction and Background: Type2 diabetes is a global public health problem (10–12). The global prevalence of the disease in 2014 was 8.5% and the number of people living with the disease is expected to increase from 415 million in 2015 to 642 in 2040; a 55% increases if effective intervention programs will not be put in place (12). In Sub Saharan Africa the prevalence is ranging from 1% to 12% while in Tanzania the prevalence of type2 diabetes has increased from less than 1% in 1989 to 9.1% in 2012 (10,11,13,14). According to the global burden of diseases in Tanzania diabetes was among the top 20 killer diseases in 1990 but in 2016 is among the top ten killer diseases (<https://vizhub.healthdata.org/gbd-compare/> **See Appendix A**). Contrary to the middle and high income countries; the prevalence of the diseases by age in the low income countries seems to decrease by increase in age due to premature deaths(12). Type2 is associated with acute complications including kidney failure, amputation, blindness, stroke, impotence plus deaths(11,15).These complications impose cost burden in the health system and at family level (15). In 2012: Diabetes and higher blood glucose caused 3.7 million deaths and 43% of these deaths were premature (11). The risk factors of type2 diabetes are divided in three categories including metabolic/ intermediate, behavioural and socio-economic and familial (16,17). Metabolic risk factors includes raised blood pressure (BP), raised triglyceride (TG), high blood glucose (BG) low high density lipoprotein (HDL) and central obesity(1–3). Physical inactivity, unhealthy diet, smoking and harmful alcohol consumption are the components of behavioural risk factors of the diseases (14,16,17). Socio-economic and familial risk include sex, age, income, location, knowledge and family history of the disease (16–18). The burden of metabolic and behavioural risk in Tanzania is evident; a national STEPs survey of 2012 in Tanzania indicated among that among individuals aged 25-64; 34.7% were overweight/ obese, 26% had raised total cholesterol, 26% had raised blood pressure and 33.8% had raised triglycerides. The survey also showed that tobacco users were 15.9% and alcohol drinkers were 29.3%. Almost all 5680(97.2%) of the participants consumed less than 5 servings of fruit and/or vegetables on average per day (14). Another study conducted in Mwanza among adults; 44.7% were overweight /obese and 36.3% had raised Blood Pressure (19). In Kilimanjaro among participants aged 18 and above 31% had raised blood pressure, 58% were overweight /obese, current drinkers (41.2%) and 21.7% were found with glucose impairment (20). In a recent study in Ifakara nearly 82% of the participants did not meet the recommended daily fruits and vegetables intake (21). Another study conducted in Dar es Salaam and Mbeya among HIV positive clients also reported high prevalence of low HDL (71.9%), raised triglyceride (16.6%), overweight/obese (26.2%), central obesity (46.6%), alcohol consumption (32.9%), low level of fruits and vegetable consumptions (70%) and low physical activity (47.8%) (18).

Most of the studies conducted in Tanzania associated the three groups of risk factors with the likelihood of type2 diabetes. However, evidence indicates that behavioural and socio-economic and familial risks interact to influence the metabolic/ intermediate risk factors (17,22,23). Therefore knowing the influence of the primary risk factors on the intermediate ones may result in a robust outcome on type2 diabetes if one will intervene on the significant primary risk factors. This study aims to implement and evaluate the effectiveness of type2 diabetes metabolic risk factors reduction intervention package and developing a model for predicting metabolic risk factors among urban street cooked food consumers in Tanzania. This kind of intervention package has not yet implemented and evaluated its effectiveness in on reducing metabolic risk factors of type2 diabetes among consumers of urban street cooked food in Tanzania has not been. In additional; no model in place showing which socio-economic and behavioral risk factors which jointly predict the components of metabolic risk factors of type2 diabetes among consumers of urban street cooked food in Tanzania. This project aims to address the gaps above.

## 1.1 Literature review

Figure 1: Primary risk factors interact with intermediate risks


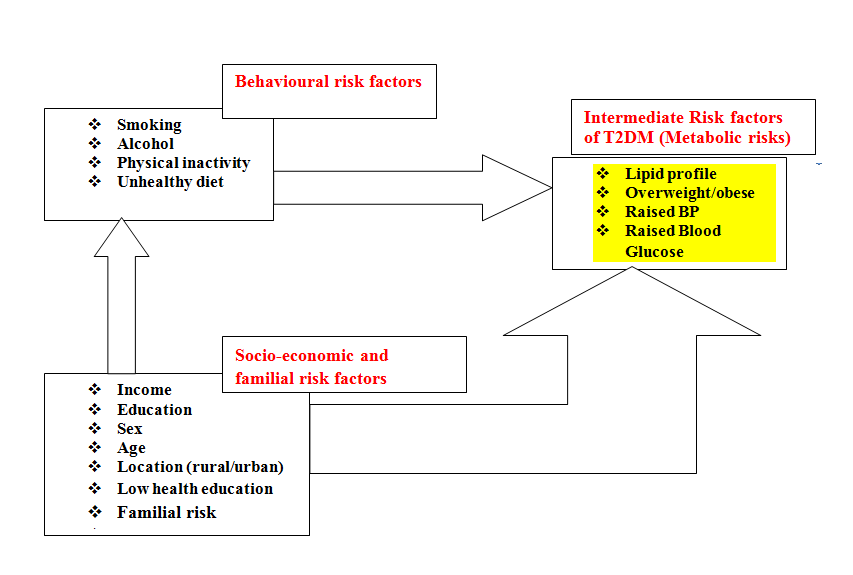


**Adopted from** (17)

Literature show that consuming unhealthy diet i.e. food without adequate intake of fruits and vegetables, food with abundant carbohydrate and protein, fat foods and unmonitored table salt plus minimal physical activities; increase the metabolic risks of type2 diabetes namely high blood pressure, high blood glucose, low high density lipoprotein, triglyceride and central obesity (24,25). In urban settings unhealthy eating pattern is influenced by consumption of street foods which have been reported to be questionable quality in terms of nutritional values (7,26,27). Despite such curiosity, the importance of street food can’t be underestimated as it is estimated that about 2.5 billion or more than 50% of global urban people consume street food(28)**.**

In Tanzania it has been reported that majority of urban people with low and middle income consume street foods especially lunch (26). However, the quality and nutritional values of food served by street vendors is questionable and this has led some countries including Zimbabwe to abolish the services because of increased disease risks linked to the food served (29). In Tanzania street food is composed with low amount of fruits and vegetables; big portion of carbohydrate and protein (26,27). This may be due to the majority of street vendors having limited capital and geared to maximizing profit regardless of the quality and nutritional values of food they sell to clients. Limited capital also hinders them from undergoing food related formal trainings, buying safe food, serving nutritional food i.e. food with adequate vegetables, fruits and cooked with safe oil (vegetables oils) (30–32). Other studies had also indicated that cultural, environmental and economic factors affect eating habits, foods availability and accessibility(33–36). For example Masai tribe consider vegetables as animal foods, in Dar es Salaam findings indicated that preference and high costs were hindering factors for vegetables and fruits consumption(26,27,34). Furthermore, available food quality regulatory practices focus on infectious diseases leaving issues related to food nutritional values unmonitored. Despite increased burden of diabetes and its risk factors in our country; little has been done to reduce the burden contributed by street foods. The prevention package (health education and healthy plate) will contribute in reducing behavioural and metabolic risks factors. The package is composed of health education covering issues on risk factors, symptoms, prevention, complications of diabetes and nutritional values of foods. Education will be delivered through brochures, group education and if possible through mobile phone technology. Another component is a healthy plate model; a plate is composed of recommended potion of carbohydrate, protein, fruits and vegetables plus water/milk and safe cooking oil. Evidence indicate that consuming recommended potion of fruits and vegetables, carbohydrate and protein coupled with physical activity decrease metabolic risk factors and up to 90% cases of diabetes can be prevented(16,22,23,37).

The model was developed with the aim of providing detailed simple guidance to help people make the best eating choices(38) . Studies have demonstrated that the cohort which adhered and complied with the Healthy Eating Plate guidelines had lower risks of heart disease and premature death (4,5). Effectiveness of the model can’t be underestimated as studies have demonstrated that the cohort which adhered and complied with the Healthy Eating Plate guidelines has lower risks of heart disease and premature death(4,5). A similar kind of model is also advocated by the Tanzania Food Nutrition Centre (TFNC) in the community with the aim of mobilizing people to eat food with acceptable nutritional values i.e. food with recommended standard ratio of vegetables, fruits, proteins and carbohydrates. However, this may be feasible for the meal prepared by individual family at home but difficult to implement when taking meal outside home since in most cases serving recommended ratio of vegetables and fruits adds costs to the food vendors especially the street food vendors who make meagre profit because of limited capital for investment and low price tied to food plate as most of their clients are poor(30–32).

Figure2: A Healthy eating plate model


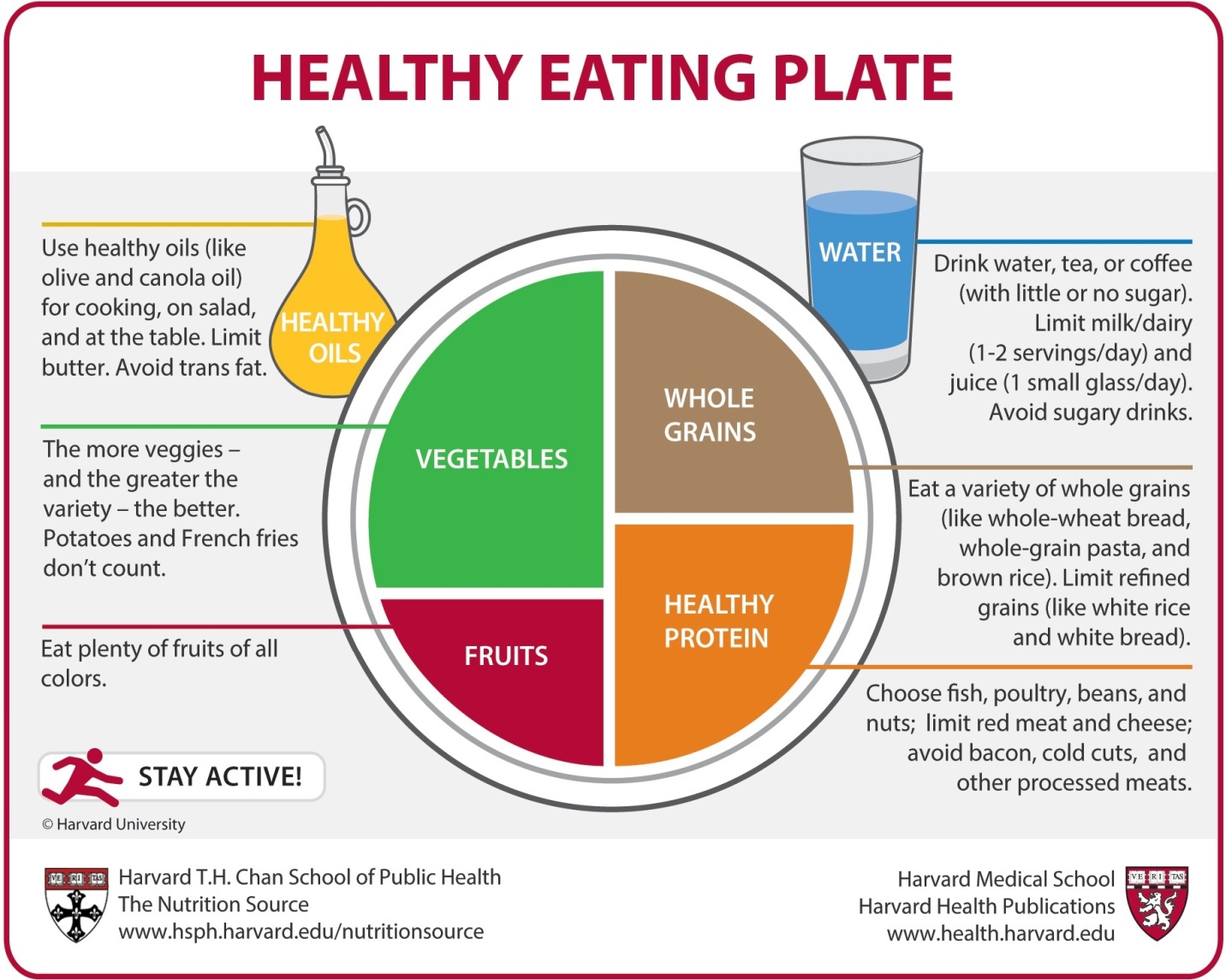


- 1. Problem Statement: The global, regional and local burden of type2 diabetes and its metabolic risk factors is currently increasing (10,12,14,18,20). At the same time; more than 50% of the global an about 50% Sub-Shaharan Africa population and majority of low and middle income of urban dwellers in Dar es Salaam consume food prepared and sold in the streets (26,39). Evidence indicate that vendors rarely serve a healthy plate because of low knowledge on food nutritional values, limited capital and incompetent food quality control mechanisms(26,27,40). Despite the increased burden of T2DM and its metabolic risk factors; the effectiveness of the current prevention package has not yet been piloted and tested in Tanzania especially among street vendors and consumers and there is no model in place to predict the risks burden. Such package is worthy to be tested in Tanzania as evidence indicate that consuming recommended potion of fruits and vegetables, carbohydrate and protein coupled with physical activity decrease metabolic risk factors and up to 90% cases of diabetes can be prevented (16,22,23,37). Testing this package will come up with information helpful in improving the quality of services provided by food vendors and avoid abolishing this sector as has happened in Rwanda and Zimbabwe because of increased disease risks (29). In addition, there is no published analyses of Tanzanian data showing how behavioural and socio-economic risk factors predict metabolic risk factors together using advanced generalized linear mixed models fitted with both frequentist and Bayesian frameworks.Therefore, this intervention study is focusing on food nutritional values and healthy eating behaviours will contribute to reducing metabolic risk factors of type2 diabetes and consequently contributing to reducing the chance of developing type2 diabetes among consumers of urban street cooked foods.

1.3 Justification of the study: Evidences indicate that food vendors have limited knowledge on foods nutritional values and have limited capital to supply healthy plate. Though many studies report higher prevalence of diabetes risk metabolic risk factors; the status of risks among those who regularly consume street foods is not known and in place there is no model to predict the metabolic risk factors among this group who are increased risk to experience the problem. This study therefore, will document the contribution of street foods on metabolic risk factors. At the end of this study; the impact of prevention package (health education and healthy plate model) on metabolic and behavioural risk factors will be known. A Joint/ Multivariate Bayesian Model for predicting metabolic risk factors for T2DM will be also developed. The research also will inform academic world, decision makers, policy makers and practitioners on what is transpiring regarding type2 diabetes in relation to cooked food supplied by street vendors. Academically, the research is important in the field of epidemiology especially on the contribution of urban street cooked food on metabolic risk factors of type2 diabetes. Furthermore, findings will help to enhance and enrich the policy and guidelines for food regulatory authorities in Tanzania. Ultimately, the research findings will help to improve food system in urban areas.

1.4 Hypotheses: The following two hypotheses will be tested in this study:

1. A prevention package is effective in reducing metabolic risk factors of type2 diabetes among consumers of urban street cooked food in Tanzania
2. Determinants established during the intervention will be useful in developing the best model for predicting metabolic risk factors of type2 diabetes among consumers of urban street cooked food in Tanzania

## 1.5 Objectives

1.5.1 General objective: To implement and evaluate the effectiveness of type2 diabetes metabolic risk factors reduction intervention package and to develop a model for predicting metabolic risk factors among urban street cooked food consumers in Tanzania.

### 1.5.2 Specific objectives

1. To determine the magnitude of metabolic and behavioural risk factors of type2 diabetes among urban street cooked food consumers
2. To assess knowledge and attitude on type2 diabetes among consumers and vendors of urban street cooked food,
3. To determine the socio-economic and cultural factors associated with the adoption of the intervention package,
4. To assess the effectiveness of health prevention package in reducing metabolic risk factors for type2 diabetes consumers of urban street cooked food and
5. To develop a model for predicting metabolic risk factors for type2 diabetes among consumers of urban street cooked food

Figure 3 Conceptual framework of risk factor for the intermediate risk factors (metabolic) of type2 diabetes and prevention and management levels

**
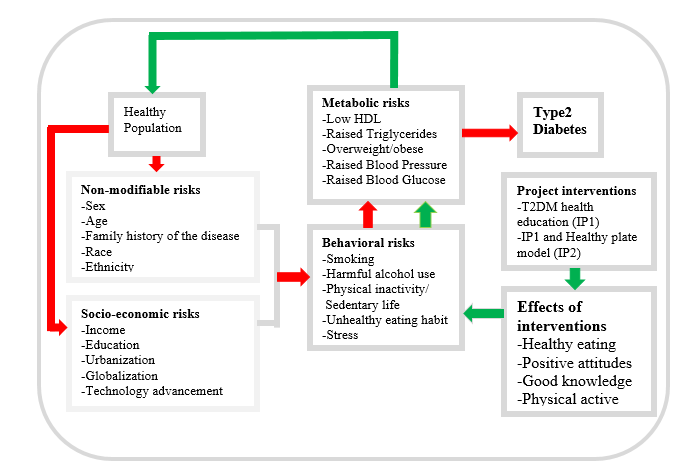
**Adopted from (12)

# 2.0 Materials and Methodology

2.1 Study area: This study will be conducted in Dar es Salaam region. Dar es Salaam is the largestcity in Tanzania with land area of 538km^2^. It has five municipalities namely Ilala, Kinondoni, Temeke, Ubungo and Kigamboni. The region/city contributes about 80% of all national economy thus attracting more immigrants from inside and outside of the country for economic investments. The city is the third fastest-growing in Africa and the ninth fastest-growing in the world. According to the national census report of 2012 the population of Dar es Salaam was 4.36 million (41)**.** The population of the region is expected to over double up to 10.76 million by 2030(42). This study will be conducted in three already randomly selected districts and six market places (two per district). The selected districts are Ilala, Kinondoni and Ubungo. The region was considered for this study because of previous researches which indicated that majority of low and income people in the area consume street foods served by street vendors(26,27). The studies also indicated that the nutrition value of the foods supplied by street food vendors is questionable as they normally serve smaller amount of fruits and rarely served fruits due to limited capital and low knowledge on nutrition values of foods (26,27)**.** Such eating behaviours expose the consumers at risk of getting type2 diabetes due to increased risk of metabolic risk factors. In Dar es Salaam it was also observed that costs and attitude were the factors associated with low consumption of fruits and vegetables among pupils and student (27). Another study conducted in Dar es Salaam among HIV positive people indicated that consuming junk foods led to increased body weight which was perceived a strategy for increasing CD4 counts (43)**.**

2.2 Study population: Urban regular eaters of street cooked food, vendors and key stakeholders. Key stakeholders will include health environmental officers, municipal nutritional officers and officers from the national food regulatory authorities.

2.3 Study design: This study will be intervention (experimental) with two arms control and experimental clusters. This design is appropriate for this study since it will allow researchers to measure the effects of an intervention package (health education and plate model) by comparing the outcomes of the package in the experimental group versus control group which will receive a placebo (health education) after three months of follow up. Healthy plate will contain ¼ carbohydrate, ¼ protein, ¼ vegetables and ¼ fruits. Health education will focus on risk factors of type2 diabetes, prevention strategies and complications of the disease and nutritional values of foods. A proposed intervention package will be assigned to the clusters (market places) randomly but subjects in the intervention sites will not be blinded in order to get the desired outcomes and justify the efficacy of an experiment. Before intervention, baseline information including behavioural risk factors (physical inactivity, unhealthy diet, and smoking, harmful alcohol consumption), metabolic risks (raised blood pressure, triglyceride, blood glucose, low HDL and central obesity) and socio-economic and familial risks (sex, age, income, location, knowledge, family history of diabetes) will be collected from both arms.After intervention the same information collected at the baseline will also be collected at the end line.

2.4 Sample size: The sample was calculated by considering the following statistical parameters: level of significance (Z_1-α_), power of the test (Z_1-β_), population standard deviation (δ) and population variance (δ^2^).The baseline indicator for the mean waist hips ratio of µ_0_ =0.82 and a target of reducing the mean of waist circumference by 5% after an intervention i.e. from 0.82 to µ_a_=0.78. A design effects (DE) of 1.5 to clear variance between clusters was applied and none response rate of 20%. With the above statistical parameters then an actual sample size is 278 (**Table 1**). This sample size will be doubled to include both arms (intervention and control). Furthermore, at least 50 food vendors will be recruited and each food vendor will be asked to identify their regular clients who will be approached for the study.

Sample size (n) calculation formula (44).

**
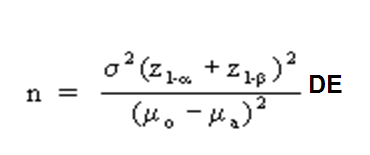
**

Table 1 Estimated sample size per each risk factor indicator

| **S/No** | **Parameter** | **µ_o_** | **Δ** | **δ^2^** | **Desired % change in mean after 3 months** | **µ_a_** | **DE** | **Non response (20%)** | **N** |
| --- | --- | --- | --- | --- | --- | --- | --- | --- | --- |
| 1 | BMI(Kg/m^2^) | 22.9 | 4.6 | 21.2 | 5 | 21.8 | 1.5 | 0.8 | 204 |
| 2 | TC(mmol/L) | 4.6 | 0.78 | 0.6 | 5 | 4.4 | 1.5 | 0.8 | 178 |
| 3 | TG(mmol/L)* | 1.7 | 1.1 | 1.2 | 12 | 1.5 | 1.5 | 0.8 | 353 |
| 4 | SBP (mmHg) | 129 | 21.3 | 453.7 | 5 | 122.2 | 1.5 | 0.8 | 129 |
| 5 | DBP (mmHg) | 80.3 | 12.2 | 148.8 | 5 | 76.3 | 1.5 | 0.8 | 109 |
| 6 | FBG(mmol/L) | 4.8 | 1.6 | 2.6 | 10 | 4.3 | 1.5 | 0.8 | 120 |
| 7 | WHR (none) | 0.82 | 0.2 | 0.0 | 5 | 0.779 | 1.5 | 0.8 | 278 |

***Source:*** (14)

2.5 Sampling procedures: This study will also apply a multistage cluster sampling whereby; three out of five districts have been selected randomly from Dar es Salaam region. Thereafter, mapping of market places (clusters) will be done and in each selected district two market places will be randomly selected then randomized one to receive an intervention package (health education and plate model) and another to receive a placebo (health education) . The step will be followed by mapping of food vendors to identify eligible food vendors and form a sampling frame per each cluster. Thereafter, probability proportion to size will be done to determine the number of vendors to be recruited per each cluster. Food vendors will be also selected randomly from each cluster to participate in the study. Each selected food vendor will identify regular customers then consent to participate in the trial will be sought from all identified clients (**Table 2**).

Table 2 Summary for sampling procedure

| **Stage** | **Ilala** | **Ubungo** | **Kinondoni** |
| --- | --- | --- | --- |
| 1. Identify the market places(MP)/clusters | 5 | 9 | 8 |
| 2. Identify and match eligible MPs | 5 | 9 | 8 |
| 3. Select randomly the required number of MPs for the study | 2 | 2 | 2 |
| 4. Assign MPs randomly into control and intervention arms | 2 | 2 | 2 |
| 5. Mapping Food vendors(FVs) | xx | Xx | Xx |
| 6. Identify eligible FVs | xx | Xx | Xx |
| 7. Use probability proportion to size (PPS) to allocate ≥50 FVs | xx | Xx | Xx |
| 8. Mapping of eligible* regular clients per FV | xx | Xx | Xx |
| 9. Seek consent and recruits all regular clients /FV | xx | Xx | Xx |

2.6 Material and method for each specific objective: A tool for data collection have been adopted from WHO STEP Survey questionnaire and other studies on similar topic(14,45–51). This tool will be then translated from English to Swhaili version. A tool also will consist of information including socio-demographic, behavioural, physical measurements and biological tests. A total of three focus group discussion (FGDs) involving street food vendors and six in-depth interviews with key stake holders will be conducted to assess their level of knowledge on type2 diabetes and hypertension risk factors and factors associated with adoption of food plate model and education package. Eight in-depth interviews will be conducted among public staff responsible with setting enabling and regulating working environment of food vendors. A three days training will be made to research assistants, this will involves orienting them on the study protocol, data collection tools and study ethics. **Table 3** shows the inclusion and exclusion criteria.

Table 3 Inclusion and exclusion criteria

| **Type of respondent** | **Inclusion** | **Exclusion** |
| --- | --- | --- |
| Street Food consumers | 1. Consuming at least three lunches meals/week to the same street food vendor (SFV) 2. Willing to participate in the study 3. Will remain in the study area for the duration of at least six months 4. Aged 25-64 years | Pregnant or lactating mothers |
| Street food vendors | 1. Vending food in the current site for not less than 6 months 2. Has at least 10 clients who have been consuming a least three lunch meals per week for not less than three months 3. Ready to implement the components required for an intervention model 4. Will remain vending food at same site for not less than six months 5. Aged 18 and above years | Not providing services daily |

2.6.1 Magnitude of type2 diabetes, behaviourial and its risk factors: The prevalence of type 2 diabetes among urban street cooked food consumers will be determined. A person will be considered to have diabetes if the reading for fasting blood glucose measured using Accu-check machine is >7.0mmol/L or is on medication for raised blood glucose. This study will also assess the prevalence metabolic and behavioural risk factors of type 2 diabetes. Prevalence of metabolic risk factors will be assessed as follow: being overweight or obese (BIM≥25Kg/M^2^), raised TC (≥5.1mmol/L), raised TG (≥2 mmol/L), low HDL (<1.03 mmol/l for Male and < 1.29 for Female), raised LDL (>2.6 mmol/L), Abnormal Waist Hips Ratio for male and female if the value is >0.8 and >0.9 respectively, raised Systolic BP (≥140mmHg) and Diastolic BP (≥90 mmHg) (17,18). Behaviorial assessement will including participating in physical activities as per recommended standard (≥600MET/week), Behaviourial assessment will include consumption of fruits and vegetables as per standard (≥5 days per week), non-harmful drinking (5 or less for male and 4 or less for female units of alcohol per week) and not consuming table salt will be assessed (18,52).

**Blood Pressure (BP) Measurements**: Blood pressure will be measured while patients are in a sitting position, using an M4 Omron® device. An average of the last two readings taken three times consistently after three minutes from the left arm will be treated as final reading and will be used during analysis(18). The final will be classified as raised blood pressure if BP of ≥140/90mmHg.

**Weight Measurement**: SECA® weighing scale will be used to measure weight. Participant will be asked to remove any heavy clothing (such as coats and shoes) and stand still on the weighing scale, with hands by their sides.

**Height Measurement:** Height will be measured with a SECA® stadiometer, while a patient is facing directly ahead. Patients will be instructed to remove their shoes, caps or head scarf, keep their feet together, and stand with their arms by the sides. Measurement will be taken with heels, buttocks and upper back in contact with the stadiometer.

**Body Mass Index (BMI):** The product of weight (in Kilograms) divided by height squared (m^2^) will give BMI. Categorization BMI will be as follows ; obesity is ≥30 kg/m2; Overweight if the BMI is > 25 but < 30kg/m2, and normal if the BMI is between 18-25kg/m2 and underweight if below 19 kg/m^2^ (53).

**Waist circumference:** The measurement will be done according to WHO guidelines where by measurement will be taken at the approximate midpoint between the lower margin of the last palpable rib and the top of the iliac crest (54). The values will be grouped as follows abnormal waist circumference of male and female are >94 cm and >80 cm respectively ; this classification is according to World Health Organizations (WHO) and International Diabetes Federation (IDF) recommendations(1,3).

**Hips measurements:** Hip measurement is one of an important parameter for monitoring weight gain or loss. Participant will be asked to remove heavy garments and put the feet together then soft measuring tape will wrapped and snag around the widest part of his/her hips. Then the value of hip measurement is the point at which the end of the tape meets the remaining length.

**Waist Hips Ratio**: Waist to hip ratio is a measure of how fat is distributed around the body. It is calculated by dividing waist to hips values. Waist Hips Ratio will be considered normal for male and female if the value is ≤0.8 and ≤0.9 respectively(55).

**Laboratory Tests**: Study participants will be requested to return to the clinics to provide fasting blood samples for blood glucose and lipid profile (TC, HDL and Triglyceride) tests in the following day after fasting for at least 8 hours. The tests will be performed by trained laboratory technicians.

**Fasting Blood Glucose:** Blood sample will be taken by finger prick (capillary) and measured using Accu-check machine. Raised fasting blood glucose will be considered if the result is >7.0mmol/L (19,20)**.**

**Lipid profile testing:** Blood sample will be collected for testing HDL-C, HDL-C, TC, and TG-C. Blood will be kept at room temperature and will be analyzed the same day using fully automated biochemistry analyzers by the direct end point enzymatic method.

**Alcohol drinking**: Participants will be interviewed on the history of alcohol drinking and those who will be found currently drinking will be asked further questions. These questions will include age when started drinking, frequency of drinking and amount taken on average when they drink.

**Smoking:** Participants will be interviewed on the history of smoking and those who will be found currently smoking will be then asked further questions. These questions will include age at initiation of smoking and age when started smoking daily. They will be also responding on questions regarding number of cigarettes the smoke and the frequency of smoking.

**Fruits/vegetable consumption:** Fruits and vegetables consumptions will be assessed as per WHO standards. Participants will be asked to responds on how many days per week they eat vegetables and fruits and estimating average amount of servings they consume in each event.

**Physical activities:** Physical activity will be assessed using the global physical activity questionnaire (GPAQ). This questionnaire collects information on physical activity participation in three settings including activity at work (work or occupational physical activity), travel to and from places (Active travel) and on leisure time physical activity (Recreational physical activities) as well as sedentary behavior. The amount spent doing physical activity will be quantified using Metabolic Equivalent of Task (MET), which is the ratio of a person's working metabolic rate relative to the resting metabolic rate. One MET is defined as the energy cost of sitting quietly, and is equivalent to a caloric consumption of 1 kcal/kg/hour. It is estimated that, compared to sitting quietly, a person's caloric consumption is four times as high when being moderately active, and eight times as high when being vigorously active. Thus during calculating a person's overall energy expenditure 4 METs were assigned to the time spent in moderate activities, and 8 METs to the time spent in vigorous activities. Low levels of physical activity will be defined as < 600 MET-minutes per week and high levels of physical activity as ≥3000MET minutes/week (14,52).

### 2.6.2 KAP on type2 diabetes and its risk factors and complications of the disease:

A list of questions will be adopted from other generic tools to assess the level of knowledge among urban street food consumers on risk factors, prevention measures and complications of type2 diabetes ( Parmenter and Wardle, 1999; Shah, Kamdar and Shah, 2009; Barzegari *et al.*, 2011; Bano *et al.*, 2013; WHO, 2013; Mumu *et al.*, 2014; Ahmad and Ahmad, 2015; Fatema *et al.*, 2017; Yang *et al.*, 2017**)**. Another list of questions will be developed to capture information on positive and negative attitudes towards change in favour of reducing metabolic risk factors of type2 diabetes. The study also will assess the level of positive practice in relation to reducing the chance of developing metabolic risk factors of type2 diabetes.

2.6.3 Factors associated with adoption and compliance to the food plate mode: This study will also assess enabling factors (socio-economic, cultural and environmental factors) which in one way or another will influence adoption of plate model. This information will be collected from food vendors, consumers and other stakeholders (nutritionists, health environmental officers). This information will be collected using interview questionnaire and interview guide for FGDs and in-depth interview.

2.6.4 Effectiveness of intervention package in reducing type2 diabetes risk factors: At the end of an intervention the mean comparison using independent T-test of all metabolic risk factors for T2DM will be made between the control and intervention groups. For pre-post analysis, the study will apply Generalized Linear Mixed Models (GLMM) to accommodate fixed and random variables. Sensitivity analysis will be conducted to assess if variables which will be statistically significant by GLMM model, will be consistently significant using the Bayesian Modelling technique with Markov Chain Monte Carlo (MCMC). Post and baseline analysis will also employ both linear and logistic regression to assess the factors associated with both primary and secondary outcome of interest. The following are the metabolic risk factors which will be assessed: High Density Lipoprotein Cholesterol (HDL-C), Low Density Lipoprotein Cholesterol (LDL-C), Total Cholesterol (TC), and triglycerides Cholesterol (TG-C) Body mass index (BMI), Waist Hips Ratio (WHR) and Blood Pressure (BP) (4,5,22,23,47,49), these will be our primary outcome of interest. The study will also assess mean knowledge and attitude scores as our secondary outcomes, mean days of fruits/vegetable consumption per week, mean number of cigarettes smoked per week, drinks consumed per week and mean MET/week between the control and intervention sites. Frequency of alcohol consumption and smoking will be also compared between the control and intervention sites. Lastly, the study will also compare the proportion of quitting alcohol consumption and smoking tobacco products between the control and intervention sites.

2.6.5 Develop a model for predicting metabolic risk factors for type2 diabetes: After collecting the endline data then a model will be developed using Multivariate/Joint Bayesian techniques to assess socio-economic and behavirioal risk factors which influence each metabolic risk factors and those which influence these factors jointly. **Table 4** below shows the type of predictive/explanatory factors which will be modelled per each metabolic risk factor of T2DM(16).

Table 4 Dependent and independent variables

| **Dependent variables- Metabolic Risk factors of T2DM** | **Independent variables/confounders** |
| --- | --- |
| 1. Body Mass Index or Waist Circumference 2. Fasting Blood Glucose 3. High Density Lipoprotein 4. Triglyceride 5. Systolic BP 6. Diastolic BP | **Socio-economic and demographic risks:**  Marital status, Education, Ethnicity, Employment status, Sex and Age  **Lifestyle behaviours**  Family history of diabetes  Smoking: Never/previously/currently; Frequency of smoking  **Physical activities**  Interview methods will be used in measuring:  Type, frequency and intensity, Work-related, communing and leisure physical activities  **Dietary pattern:**  Vegetables, Fruits, Spreads and oil, Table salt & Alcohol consumption  **Others factors:**  Mean number of health plate consumed per week,  Type2 diabetes knowledge of vendors and consumers |

## 2.7 Implementation of intervention package (health education and plate model) and application of onehealth approach

2.7.1 Health education**:** To achieve the goals of this study, application of one health approach whereby different disciplines are involved in interdisciplinary manner to implement the project became inevitable. Participants will be educated on the behavioural, familial and metabolic risk factors for T2DM. The participants will also be educated on the prevention measures of T2DM. These measures will include recommended level of MET/Week of physical activities, recommended amount of alcohol to drink per week for each sex, the recommended amount of fruits and vegetable servings per day. In addition, the participants will be educated on the danger of smoking and eating excess fats, carbohydrate and fruits with high calories. The signs and symptoms of T2DM and complication of the diseases will be also explained to the participants. This part of intervention will be implemented by nurses working in diabetes clinic and providing dietary health education to diabetes patients. Nutritionist and health environmental officers will be part of the team since they work closely with the food vendors. If the findings from this study will be used to inform the food policy, guidelines and food systems, these officers will be responsible for monitoring compliance and adherence to the new changes.

2.7.2 Plate Model: Before implementing the food plate model the consumers and vendors who will consent to be recruited for the intervention arm will be educated on how to prepare the standard and recommended food plate model and its hypothesized role of reducing metabolic risk factors of T2DM. We envisaged that knowledge about the current topic will influence practice and increase uptake of an intervention. The benefits of serving the food plate model will be explained to the vendors and these benefits will include serving small amount of carbohydrate, getting extra business by selling separate portion of vegetables and fruits after the project implementation thus getting more profit. For the consumers the benefits of plate model will include paying reduced price for carbohydrate since amount to be served will be the quarter of the normal served amount. Other advantages will include preventing the consumers from excess weight gain and contributing to balancing lipid T2DM metabolic risk factors. More emphasize will be given to the participants regarding the recommended ratio of fruits, vegetables, fats/protein and carbohydrate. Participants will be also instructed on how to ensure that the food plate model contain the recommended amount of potassium, magnesium and calcium levels. The food plate model will consists of more fruits and vegetables which will be half normal eaten plate before the intervention and each quarter will comprise carbohydrate and fats/protein. The participants will be insisted to avoid diary with high saturated fats and cholesterol.

2.8 Project monitoring: To ensure sustainability of the outcomes; the project will involve municipals officers during the implementation of the study so that they take lead in promoting supply and consumption of healthy diet. Intervention program will take three months(57). Monitoring will be done to ensure adherence and compliance as per study protocol requirements. This will involve signing a daily healthy plate form and weekly visits (intervention arm) while a daily lunch form and weekly visit (control arm). The main targets indicators will include mean of metabolic components (systolic and diastolic blood pressure, fasting blood glucose, body mass index, waist circumference, triglycerides and high density lipoprotein), proportion of respondents with high knowledge and positive attitude before and after intervention.

2.9 Controlling bias and confounders: This project is prone to bias since food vendors will be responsible in identifying their regular customers a situation which is associated with selection bias, however, the vendors will be told that all identified clients will be approached to consent for the study. Interview bias which is associated with incomplete and inaccuracy data; this effect will be minimized by training data collectors and field supervision(58–62). This study is also prone to known and unknown confounders. Clusters randomization (market place), probability proportional to size allocation of vendors and multivariable analysis approaches will minimize confounding effects (58–62)**.**

2.10 Expected outcomes: The magnitude of type2 diabetes and its risk factors among consumers of urban street cooked food will be known. The level of knowledge and attitude on type2 diabetes and its risk factors among consumers and vendors of urban street cooked food will be known. Through this study the effects of health intervention (Health education and healthy eating plate model) on prevalence of metabolic risk factors of type 2 diabetes will be known. A model for monitoring and predicting metabolic risk factors of type 2 diabetes among consumers of urban street cooked food will be developed from this study. Consequently, the study findings will help to enhance and enrich the policy and guidelines for food regulatory authorities in Tanzania and elsewhere. Ultimately, the research findings will inform food policy and food related guidelines and will also help to improve food system in urban areas.

Table 5 Timeline and Milestones

| **Activity** | **Oct 2017-**  **Sept 2018** | | | | **Oct 2018-**  **Sept 2019** | | | | **Oct 2019-**  **Sept 20120** | | | |
| --- | --- | --- | --- | --- | --- | --- | --- | --- | --- | --- | --- | --- |
|  | **Q1** | **Q2** | **Q3** | **Q4** | **Q1** | **Q2** | **Q3** | **Q4** | **Q1** | **Q2** | **Q3** | **Q4** |
| 1. Writing proposal |  |  |  |  |  |  |  |  |  |  |  |  |
| 2.Application of Ethical Approval |  |  |  |  |  |  |  |  |  |  |  |  |
| 3. Attend monthly or quarterly supervisory meetings |  |  |  |  |  |  |  |  |  |  |  |  |
| 4.Attend at least 4 short courses & 5 conferences |  |  |  |  |  |  |  |  |  |  |  |  |
| 5.Write small proposals to mobilize l funds |  |  |  |  |  |  |  |  |  |  |  |  |
| 6. Write systematic review /meta analysis |  |  |  |  |  |  |  |  |  |  |  |  |
| 7.Site preparation |  |  |  |  |  |  |  |  |  |  |  |  |
| 8.Finalize protocol |  |  |  |  |  |  |  |  |  |  |  |  |
| 9.Supervisory and Mentorship |  |  |  |  |  |  |  |  |  |  |  |  |
| 10.Baseline data collection |  |  |  |  |  |  |  |  |  |  |  |  |
| 11.Implement intervention package |  |  |  |  |  |  |  |  |  |  |  |  |
| 12.Data management and analysis for baseline |  |  |  |  |  |  |  |  |  |  |  |  |
| 13.Defend Thesis |  |  |  |  |  |  |  |  |  |  |  |  |
| 14. End line data collection |  |  |  |  |  |  |  |  |  |  |  |  |

2.11 Ethical consideration: An ethical clearance will be sought from the Medical Research Coordination Committee of the National Institute for Medical Research. Regional and district authorities will be informed about the aim and procedures of the study. During the study, participants will be informed about the aims and benefits of the study and a written informed consent will be obtained from each participant. Names will not be used and confidentiality will be maintained by keeping data collection questionnaire locked in a secure cabinet, while the electronic data file will be kept in a password protected computer. Participants who will be examined and identified with hypertension, or diabetes (high blood sugar) will be referred to the nearest health facilities for further management. Those who will be screened and found with abnormal cholesterol and body mass index will be counselled and advised on proper approaches for risk prevention and control. This protocol will be registered in order to comply with WHO’s  [Helsinki](http://www.wma.net/en/30publications/10policies/b3/index.html) Declaration which states that "Every clinical trial must be registered in a publicly accessible database before recruitment of the first subject".

Table 6 Budget for the study project

| **S/No** | **Item** | **Description** | **Year1 (Euros)** | **Year2 (Euros)** | **Year3 (Euros)** | **Total (Euros)** | **Source** |
| --- | --- | --- | --- | --- | --- | --- | --- |
| 1 | Salary/Stipend | 36 months | 4,560 | 4,560 | 4,560 | **13,680** | ASPIRE |
| 2 | Tuition Fees |  | 5,200 | 5,121 | 5,120 | **15,442** | ASPIRE |
| 3 | Training / Workshop | Pm | - | - | - | **-** | Vliruos/others |
| 4 | Supervision | Lump sum | 381 | 381 | - | **762** | ASPIRE |
| 5 | Research cost (lab) | Lump sum | 19,650 | - | - | **19,650** | ASPIRE/others |
| 6 | Equipment(lab) | Lump sum | 762 | - | - | **762** | ASPIRE/others |
| 7 | Travel | Lump sum | 250 | 250 | - | **500** | ASPIRE |
| 8 | Miscellaneous | Lump sum | 300 | 300 | - | **600** | ASPIRE/others |
|  | **Total** |  | **31,103** | **10,612** | **9,680** | **51,396** |  |

|  | **Explanation/details** |
| --- | --- |
| 1 | Meal, Accommodation, fare |
| 2 | University fee and other costs including examination fee, medical costs, application fee, stationeries, book allowance, thesis production etc |
| 3 | Short courses, seminars and workshop |
| 4 | Supervision by academic supervisor |
| 5 | Salaries, car hire, reagents and supplies etc |
| 6 | Glucometer, scale, height body, tapes measure, BP machines |
| 7 | Bus ticket, tax charges and accommodation |
| 8 | All emergency expenses |

## 2.12 Publication of three papers in peer reviewed journals

A least three papers are expected to be published before completion of the PhD program in the peer review and international journal.

**Titles:**

1. Magnitude of metabolic and behavioral risk factors of type2 diabetes among urban street cooked food consumers from selected three districts in Dar es Salaam region
2. Knowledge and attitude on type2 diabetes among consumers and vendors of urban street cooked food from selected three districts in Dar es Salaam region
3. Assessment of socio-economic and cultural factors associated with adoption and compliance to the food plate model among consumers and vendors of urban street cooked food from selected three districts in Dar es Salaam region
4. Effectiveness of health prevention package in reducing metabolic risk factors for type2 diabetes consumers of urban street cooked food from selected three districts in Dar es Salaam region
5. A Bayesian model for predicting metabolic risk factors for type2 diabetes among consumers of urban street cooked food

2.13 Dissemination plan of research findings: Data emanating from this study will be presented and discussed with official from the Ministry of health and social welfare, Tanzania Diabetes Association, Afrique One ASPIRE, KCMUCo Academic forum and other stakeholders to identify feasibility of utilizing the findings to address type2 diabetes metabolic risk factors and nutrition related issues in Dar es Salaam and elsewhere. Reports will be produced and disseminated to the sponsors and the local health bodies and other stakeholders. At least three manuscripts manuscripts addressing different components in this project will be written and published in local and international peer reviewed journals.

# Reference

1. IDF. The IDF consensus worldwide definition of the metabolic syndrome. B-1000 Brussels, Belgium; 2006.

2. Panel NCEP Expert. Detection, Evaluation, and Treatment of High Blood Cholesterol in Adults (Adult Treatment Panel III) Final Report. Arch Intern Med [Internet]. 2002;151(6):284. Available from: http://archinte.jamanetwork.com/article.aspx?doi=10.1001/archinte.1991.00400060019005

3. WHO. Definition, Diagnosis and Classification of Diabetes Mellitus and its Complications. 1999.

4. Belin RJ, Allison M, Martin L, Shikany JM, Larson J, Tinker L, et al. Diet quality and the risk of cardiovascular disease : the Women ’ s Health. Am J Clin Nutr. 2011;94:49–57.

5. Akbaraly T, Ferrie J, Berr C, Brunner E, Head J, Marmot M, et al. Alternative Healthy Eating Index and mortality over 18 years of follow-up: results from the Whitehall II cohort. Am J Clin Nutr [Internet]. 2011;94(1):247–53. Available from: http://discovery.ucl.ac.uk/1313914/

6. WHO. Eseential Safety Requirements for Street-Vended Foods. World Heal Organ. 1996;96.7:36.

7. Bryan FL, Jermini M, Schmitt R, Chilufya EN, Michael M, Matoba A, et al. Harzards associated with holding and reheating foods at vending sites in a small town in Zambia. J Food Prot. 1997;60(4)(4):391–8.

8. WHO. The world health report 2002 - reducing risks, promoting healthy life. WEducation Heal (Abingdon, England). 2002;16(2):230.

9. American Dietary Guidelines. 2015 – 2020 Dietary Guidelines for Americans. 2015 – 2020 Dietary Guidelines for Americans (8th edition). 2015.

10. Hall V, Thomsen R, Henriksen O, Lohse N. Diabetes in Sub Saharan Africa 1999-2011: Epidemiology and public health implications. A systematic review. BMC Public Health [Internet]. 2011;11(1):564. Available from: http://www.biomedcentral.com/1471-2458/11/564

11. WHO. Global Report on Diabetes. Isbn [Internet]. 2016;978:88. Available from: http://www.who.int/about/licensing/%5Cnhttp://apps.who.int/iris/bitstream/10665/204871/1/9789241565257_eng.pdf

12. Ogurtsova K, da Rocha Fernandes JD, Huang Y, Linnenkamp U, Guariguata L, Cho NH, et al. IDF Diabetes Atlas: Global estimates for the prevalence of diabetes for 2015 and 2040. Diabetes Res Clin Pract [Internet]. 2017;128:40–50. Available from: http://dx.doi.org/10.1016/j.diabres.2017.03.024

13. McLarty D, Kitange H, Mtinangi B, Makene W. Prevalence of diabetes and impaired glucose tolerance in rural Tanzania. Lancet [Internet]. 1989;333(8643):871–5. Available from: http://www.sciencedirect.com/science/article/pii/S0140673689928663

14. WHO. STEPs Survey Report of Risk factors of Non communicable diseases. 2013.

15. Seuring T, Archangelidi O, Suhrcke M. The Economic Costs of Type 2 Diabetes: A Global Systematic Review. Pharmacoeconomics [Internet]. 2015;33(8):811–31. Available from: http://dx.doi.org/10.1007/s40273-015-0268-9

16. Liu YM, Chen SLS, Yen AMF, Chen HH. Individual risk prediction model for incident cardiovascular disease: A Bayesian clinical reasoning approach. Int J Cardiol [Internet]. 2013;167(5):2008–12. Available from: http://dx.doi.org/10.1016/j.ijcard.2012.05.016

17. Roman-urrestarazu A, Mohamed F, Ali H, Reka H, Renwick MJ, Roman GD, et al. Structural equation model for estimating risk factors in type 2 diabetes mellitus in a Middle Eastern setting : evidence from the STEPS Qatar. 2016;

18. Kagaruki GB, Mayige MT, Ngadaya ES, Kimaro GD, Kalinga AK, Kilale AM, et al. Magnitude and risk factors of non-communicable diseases among people living with HIV in Tanzania: A cross sectional study from Mbeya and Dar es Salaam regions. BMC Public Health. 2014;14(1):1–9.

19. Ruhembe CC. Prevalence, Awareness and Management of Type 2 Diabetes Mellitus in Mwanza City, Tanzania. 2016;16(2):1–11. Available from: http://www.suaire.suanet.ac.tz:8080/xmlui/bitstream/handle/123456789/1469/CAROLYNE CHARLES RUHEMBE.pdf?sequence=1&isAllowed=y

20. Stanifer JW, Cleland CR, Makuka GJ, Egger JR, Maro V, Maro H, et al. Prevalence, risk factors, and complications of diabetes in the Kilimanjaro region: A population-based study from Tanzania. PLoS One. 2016;11(10):1–13.

21. Msambichaka B, Eze IC, Abdul R, Abdulla S, Klatser P, Tanner M, et al. Insufficient fruit and vegetable intake in a low- and middle-income setting: A population-based survey in semi-Urban Tanzania. Nutrients. 2018;10(2).

22. Ibrahim N, Moy FM, Awalludin IAN, Ali ZM, Ismail IS. Effects of a community-based healthy lifestyle intervention program (Co-HELP) among adults with prediabetes in a developing country: A quasi-experimental study. PLoS One [Internet]. 2016;11(12):1–21. Available from: http://dx.doi.org/10.1371/journal.pone.0167123

23. Larsson SC, Virtamo J, Wolk A. Total and specific fruit and vegetable consumption and risk of stroke: A prospective study. Atherosclerosis [Internet]. 2013;227(1):147–52. Available from: http://dx.doi.org/10.1016/j.atherosclerosis.2012.12.022

24. WHO. Global status report on noncommunicable diseases 2014. World Health. 2014;176.

25. Mayige M, Kagaruki G, Ramaiya K, Swai A. Non communicable diseases in Tanzania: a call for urgent action. Tanzan J Heal Res [Internet]. 2011;13(5 Suppl 1):378–86. Available from: http://pesquisa.bvsalud.org/portal/resource/pt/mdl-26591992

26. Kinabo J. DRAFT – NOT FOR QUOTATION A Case Study of Dar es Salaam City , Tanzania Joyce KINABO Paper prepared for the FAO technical workshop on Information on the Authors : 2003.

27. FAO. Improving the nutritional quality of street foods to better meet the micronutrient needs of urban populations. 2007;

28. Fellows P, Hilmi M. Selling street and snack foods. FAO Diversification Booklet 18. 2011. 1-9 p.

29. Musvanhiri P. Zimbabwe:-street food vendors banned over typhoid, cholera fears. Available from: http://www.dw.com/en/zimbabwe-street-food-vendors-banned-over-typhoid-cholera-fears/a-37104481. 2017.

30. Pokhrel P, Sharma D. A study on assessment of food safety knowledge and practices among the street food vendor of urban and semi urban areas of Guwahati , Assam. Int J Home Sci. 2016;2(2):85–9.

31. Batchelder AR. Street food quality A matter of neatness and trust A qualitative study of local practices and perceptions of food quality, food hygiene and food safety in urban Kumasi, Ghana. [Internet]. Vol. 10, Journal of Environment Quality. 2006. Available from: https://cgspace.cgiar.org/bitstream/handle/10568/17110/17110.pdf?sequence=1%0Ahttps://www.agronomy.org/publications/jeq/abstracts/10/4/JEQ0100040515

32. Lopes Nonato I, Oliveira De Almeida Minussi L, Pascoal GB, De-Souza DA, Br D. Nutritional Issues Concerning Street Foods. J Clin Nutr Diet J Clin Nutr Diet [Internet]. 2016;2(1):1–9. Available from: http://clinical-nutrition.imedpub.com/archive.php%0Ahttp://www.imedpub.com%0Ahttp://dx.doi.org/10.4172/2472-1921.100014

33. Lyana A, Manimbulu N. Culture and Food Habits in Tanzania and Democratic Republic of Congo. J Hum Ecol J Hum Ecol [Internet]. 2014;48(1):9–21. Available from: http://www.krepublishers.com/02-Journals/JHE/JHE-48-0-000-14-Web/JHE-48-1-000-14-Abst-PDF/JHE-48-1-009-14-2666-Lyana-A-Z/JHE-48-1-009-14-2666-Lyana-A-Z-T[2].pdf

34. Chege PM, Kimiywe JO, Ndungu ZW. Influence of culture on dietary practices of children under five years among Maasai pastoralists in Kajiado , Kenya. Int J Behav Nutr Phys Act [Internet]. 2015;1–6. Available from: http://dx.doi.org/10.1186/s12966-015-0284-3

35. Gittelsohn J, Vastine AE. Animal Source Foods to Improve Micronutrient Nutrition and Human Function in Developing Countries Sociocultural and Household Factors Impacting on the Selection , Allocation and Consumption of Animal Source Foods : Current Knowledge and Application 1. 2003;4036–41.

36. Mbwana HA, Kinabo J, Lambert C, Biesalski HK. Determinants of household dietary practices in rural Tanzania : Implications for nutrition interventions. Cogent Food Agric [Internet]. 2016;13:1–13. Available from: http://dx.doi.org/10.1080/23311932.2016.1224046

37. Willett WC. Balancing life-style and genomics research for disease prevention. Science (80- ). 2002;296(5568):695–8.

38. Harvard T. H. Chan School of Public Health. Healthy Eating Plate & Healthy Eating Pyramid. Harvard Sch Public Heal [Internet]. 2011;1–6. Available from: https://www.hsph.harvard.edu/nutritionsource/healthy-eating-plate/

39. Steyn NP, McHiza Z, Hill J, Davids YD, Venter I, Hinrichsen E, et al. Nutritional contribution of street foods to the diet of people in developing countries: A systematic review. Public Health Nutr. 2014;17(6):1363–74.

40. Cluss PA, Ewing L, King WC, Reis EC, Dodd JL, Penner B. Nutrition knowledge of low-income parents of obese children. Transl Behav Med. 2013;3(2):218–25.

41. URT. 2012 Population and Housing Census Population Distribution by Administrative areas. NBS Minist Financ. 2013;177,180.

42. United Nations. The World’s Cities in 2016: Data Booklet. Econ Soc Aff [Internet]. 2016;29. Available from: http://www.un.org/en/development/desa/population/publications/pdf/urbanization/the_worlds_cities_in_2016_data_booklet.pdf

43. Kagaruki MTM, Esther S. Ngadaya, Andrew M.Kilale, Amos Kahwa, Amani F. Shao GD, Kimaro, Chacha M. Manga, Doris Mbata, Godlisten S. Materu RMM, Mfinanga SG. Knowledge and perception on type2 diabetes and hypertension among HIV clients utilizing care and treatment services: A cross sectional study from Mbeya and Dar es Salaam regions in Tanzania. :1–19.

44. Lemeshow S, Hosmer Jr DW, Klar J, Lwanga SK. Part 1: Statistical Methods for Sample Size Determination. Adequacy Sample Size Heal Stud [Internet]. 1990;247. Available from: http://apps.who.int/iris/bitstream/10665/41607/1/0471925179_eng.pdf?ua=1

45. Shah V, Kamdar P, Shah N. Assessing the knowledge, attitudes and practice of type 2 diabetes among patients of Saurashtra region, Gujarat. Int J Diabetes Dev Ctries [Internet]. 2009;29(3):118. Available from: http://www.ijddc.com/text.asp?2009/29/3/118/54288

46. Fatema K, Hossain S, Natasha K, Chowdhury HA, Akter J, Khan T, et al. Knowledge attitude and practice regarding diabetes mellitus among Nondiabetic and diabetic study participants in Bangladesh. BMC Public Health. 2017;17(1):1–10.

47. Mumu SJ, Saleh F, Ara F, Haque MR, Ali L. Awareness regarding risk factors of type 2 diabetes among individuals attending a tertiary-care hospital in Bangladesh: A cross-sectional study. BMC Res Notes. 2014;7(1).

48. Barzegari A, Ebrahimi M, Azizi M, Ranjbar K. A Study of Nutrition Knowledge , Attitudes and Food Habits of College Students. A Study Nutr Knowl , Attitudes Food Habits Coll Students [Internet]. 2011;15(7):1012–7. Available from: https://pdfs.semanticscholar.org/f034/7ca56ec6ec6fefc1b8e84e6f5c828c86bac7.pdf

49. Yang H, Gao J, Ren L, Li S, Chen Z, Huang J, et al. Association between Knowledge-Attitude-Practices and Control of Blood Glucose, Blood Pressure, and Blood Lipids in Patients with Type 2 Diabetes in Shanghai, China: A Cross-Sectional Study. J Diabetes Res. 2017;2017.

50. Parmenter K, Wardle J. Development of a general nutrition knowledge questionnaire for adults. Eur J Clin Nutr [Internet]. 1999;53(4):298–308. Available from: http://www.nature.com/doifinder/10.1038/sj.ejcn.1600726

51. Bano R, AlShammari E, Fatima SB, Norah Ayed A-S. A comparative study of Knowledge , Attitude , Practice of nutrition and non-nutrition student towards a balanced diet in. J Nurs Heal Sci [Internet]. 2013;2(3):29–36. Available from: www.iosrjournals.org

52. Mashili FL, Kagaruki GB, Mbatia J, Nanai A, Saguti G, Maongezi S, et al. Physical Activity and Associated Socioeconomic Determinants in Rural and Urban Tanzania : Results from the 2012 WHO-STEPS Survey. 2018;2018:7–10.

53. WHO. Physical status: the use and interpretation of anthropometry. Report of a WHO Expert Committee. Vol. 854, World Health Organization technical report series. 1995. p. 1–452.

54. Ma WY, Yang CY, Shih SR, Hsieh HJ, Hung CS, Chiu FC, et al. Measurement of waist circumference: Midabdominal or iliac crest? Diabetes Care. 2013;36(6):1660–6.

55. WHO. Waist Circumference and Waist-Hip Ratio: Report of a WHO Expert Consultation. World Heal Organ. 2008;(December):8–11.

56. Ahmad S, Ahmad T. Assessment of knowledge, attitude and practice among diabetic patients attending a health care facility in North India. Indian J Basic Appl Med Res. 2015;4(3):501–9.

57. Naug H, Colson N, Kundur A, Santha Kumar A, Tucakovic L, Roberts M, et al. Occupational health and metabolic risk factors: A pilot intervention for transport workers. Int J Occup Med Environ Health [Internet]. 2016;29(4):573–84. Available from: http://www.journalssystem.com/ijomeh/Occupational-health-and-metabolic-risk-factors-A-pilot-intervention-for-transport-workers,59127,0,2.html

58. Jadad A, Enkin M. Bias in randomized controlled trials. Randomized Control Trials Quest Answers, Musings, Second Ed [Internet]. 2008;29–47. Available from: http://onlinelibrary.wiley.com/doi/10.1002/9780470691922.ch3/summary

59. Gluud LL. Bias in clinical intervention research. Am J Epidemiol. 2006;163(6):493–501.

60. Jager KJ, Zoccali C, MacLeod A, Dekker FW. Confounding: What it is and how to deal with it. Kidney Int [Internet]. 2008;73(3):256–60. Available from: http://dx.doi.org/10.1038/sj.ki.5002650

61. McNamee R. Regression modelling and other methods to control confounding. Occup Environ Med. 2005;62(7):500–6.

62. Viera AJ, Bangdiwala SI. Eliminating bias in randomized controlled trials: Importance of allocation concealment and masking. Fam Med. 2007;39(2):132–7.

Appendix A: <https://vizhub.healthdata.org/gbd-compare/>


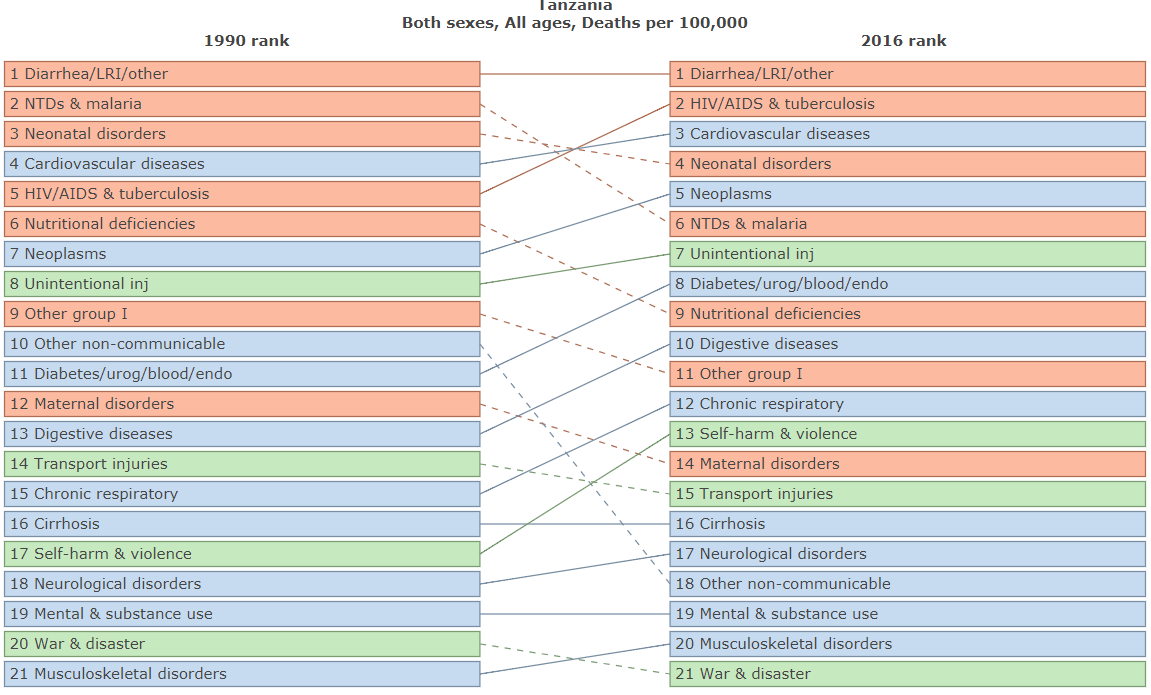


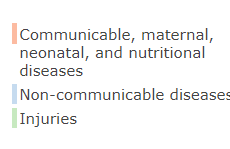


Appendix B: **Data Collection tools**

**Effectiveness of prevention package and model for predicting metabolic risk factors of type2 diabetes among street cooked food consumers in Tanzania: A cluster randomized control trial**

**Section A: Information for Identification**

| **Q-Number** | **Question** | **Response** | **Code** |
| --- | --- | --- | --- |
| 1. | District: 1. Ilala 2. Kinondoni 3. Ubungo |  | Qn1 |
| 2. | Market place: |  | Qn2 |
| 3. | Category of cluster (market place): 1. Intervention 2. Control |  | Qn3 |
| 4. | Interviewer Names (Initials): |  | Qn4 |
| 5. | Date of an interview |  | Qn5 |
| 6. | Consent has been read and obtained: 1. Yes 2. No |  | Qn6 |
| 7. | Time of starting interview (24 hour clock) |  | Qn7 |
| 8. | Time of completing interview (24 hour clock) |  | Qn8 |
| 9. | Family Surname |  | Qn9 |
| 10. | First Name |  | Qn10 |
| 11. | Contact phone number where possible |  | Qn11 |
| 12. | Owner of the phone: 1. Office phone 2. Home  3. Neighbours’ 4. Others___________ |  | Qn12 |

**Section B: Step 1: Demographic Information**

| **Q-Number** | **Question** | **Response** | **Code** |
| --- | --- | --- | --- |
| 13. | Sex: 1. Male 2. Female |  | Qn13 |
| 14. | What is your date of birth? DD/MM/YYYY______________________  *Don't Know 77 77 7777* |  | Qn14 |
| 15. | How old are you? |  | Qn15 |
| 16. | In total, how many years have you spent at school or in full-time study (excluding pre-school)? ___________________________________ |  | Qn16 |
| 17. | Highest level of education attained: 1. None 2. Primary  3. Secondary 4. College |  | Qn17 |
| 18. | What is your **marital status**? 1. Never married 2. Married  3. Separated 4. Divorced  5. Widowed 6. Cohabiting |  | Qn18 |
| 19. | Which of the following best describes your **main work** status over the past 12 months?  1. Government employee 2. Non-government employee  3. Self-employed 4.Non Paid  5. Homemaker 6. Retired  7. Unemployed (able to work) 8. Unemployed (unable to work)  9. Refused |  | Qn19 |
| 20. | How many people older than 18 years, including yourself, live in your household?________________ |  | Qn20 |
| 21. | Taking **the past year**, can you tell me what the average earnings of the household have been?   1. Per day______________________________________ 2. Per week______________________________________ 3. Per month_____________________________________ 4. Per year_______________________________________ |  | Qn21a  Qn21b  Qn21c  Qn21d |

**Section D: Step 1: Attitudes towards living healthy life to prevent type2 diabetes**

| **Q-Number** | **Question** | **Response** | **Code** |
| --- | --- | --- | --- |
|  | **Statements** | 1.Strogly agree  2.Agree  3.Disagree  4.Strongly disagree |  |
| 22. | It is possible to prevent diabetes by dietary management |  | Qn22 |
| 23. | Family history of diabetes influences to follow disciplined life |  | Qn23 |
| 24. | Obesity increases the risk of diabetes |  | Qn24 |
| 25. | Regular exercise needs lots of effort |  | Qn25 |
| 26. | Everybody should be aware regarding prevention of diabetes with increasing age |  | Qn26 |
| 27. | If there is increased chance of getting diabetes e.g. having family history then there is nothing to do to prevent or delay occurrences of the disease. |  | Qn27 |
| 28. | If you try you can reduce the risk of diabetes |  | Qn28 |
| 29. | People who take steps to reduce the risk factors of diabetes, their possibility of occurring diabetes is less |  | Qn29 |
| 30. | You have little control on your health risk |  | Qn30 |
| 31. | If a person is rich and fat if the suddenly becomes thin and slender is a sign of running bankrupt |  | Qn31 |
| 32. | If somebody is fat has to maintain his/her body structure otherwise can be considered to be HIV positive |  | Qn32 |
| 33. | To become thin and slender is not good because people will consider one to be HIV positive |  | Qn33 |
| 34. | A glass of unsweeted juice is better than chewing the actual fruits |  | Qn34 |
| 35. | It is very difficult to yourself to avoind overfeeding if delicious foods are prently available |  | Qn35 |
| 36. | If salt is low teste low in foods it is better to add when you are eating in order to maintan the food teste |  | Qn36 |

**Section D: Step 1: Knowledge on type2 diabetes**

**Note: *This is a survey, not a test. Your answers will help identify which dietary advice people finding confusing. Therefore, if you do not know the answer, say `not sure/DK' rather than guess.***

| **Q-Number** | | **Question** | **Response** | **Code** |
| --- | --- | --- | --- | --- |
| *T2DM: Risk factors, symptoms, preventions strategies, complications Specific dietary related prevention strategies, nutritional values of foods and disease related with poor eating habits* | | | | |
| 37. | Have you had about diabetes? 1. Yes 2. No | |  | Qn37 |
| 38. | **If yes; mention at least five the risk factors of diabetes** | |  |  |
|  | 1. Age (40+ years) | |  | Qn38a |
|  | 1. Physical inactivity | |  | Qn38b |
|  | 1. Raised blood glucose | |  | Qn38c |
|  | 1. Raised cholesterol | |  | Qn38d |
|  | 1. Unhealthy eating | |  | Qn38e |
|  | 1. Family history of diabetes | |  | Qn38f |
|  | 1. Raised blood pressure | |  | Qn38g |
|  | 1. Alcohol consumption | |  | Qn38h |
|  | 1. Smoking | |  | Qn38i |
|  | 1. Excessive body weight | |  | Qn38j |
|  | 1. Gestational diabetes | |  | Qn38k |
|  | 1. Depression/stress | |  | Qn38l |
|  | 1. Others (specify)_______________________________ | |  | Qn38m |
| 39. | **Mention at least five symptoms of diabetes** | |  |  |
|  | 1. Extreme hunger | |  | Qn39a |
|  | 1. Frequent thirst | |  | Qn39b |
|  | 1. Frequent urination | |  | Qn39c |
|  | 1. Extreme weight loss | |  | Qn39d |
|  | 1. Blurred vision | |  | Qn39e |
|  | 1. Fatigue | |  | Qn39f |
|  | 1. Dry mouth | |  | Qn39g |
|  | 1. Dry or itchy skin | |  | Qn39h |
|  | 1. Poor wound healing | |  | Qn39i |
|  | 1. Extreme hunger | |  | Qn39j |
|  | 1. Frequent thirst | |  | Qn39k |
|  | 1. Others (specify)_______________________ | |  | Qn39l |
| 40. | **Mention at least five strategies for preventing type two diabetes** | | |  |
|  | 1. Control excessive body weight | |  | Qn40a |
|  | 1. Healthy eating habit | |  | Qn40b |
|  | 1. Control blood pressure | |  | Qn40c |
|  | 1. Healthy eating habit | |  | Qn40d |
|  | 1. Being physical active | |  | Qn40e |
|  | 1. Control blood glucose | |  | Qn40f |
|  | 1. Control blood cholesterol | |  | Qn40g |
|  | 1. Avoid excessive alcohol drinking | |  | Qn40h |
|  | 1. Avoid stress/depression | |  | Qn40i |
|  | 1. Quit smoking | |  | Qn40j |
|  | 1. Treat raised blood glucose during pregnancy | |  | Qn40k |
|  | 1. Others (specify)___________________________ | |  | Qn40l |
| 41. | **Mention at least five complications of type2 diabetes** | |  |  |
|  | 1. Amputation | |  | Qn41a |
|  | 1. Stroke | |  | Qn41b |
|  | 1. Kidney failure | |  | Qn41c |
|  | 1. Blindness | |  | Qn41d |
|  | 1. Impotence | |  | Qn41e |
|  | 1. Nerve damage | |  | Qn41f |
|  | 1. Heart attacks | |  | Qn41g |
|  | 1. Others (specify)_____________________ | |  | Qn41h |
| **Nutritional values** | | | | |
| 42. | What is the best composition of portion of carbohydrate, fruits, vegetables and proteins healthy food plate?  1) ¼ Carbohydrate, ¼ Protein, ½ Fruits and vegetables, Water/milk  2) DK  3) Others_______________ | |  | Qn42 |
| 43. | Do you think health experts recommend that people should be eating more, the same amount, or less of these foods? (Tick one box per food) | |  |  |
|  | **Food** | | **1.More**  **2.Same**  **3.Less**  **4.Not sure** |  |
|  | a) Vegetables | |  | Qn43a |
|  | b) Sugary foods | |  | Qn43b |
|  | c) Meat | |  | Qn43c |
|  | d) Starchy foods | |  | Qn43d |
|  | e) Fatty foods | |  | Qn43e |
|  | f) High fibre foods | |  | Qn43f |
|  | g) Fruit | |  | Qn43g |
|  | h) Salty foods | |  | Qn43h |
| 44. | How many servings of fruit and vegetables a day do you think experts are advising people to eat? (One serving could be, for example, an apple or a handful of chopped carrots)   1. Fruits _______________   b) Vegetable_______________ | |  | Qn44 |
| 45. | Saturated fats are mainly found in:(tick one)  1.Vegetable oils  2. Dairy products  3. Both (a) and  4. Not sure | |  | Qn45 |
| 46. | If a person wanted to reduce the amount of salt in their diet, which would be the best choice? (tick one)  1.Buy food with already added salts like sausages, manufacture beefs, chips crips etc  2. Add salt during cooking himself/herelf  3. Not sure | |  | Qn46 |
| 47. | Are you aware of the major non-communicable disease which is related to how much salt or sodium people eat?   1. Yes 2. No 3. Not sure | |  | Qn47 |
| 48 | If yes, which non-communicable disease do you think is related to salt?   1. High blood pressure 2. Not remember 3. Other______________________ | |  | Qn48 |
| 49. | Are you aware of any major non-communicable diseases that are related to a low intake of fruit and vegetables?   1. Yes 2. No 3. Not sure | |  | Qn49 |
| 50 | If yes, what non-communicable diseases do you think are related to a low intake of fruit and vegetables?   1. High blood pressure 2. Diabetes 3. Cancer 4. Not remember 5. Others________________ | |  | Qn50a  Qn50b  Qn50c  Qn50d  Qn50e |
| 51. | Are you aware of any major non-communicable diseases that are related to the amount of fat people eat? (Yes/No/DK) | |  | Qn51 |
| 52. | If yes, what non-communicable do you think are related to fat?   1. High blood pressure 2. Diabetes 3. Cancer 4. Not remember 5. Others________________ | |  | Qn52a  Qn52b  Qn52c  Qn52d  Qn52e |
| 53. | Are you aware of any major non-communicable diseases that are related to a low intake of fibre?   1. Yes 2. No 3. Not sure | |  | Qn53 |
| 54. | If yes, what diseases or health problems do you think are related to low intake of fibre?   1. High blood pressure 2. Diabetes 3. Cancer 4. Not remember 5. Others________________ | |  | Qn54a  Qn54b  Qn54c  Qn54d  Qn54e |
| 55. | Are you aware major non-communicable diseases that are related to excessive drinking of alcohol?   1. Yes 2. No 3. Not sure | |  | Qn55 |
| 56. | If yes, what non-communicable diseases that are related to excessive drinking of alcohol?   1. High blood pressure 2. Diabetes 3. Cancer 4. Not remember 5. Others________________ | |  | Qn56 |

**Section E1: Step 1: Behavioural Measurements –Tobacco Use**

***Now I am going to ask you some questions about various health behaviours. This includes things like smoking, drinking alcohol, eating fruits and vegetables and physical activity. Let's start with tobacco.***

| **Q-Number** | **Question** | **Response** | **Code** |
| --- | --- | --- | --- |
| 57. | Do you currently smoke any **tobacco products**, such as cigarettes, cigars or pipes? 1. Yes 2. No |  | Qn57 |
| 58. | Do you currently smoke tobacco products **daily**?  1. Yes 2. No |  | Qn58 |
| 59. | How old were you when you **first started** smoking daily?  Age (years)______________________DK: 77 |  | Qn59 |
| 60. | Do you remember how long ago it was? (Record one option only)   1. In Years_______________________ 2. OR In Months______________ 3. OR In Weeks_____________ 4. Don’t remember____________ |  | Qn60a  Qn60b  Qn60c  Qn60d |
| 61. | On average, **how many (number of sticks)** of the following do you smoke each day?*(RECORD FOR EACH TYPE, USE SHOWCARD)* *Don’t Know* |  |  |
|  | 1. Manufactured cigarettes__________ |  | Qn61a |
|  | 1. Hand-rolled cigarettes__________ |  | Qn61b |
|  | 1. Pipes full of tobacco__________ |  | Qn61c |
|  | 1. Cigars, cheroots, cigarillos__________ |  | Qn61d |
|  | 1. Others______________________________ |  | Qn61e |
| 62. | In the past, did you ever smoke daily?  1. Yes 2. No |  | Qn62 |
| 63. | How old were you when you stopped smoking daily? |  | Qn63 |
| 64. | How long ago did you stop smoking daily? (RECORD ONLY 1, NOT ALL 3)   1. In Years_______________________ 2. OR In Months______________ 3. OR In Weeks_____________ 4. Don’t remember____________ |  | Qn64a  Qn64b  Qn64c  Qn64d |
| 65. | Do you **currently use smokeless tobacco** products **daily?**  1. Yes 2. No |  | Qn65 |
| 66. | During the past 7 days, on how many days did someone **in your home** smoke when you were present? |  | Qn66 |
| 67. | During the past 7 days, on how many days did someone smoke in closed areas **in your workplace** (in the building, in a work area or a specific office) when you were present? |  | Qn67 |

**Section E2: Step 1: Behavioural Measurements –Alcohol Consumption**

***The next questions ask about the consumption of alcohol.***

| **Q-Number** | **Question** | **Response** | **Code** |
| --- | --- | --- | --- |
| 68. | Have you **ever** consumed an alcoholic drink such as beer, wine, spirits, fermented cider or *[add other local examples]*?  *(USE SHOWCARD OR SHOW EXAMPLES)*   1. Yes 2. No |  | Qn68 |
| 69. | Have you consumed an alcoholic drink within the **past 12 months**?  Yes 2. No |  | Qn69 |
| 70. | During the past 12 months, **how frequently** have you had at least one alcoholic drink? *(READ RESPONSES, USE SHOWCARD)*  1 .Daily  2. 5-6 days per week  3. 1-4 days per week  4. 1-3 days per month  5. Less than once a month |  | Qn70 |
| 71. | Have you consumed an alcoholic drink within the **past 30 days**?  Yes 2. No |  | Qn71 |
| 72. | During the past 30 days, on how many **occasions** did you have at least one alcoholic drink?  Number _______________Don't know 77 |  | Qn72 |
| 73. | During the past 30 days, when you drank alcohol, **on average**, how many **standard alcoholic drinks** did you have during one drinking occasion?*(USE SHOWCARD)*  Number _______________Don't know 77 |  | Qn73 |
| 74. | During the past 30 days, what was the **largest number** of standard alcoholic drinks you had on a single occasion, counting all types of alcoholic drinks together? |  | Qn74 |
| 75. | During the past 30 days, how many times did you have for **men**: **five or more** for **women**: **four or more** standard alcoholic drinks in a single drinking occasion? |  | Qn75 |

**Section E3: Step 1: Behavioural Measurements –Diet**

***The next questions ask about the fruits and vegetables that you usually eat. I have a nutrition card here that shows you some examples of local fruits and vegetables. Each picture represents the size of a serving. As you answer these questions please think of a typical week in the last year.***

| **Q-Number** | **Question** | **Response** | **Code** |
| --- | --- | --- | --- |
| 76. | In a typical week, on how many days do you **eat fruit**? *If Zero days, skip*  Number of days____________________Don't Know 77 |  | Qn76 |
| 77. | How many **servings** of fruit do you eat on **one** of those days? (*USE SHOWCARD) If Zero days, skip*  Number of days___________________Don't Know 77 |  | Qn77 |
| 78. | In a typical week, on how many days do you **eat vegetables**?*(USE SHOWCARD) If Zero days, skip*  Number of days___________________Don't Know 77 |  | Qn78 |
| 79. | How many **servings** of vegetables do you eat on one of those days? *(USE SHOWCARD) If Zero days, skip*  Number of days____________________Don't Know 77 |  | Qn79 |
| 80. | What type of **oil or fat is most often** used for meal preparation in your household?*(USE SHOWCARD)* *(SELECT ONLY ONE)*   1. Animal Oil 2. Vegetable oil 3. Both(mixed) 4. DK |  | Qn80 |
| 81. | On average, how many meals per week do you eat that were not prepared at a home? By meal, I mean breakfast, lunch and dinner.  Number of days____________________Don't Know 77 |  | Qn81 |
| 82. | Do you add salt when you eating some food?   1. Yes 2. No |  | Qn82 |

**Section E4: Step 1: Behavioural Measurements –Physical activities**

Next I am going to ask you about the time you spend doing different types of physical activity in a typical week. Please answer these questions even if you do not consider yourself to be a physically active person. Think first about the time you spend doing work. Think of work as the things that you have to do such as paid or unpaid work, study/training, household chores, harvesting food/crops, fishing or hunting for food, seeking employment*.* In answering the following questions 'vigorous-intensity activities' are activities that require hard physical effort and cause large increases in breathing or heart rate, 'moderate-intensity activities' are activities that require moderate physical effort and cause small increases in breathing or heart rate.

| **Q-Number** | **Question** | **Response** | **Code** |
| --- | --- | --- | --- |
| **Work Related Activities** | | | |
| 83. | Does your work involve vigorous-intensity activity that causes large increases in breathing or heart rate like lifting heavy loads, manual construction work, digging etc for at least 10 minutes continuously? *USE SHOWCARD)*   1. Yes 2. No |  | Qn83 |
| 84. | In a typical week, on how many days do you do vigorous-intensity activities as part of your work? Number of days________________________ |  | Qn84 |
| 85. | How much time do you spend doing vigorous-intensity activities at work on a typical day? | a) Hours : ___  b)Minutes:___ | Qn85a  Qn85b |
| 86. | Does your work involve moderate-intensity activity, that causes small increases in breathing or heart rate such as brisk walking*, carrying light loads, doing chores like cleaning, washing or ironing clothes etc ,*for at least 10 minutes continuously?   1. Yes 2. No |  | Qn86 |
| 87. | In a typical week, on how many days do you do moderate-intensity activities as part of your work?  Number of days________________________ |  | Qn87 |
| 88. | How much time do you spend doing moderate-intensity activities at work on a typical day? | a) Hours : ___  b)Minutes:___ | Qn88a  Qn88b |
| **Travel to and from place** | | | |
| The next questions exclude the physical activities at work that you have already mentioned.  Now I would like to ask you about the usual way you travel to and from places. For example to work, for shopping, to market, to place of worship. | | | |
| **Q-Number** | **Question** | **Response** | **Code** |
| 89. | Do you walk or use a bicycle *(pedal cycle)* for at least 10 minutes continuously to get to and from places?   1. Yes 2. No |  | Qn89 |
| 90. | In a typical week, on how many days do you walk or bicycle for at least 10 minutes continuously to get to and from places?  Number of days________________________ |  | Qn90 |
| 91. | How much time do you spend walking or bicycling for travel on a typical day? | a) Hours : ___  b)Minutes:___ | Qn91a  Qn91b |
| **Recreational activities** | | | |
| The next questions exclude the work and transport activities that you have already mentioned.  Now I would like to ask you about sports, fitness and recreational activities (leisure), *[Insert relevant terms]*. | | | |
| **Q-Number** | **Question** | **Response** | **Code** |
| 92. | Do you do any vigorous-intensity sports, fitness or recreational *(leisure)* activities that cause large increases in breathing or heart rate like *running, playing football etc,* for at least 10 minutes continuously?  *(USE SHOWCARD)*   1. Yes 2. No |  | Qn92 |
| 93. | In a typical week, on how many days do you do vigorous-intensity sports, fitness or recreational *(leisure)* activities?  Number of days________________________ |  | Qn93 |
| 94. | How much time do you spend doing vigorous-intensity sports, fitness or recreational activities on a typical day? | a) Hours : ___  b)Minutes:___ | Qn94a  Qn94b |
| 95. | Do you do any moderate-intensity sports, fitness or recreational *(leisure)* activities that cause a small increase in breathing or heart rate such as brisk walking*, cycling, swimming, dancing* etc for at least 10 minutes continuously?   1. Yes 2. No |  | Qn95 |
| 96. | In a typical week, on how many days do you do moderate-intensity sports, fitness or recreational *(leisure)* activities?  Number of days________________________ |  | Qn96 |
| 97. | How much time do you spend doing moderate-intensity sports, fitness or recreational *(leisure)* activities on a typical day? | a) Hours : ___  b)Minutes:___ | Qn97a  Qn97b |
| **Sedentary behaviour** | | | |
| The following question is about sitting or reclining at work, at home, getting to and from places, or with friends including time spent sitting at a desk, sitting with friends, travelling in car, bus, train, reading, playing cards or watching television, but do not include time spent sleeping.  *(USE SHOWCARD)* | | | |
| 98. | How much time do you usually spend sitting or reclining on a typical day? | a) Hours : ___  b)Minutes:___ | Qn98a  Qn98b |

**Section E6: Step 1: Familial –Hypertension**

| **Q-Number** | **Question** | **Response** | **Code** |
| --- | --- | --- | --- |
| 99. | Have you ever had your blood pressure measured by a doctor or other health worker? 1. Yes 2. No |  | Qn99 |
| 100. | Have you ever been told by a doctor or other health worker that you have raised blood pressure or hypertension? 1. Yes 2. No |  | Qn100 |
| 101. | Have you been told in the past 12 months? 1. Yes 2. No |  | Qn101 |

**Section E7: Step 1: Familial –Diabetes**

| **Q-Number** | **Question** | **Response** | **Code** |
| --- | --- | --- | --- |
| 102. | Have you ever had your blood sugar measured by a doctor or other health worker? 1. Yes 2. No |  | Qn102 |
| 103. | Have you ever been told by a doctor or other health worker that you have raised blood sugar or diabetes? 1. Yes 2. No |  | Qn103 |
| 104. | Have you been told in the past 12 months? 1. Yes 2. No |  | Qn104 |

**Section F: Step 2 Physical Measurements**

| **Q-Number** | **Question** | **Response** | **Code** |
| --- | --- | --- | --- |
| **Height and weight** | | | |
| 105. | Device IDs for height and weight | a)Weight:_____ | Qn105a |
|  |  | b) Height:______ | Qn105b |
| 106. | Height (cm) | ______.____ | Qn106 |
| 107. | Weight (kgs) | ______.____ | Qn107 |
| **Waist Circumference** | | | |
| 108. | Device ID for waist | ___________ | Qn108 |
| 109. | Waist circumference (cm) | ______.____ | Qn109 |
| **Blood Pressure** | | | |
| 110. | Device ID for blood pressure |  | Qn110 |
| 111. | Cuff size used: 1. Small 2. Medium 3. Larger |  | Qn111 |
| 112. | Reading 1 a) Systolic ( mmHg) | __________ | Qn112a |
|  | b)Diastolic (mmHg) | __________ | Qn112b |
| 113. | Reading 2 a) Systolic ( mmHg) | __________ | Qn113a |
|  | b)Diastolic (mmHg) | __________ | Qn113b |
| 114. | Reading 3 a) Systolic ( mmHg) | __________ | Qn114a |
|  | b)Diastolic (mmHg) | __________ | Qn114b |
| 115. | During the past two weeks, have you been treated for raised blood pressure with drugs (medication) prescribed by a doctor or other health worker?   1. Yes 2. No |  | Qn115 |
| **Hip Circumference** | | | |
| 116. | Hip circumference (cm) | __________ | Qn116 |

**Section G: Step 3 Biochemical Measurements**

| **Q-Number** | **Question** | **Response** | **Code** |
| --- | --- | --- | --- |
| **Blood Glucose** | | | |
| 117. | During the past 12 hours have you had anything to eat or drink, other than water? 1. Yes 2. No |  | Qn117 |
| 118. | Technician names (initials) | ___________ | Qn118 |
| 119. | Device ID | ___________ | Qn119 |
| 120. | Time of day blood specimen taken (24 hour clock) | Hours :  minutes | Qn120 |
| 121. | Fasting blood glucose: *(mmol/l)* |  | Qn121 |
| 122. | Today, have you taken insulin or other drugs (medication) that have been prescribed by a doctor or other health worker for raised blood glucose?  1. Yes 2. No |  | Qn122 |

| **Q-Number** | **Question** | **Response** | **Code** |
| --- | --- | --- | --- |
| **Blood Lipids** | | | |
| 123. | Device ID |  | Qn123 |
| 124. | Total cholesterol *(mmol/l)* |  | Qn124 |
| 125. | During the past two weeks, have you been treated for raised cholesterol with drugs (medication) prescribed by a doctor or other 1. Yes 2. No |  | Qn125 |
| 126. | Triglycerides  *(mmol/l)* |  | Qn126 |
| 127. | During the past two weeks, have you been treated for raised Triglycerides with drugs (medication) prescribed by a doctor or other 1. Yes 2. No |  | Qn127 |
| 128. | High Density Lipoprotein *(mmol/l)* |  | Qn128 |
| 129. | During the past two weeks, have you been treated for low High Density Lipoprotein s with drugs (medication) prescribed by a doctor or other 1. Yes 2. No |  | Qn129 |
| 130. | Low Density Lipoprotein *mmol/l)* |  | Qn130 |
| 131. | During the past two weeks, have you been treated for raised Low Density Lipoprotein with drugs (medication) prescribed by a doctor or other  1. Yes 2. No |  | Qn131 |

**End term evaluation: The entire above plus**

| **Q-Number** | **Question** | **Response** | **Code** |
| --- | --- | --- | --- |
| **Blood Lipids** | | | |
| 132. | What is the number of healthy plates consumed on average per week? | _______ | Qn132 |

**Socio-economic and cultural factors which may impend uptake and adherence to healthy plate**

| **Q-Number** | **Question** | **Response** | **Code** |
| --- | --- | --- | --- |
| **Blood Lipids** | | | |
| 133. | Based on your experience do you think are some financial issues which from not consuming healthy plate? |  | Qn133 |
| 134. | In this place do you think there some cultural factors which may hinder you from not consuming healthy plate? |  | Qn134 |
| 135. | If yes, what are those factors? |  | Qn135 |
| 136. | What do you recommend towards improving availability, affordability and accessibility of healthy plate from the street food vendors? |  |  |
|  | 1. Availability: |  | Qn136a |
|  | 1. Affordability: |  | Qn136b |
|  | 1. Accessibility: |  | Qn136c |

**Ufanisi wa kipengele cha kinga na mwongozo kwa ajili ya kubaini vimeng’enywa hatarishi vya aina ya pili ya ugonjwa wa kisukari miongoni mwa walaji wa vyakula vilivyopikwa vya mitaani nchini Tanzania: Jaribio la utafiti**

**Sehemu A: Taarifa za utambulisho**

| **Namba ya swali** | **Swali** | **Jibu** | **Code** |
| --- | --- | --- | --- |
| 1. | Wilaya: 1. Ilala 2. Kinondoni 3. Ubungo |  | Qn1 |
| 2. | Jina la soko: |  | Qn2 |
| 3. | Aina ya soko kwenye utafiti: 1. Jaribio 2. Siyo jaribio |  | Qn3 |
| 4. | Majina ya mhojaji (vifupi):_______________ |  | Qn4 |
| 5. | Tarehe ya mahojiano______________________ |  | Qn5 |
| 6. | Fomu ya ridhaa imesomwa na ridhaa imepatikana?   1. Ndiyo 2. Hapana |  | Qn6 |
| 7. | Muda wa kuanza mahojiano (kwa masaa 24)_______________ |  | Qn7 |
| 8. | Muda wa kumaliza mahojiano (kwa masaa 24)______________ |  | Qn8 |
| 9. | Jina la ukoo______________________________ |  | Qn9 |
| 10. | Jina la kwanza____________________________ |  | Qn10 |
| 11. | Namba ya simu:___________________________ |  | Qn11 |
| 12. | Mmiliki wa simu: 1. Ya ofisini 2. Nyumbani  3. Jirani 4. Nyingine___________ |  | Qn12 |

**Sehemu B: Step 1: Taarifa za kidemographia**

| **Namba ya swali** | **Swali** | **Jibu** | **Code** |
| --- | --- | --- | --- |
| 13. | Jinsi: 1. Me 2. Ke |  | Qn13 |
| 14. | Tarehe ya kuzaliwa? DD/MM/YYYY______________________  *Sijui 77 77 7777* |  | Qn14 |
| 15. | Una umri gani? |  | Qn15 |
| 16. | Kwa jumla, umetumia miaka mingapi shuleni au katika masomo yako mpaka sasa (ukiondoa muda wa kabla ya shule ya msingi)?  ____________________________ |  | Qn16 |
| 17. | Elimu yako ya juu uliyofikia : 1. Hana 2. Msingi  3. Sekondari 4. Chuo |  | Qn17 |
| 18. | Hali ya ndoa? 1. Mseja 2. Ameoa/olewa  3. Wametengana 4. Mtalaka  5. Mjane/mgane 6. Wanaishi pamoja |  | Qn18 |
| 19. | Ni chanzo chako kikuu cha kipato kwa kipindi cha miezi 12 iliyopita?  1. Mwajiriwa wa serikali 2. Mwajiriwa siyo wa serikali  3. Ajira binafsi 4.Ajira bila malipo  5. Kazi za nyumbani 6. Mstaafu  7. Hana ajira (aweza kufanya kazi)  8. Hana ajira (hawezi kufanya kazi)  9. Kakataa kujibu |  | Qn19 |
| 20. | Katika kaya yako kuna watu wangapi wenye umri wa kuanzia miaka 18 na kuendelewa ikiwepo na wewe?  ________________ |  | Qn20 |
| 21. | Kwa mwaka jana, waweza niambia kwa wastani kaya yako ilikuwa inaingiza kipato kiasi gani kwa:   1. Siku______________________________________ 2. Wiki______________________________________ 3. Mwezi_____________________________________ 4. Mwaka_______________________________________ |  | Qn21a  Qn21b  Qn21c  Qn21d |

**Sehemu D: Step 1: Mtazamo kuhusu kuishi maisha ya afya ili kujikinga na ugonjwa wa kisukari**

| **Namba ya swali** | **Sentensi** | **Jibu** | **Code** |
| --- | --- | --- | --- |
|  |  | 1.Nakubali kabisa  2.Nakubali  3.Sikubali  4.Nakubali kabisa |  |
| 22. | Inawezekana kuzuia kisukari cha aina ya pili kwa ulaji unaozingatia afya |  | Qn22 |
| 23. | Historia ya familia ya ugonjwa wa kisukari ina mchango kwa mtu kuishi maisha ya taadhari |  | Qn23 |
| 24. | Kitambi kinaongeza hatari ya kupata ugonjwa wa kisukari |  | Qn24 |
| 25. | Kufanya mazoezi mara kwa mara kunaitaji juhudi nyingi |  | Qn25 |
| 26. | Kila mtu anapaswa kuwa mwangalifu kujikinga na kisukari kadiri umri wake unavyoongezeka |  | Qn26 |
| 27. | Kama kuna uwezekano mkubwa wa kupatatwa na kisukari mfano kuwa na historia ya kisukari ndani ya familia kunakuwa hakuna jinsi ya kufanya ili kujikinga au kuchelewesha kupata kisukari |  | Qn27 |
| 28. | Ukijaribu unaweza kupunguza uwezekano wa kupata Kisukari |  | Qn28 |
| 29. | Watu wanaochukua hatua wanaweza kupunguza vihatarishi vya kisukari, uwezekano wa kupata ugonjwa wa kisukari kwao ni mdogo |  | Qn29 |
| 30. | Wewe una juhudi kidogo za kupunguza vihatarishi vya kisukari |  | Qn30 |
| 31. | Kama mtu ni tajiri na ni mnene kisha akapungua uzito ghafla na kuwa mwembamba ni ishara kuwa amefilisika |  | Qn31 |
| 32. | Kama mtu ni mnene, inabidi ahahakishe hapungui uzito wake la sivyo jamii inaweza kumdhania kuwa ameathirika na UKIMWI |  | Qn32 |
| 33. | Kuwa mwembamba siyo vizuri kwani watu watakuona kuwa wewe ni muadhirika wa VVU. |  | Qn33 |
| 34. | Gilasi ya juisi ya matunda ambayo haijawekewa sukari ni bora kuliko kutafuna matunda |  | Qn34 |
| 35. | Ni vigumu sana kwako kujizuia kula ikiwa chakula kitamu kipo cha kutosha |  | Qn35 |
| 36. | Ikiwa chumvi ni kwenye chakula au mboga ni vyema kuongeza wakati wa kula ili kutopoteza utamu wa chakula |  | Qn36 |

**Sehemu D: Step 1: Uelewa kuhusu ugonjwa wa kisukari**

**Kumbuka**: Huuni utafiti, si mtihani. Majibu yako yatasaidia kutambua changamoto zilizopo katika jamii kuhusu ulaji bora. Kwa hiyo, kama hujui jibu, sema "sina na uhakika / Sijui" badala ya kubahatisha majibu.

| **Namba ya swali** | **Swali** | **Jibu** | **Code** |
| --- | --- | --- | --- |
| **Kisukari cha aina ya pili**: Vihatarishi, dalili, mikakati ya kuzuia, madhara ya kisukari, chakula bora, na magonjwa yasiyoambukiza yanayoambatana na ulaji usiozingatia afya. | | | |
| 37. | Umeishasikia kuhusu ugonjwa wa kisukari   1. Ndiyo 2.Hapana |  | Qn37 |
| 38. | **Kama jibu ni ndiyo, taja anagalau vihatarishi vitano vya ugonjwa wa kisukari.** |  |  |
|  | 1. Umri (Miaka 40 na kuendelea) |  | Qn38a |
|  | 1. Kutoshughulisha mwilia |  | Qn38b |
|  | 1. Kuongezeka sukari ndani ya damu |  | Qn38c |
|  | 1. Kuongezeka kwa mafuta kwenye damu |  | Qn38d |
|  | 1. Ulaji usiozingatia afya |  | Qn38e |
|  | 1. Historia ya kisukari ndani ya familia |  | Qn38f |
|  | 1. Shinikizo la damu la juu |  | Qn38g |
|  | 1. Unywaji wa pombe |  | Qn38h |
|  | 1. Uvutaji wa tumbaku |  | Qn38i |
|  | 1. Uzito wa juu |  | Qn38j |
|  | 1. Kuwa na kisukari wakati wa ujauzito |  | Qn38k |
|  | 1. Msongo wa mawazo |  | Qn38l |
|  | 1. Nyingine (taja)_______________________________ |  | Qn38m |
| 39. | **Taja angalau dalili 5 za kisukari** |  |  |
|  | 1. Njaa ya mara kwa mara |  | Qn39a |
|  | 1. Kiu ya mara kwa mara |  | Qn39b |
|  | 1. Kukojoa mara kwa mara hasa usiku |  | Qn39c |
|  | 1. Kupungua uzito ghafla |  | Qn39d |
|  | 1. Kutoona vizuri (upofu) |  | Qn39e |
|  | 1. Uchovu |  | Qn39f |
|  | 1. Midomo mikavu |  | Qn39g |
|  | 1. Ngozi kukauka na kuwasha |  | Qn39h |
|  | 1. Vidonda visivyopona haraka |  | Qn39i |
|  | 1. Others (specify)_______________________ |  | Qn39j |
| 40. | **Taja njia/mikakati 5 ya kuzuia ugongwa wa kisukari cha aina ya pili** | |  |
|  | 1. Kudhibiti uzito wa mwili |  | Qn39l |
|  | 1. Ulaji unaozingatia afya |  |  |
|  | 1. Kudhibiti shinikizo la damu |  | Qn40a |
|  | 1. Ulaji unaozingatia afya |  | Qn40b |
|  | 1. Kushughulisha mwili |  | Qn40c |
|  | 1. Kudhibiti kiwango cha sukari kwenye damu |  | Qn40d |
|  | 1. Kudhibiti kiwango cha mafuta kwenye damu |  | Qn40e |
|  | 1. Unywaji pombe unaozingatia afya |  | Qn40f |
|  | 1. Kuepuka msongo wa mawazo |  | Qn40g |
|  | 1. Kuacha kuvuta sigara |  | Qn40h |
|  | 1. Kutibu kiwango cha sukari wakati wa ujauzito |  | Qn40i |
|  | 1. Nyingine (taja)___________________________ |  | Qn40j |
| 41. | **Mention at least five complications of type2 diabetes** |  | Qn40k |
|  | 1. Kukatwa viuongo(miguu, mikono, vidole nk) |  | Qn40l |
|  | 1. Kiharusi (Stroke) |  |  |
|  | 1. Figo kushindwa kufanya kazi |  | Qn41a |
|  | 1. Upofu |  | Qn41b |
|  | 1. Kupungukiwa nguvu za kiume |  | Qn41c |
|  | 1. Uharibifu wa neva |  | Qn41d |
|  | 1. Shambulio la moyo |  | Qn41e |
|  | 1. Nyingine (taja)_____________________ |  | Qn41f |
| **Ubora wa chakula** | | | |
| 42. | Je! ni kiasi gani cha wanga, matunda, mboga mboga na protini kinachopaswa kuwa kwenye sahani ya chakula wakati wa ulaji kwa mtu anayezingatia afya?  1) ¼ ya wanga, ¼ ya protin, ½ matunda na mbogamboga, maji/maziwa  2)Sijui  3)Nyingine (taja)__________________ |  | Qn42 |
| 43. | Je! Unafikiri wataalamu wa afya wanashauri kwamba watu wanapaswa kula zaidi, kiasi sawa, au chini kwa vyakula vifuatavyo? (Tiki sanduku moja kwa kila chakula) |  |  |
|  | **Chakula** | **1.Zaidi**  **2.Kiasi saw**  **3.Kidogo**  **4.Sina hakika** |  |
|  | a) Mboga mboga |  | Qn43a |
|  | b) Vyakula vyenye sukari |  | Qn43b |
|  | c) Nyama nyekundu |  | Qn43c |
|  | d) Chakula cha wanga |  | Qn43d |
|  | e) Vyakula vyenye mafuta |  | Qn43e |
|  | f) Vyakula vyenye nyuzi |  | Qn43f |
|  | g) Matunda |  | Qn43g |
|  | h) Vyakula vyenye chumvi |  | Qn43h |
| 44. | Ni vipimo kiasi gani vya matunda na mboga mboga unafikiri vinashauriwa kuliwa na mtu kwa siku moja) (kipimo kimoja kinaweza kuwa chungwa moja au kiganja kimoja kilichojaa karoti zilizomenywa na kukatwakatwa)   1. Mtunda _______________ 2. Mbogamboga_______________ |  | Qn44 |
| 45. | Mafuta yaliyojaa sana hupatikana kwenye:  1.Mafuta ya mimea  2. Mafuta ya wanyama  3. Yote hapo juu  4. Sina uhakika |  | Qn45 |
| 46. | Ikiwa mtu antaka kupunguza kiasi cha chumvi katika chakula, ni nini chaguo bora kati ya haya yafuatayo? (chagua moja)  1. Kununua chakula ambacho tayari kimewekwa chunvi chumvi mfano sosegi, nyama za kopo, chipsi (clips) nk  2. Kuongeza chumvi mwenyewe wakati wa kupika  3. Sina uhakika |  | Qn46 |
| 47. | Je! Unafahamu magonjwa makubwa yasiyo ya kuambukizwa ambayo yanachochewa na kiasi kikubwa cha chumvi watu wanachokula?   1. Ndiyo 2. Hapana 3. Sina hakika |  | Qn47 |
| 48 | Ikiwa ndio, ni magonjwa gani yasiyotambulika unafikiri yanahusiana na chumvi?   1. Shinikizo la damu 2. Sijui 3. Nyingine (taja)______________________ |  | Qn48 |
| 49. | Je, unafahamu magonjwa yoyote yasiyo ya kuambukizwa yanayohusiana na ulaji mdogo wa matunda na mboga?   1. Ndiyo 2. Hapana 3. Sina hakika |  | Qn49 |
| 50 | Ikiwa ndio, ni magonjwa gani yasiyoyakuambukiza unafikiri yanahusiana na ulaji mdogo wa matunda na mboga?   1. Shinikizo la damu la juu 2. Kisukari 3. Kansa 4. Sijui 5. Nyingine (taja)______________________ |  | Qn50a  Qn50b  Qn50c  Qn50d  Qn50e |
| 51. | Je, unafahamu magonjwa yoyote yasiyo ya kuambukizwa yanayohusiana na kiasi cha mafuta watu wanayokula?  Ndiyo 2. Hapana 3. Sina hakika |  | Qn51 |
| 52. | Kama ndiyo, je ni magonjwa yapi yasiyoyakuambukiza yanahusiannishwa na ulaji wa mafuta mengi?   1. Shinikizo la damu la juu 2. Kisukari 3. Kansa 4. Sijui 5. Nyingine (taja)______________________ |  | Qn52a  Qn52b  Qn52c  Qn52d  Qn52e |
| 53. | Je, unafahamu magonjwa yoyote yasiyo ya kuambukizwa yanayohusiana na ulaji mdogo wa vyakula vyenye nyuzinyuzi?   1. Ndiyo 2. Hapana 3. Sina hakika |  | Qn53 |
| 54. | Kama ndiyo, je ni magonjwa yapi yasiyoyakuambukiza yanahusianishwa na ulaji mdogo wa vyakula vyenye nyuzinyuzi?   1. Shinikizo la damu la juu 2. Kisukari 3. Kansa 4. Sijui 5. Nyingine (taja)______________________ |  | Qn54a  Qn54b  Qn54c  Qn54d  Qn54e |
| 55. | Je, unafahamu magonjwa yoyote yasiyo ya kuambukizwa yanayohusiana unywaji wa pombe?  1.Ndiyo 2. Hapana 3. Sina hakika |  | Qn55 |
| 56. | Kama ndiyo, je ni magonjwa yapi yasiyoyakuambukiza yanahusianishwa na unywaji wa pombe?   1. Shinikizo la damu la juu 2. Kisukari 3. Kansa 4. Sijui 5. Nyingine (taja)______________________ |  | Qn56a  Qn56b  Qn56c  Qn56d  Qn56e |

**Sehemu E1: Upimaji wa Tabia – Utumiaji wa Tumbaku**

***Sasa nitakuuliza maswali yanayohusiana na tabia/mazoea mbalimbali yanayohusu afya. Haya yanahusisha masuala ya uvutaji sigara/tumbaku, unywaji wa pombe, ulaji wa matunda ma mbogamboga na mazoezi ya mwili. Tuanze na sigara/tumbaku.***

| **Namba ya swali** | **Swali** | **Jibu** | **Code** |
| --- | --- | --- | --- |
| 57. | Je, kwa sasa unavuta aina yoyote ya tumbaku kama vile sigara, kiko, sigara ya kusokota n.k? 1. Ndiyo 2. Hapana |  | Qn57 |
| 58. | **Kama Ndio,** Kwa sasa unavuta sigara au tumbaku **kila siku**? |  | Qn58 |
| 59. | Ulikuwa na umri gani ulipoanza kuvuta sigara kila siku kwa mara ya kwanza? __________________  Sijui: 77 |  | Qn59 |
| 60. | Unakumbuka ni muda gani uliopita?(JIBU MOJA TU)   1. Kwa miaka_______________________ 2. AU Kwa miezi______________ 3. AU Kwa wiki_____________ 4. Sikumbuki____________ |  | Qn60a  Qn60b  Qn60c  Qn60d |
| 61. | Kwa wastani, unavuta kiasi gani kwa siku cha kila moja ya aina zifuatazo za tumbaku? |  |  |
|  | 1. Sigara zinazotengenezwa viwandani __________ |  | Qn61a |
|  | 1. Sigara /tumbaku za kusokota kwa mikono __________ |  | Qn61b |
|  | 1. Kiko kilichojazwa tumbaku/sigara __________ |  | Qn61c |
|  | 1. Biri(Cigars)__________ |  | Qn61d |
|  | 1. Aina nyingine (taja)______________________________ |  | Qn61e |
| 62. | Hapo zamani, **ulishawahi** kuvuta sigara/tumbaku **kila siku**?  1. Ndiyo 2. Hapana |  | Qn62 |
| 63. | **Kama Ndio**, ulikuwa na miaka mingapi **ulipoacha** kuvuta sigara/tumbaku **kila siku**? |  | Qn63 |
| 64. | **Ni muda gani umepita** tangu ulipoacha kuvuta sigara/tumbaku kila siku? *(JIBU MOJA TU)*   1. Kwa miaka_______________________ 2. AU Kwa miezi______________ 3. AU Kwa wiki_____________ 4. Sikumbuki____________ |  | Qn64a  Qn64b  Qn64c  Qn64d |
| 65 | Kwa sasa unatumia aina yoyote ya tumbaku isiyo ya kuvuta kama vile ugoro, ‘kuber’ n.k.?  1. Ndiyo 2. Hapana |  | Qn65 |
| 66. | Ndani ya siku saba zilizopita, ni siku ngapi mtu mnayeishi naye ndani ya nyumba amevuta sigara ukiwepo?  1. Ndiyo 2. Hapana |  | Qn66 |
| 67. | Ndani ya siku saba zilizopita, ni siku ngapi mtu amevuta sigara kwenye eneo lisilo la wazi kazini ukiwepo?  1. Ndiyo 2. Hapana |  | Qn67 |

**Sehemu E2: Taarifa za tabia– Utumiaji wa Pombe/Vileo**

***Maswali yafuatayo yanauliza kuhusu utumiaji wa pombe/Vileo***

| **Namba ya swali** | **Swali** | **Jibu** | **Code** |
| --- | --- | --- | --- |
| 68. | Je, umewahi kutumia kinywaji chenye kilevi (kama vile bia, mvinyo, pombe kali au pombe ya kienyeji) katika **miezi 12 iliyopita**?  *(TUMIA kadi ya kielelezo AU TOA MIFANO)*   1. Ndiyo 2.Hapana |  | Qn68 |
| 69. | Je umekunywa kinywaji chenye kilevi ndani ya miezi 12 iliyopita?   1. Ndiyo 2.Hapana |  | Qn69 |
| 70. | Katika miezi 12 iliyopita, ni mara ngapi umekunywa angalau kinywaji kimoja chenye kilevi?( MSOMEE MAJIBU, TUMIA kadi ya kielelezo)  1.Kila siku  2.Siku 5-6 kwa juma  3.Siku 1-4 kwa juma  4.Siku 1-3 kwa mwezi  6.Chini ya mara 1 kwa mwezi |  | Qn70 |
| 71 | Umetumia kinywaji chenye kilevi (kama vile bia, mvinyo, pombe kali au pombe ya kienyeji) katika **siku 30 zilizopita**? *(TUMIA kadi ya kielelezo AU TOA MIFANO)*   1. Ndiyo 2.Hapana |  | Qn71 |
| 72. | Katika **siku 30** zilizopita, **ni mara ngapi** umekunywa angalau kinywaji kimoja chenye kilevi?  Idadi______________Sikumbuki (77) |  | Qn72 |
| 73. | Katika siku 30 zilizopita, ulipokunywa kinywaji chenye kilevi, kwa makisio ulikunywa vinywaji vingapi (standard drinks) kwa mkupuo  (*TUMIA KADI YA KIELELEZO)*  Idadi______________Sikumbuki (77) |  | Qn73 |
| 74. | Katika siku 30 zilizopita , ulipokunywa kinywaji chenye kilevi , ni kiasi gani **kikubwa** ulichokunywa (standard drink) siku uliyokunywa zaidi kwenye mkupuo mmoja ukijumlisha vinywaji vyote? |  | Qn74 |
| 75. | Katika siku 30 zilizopita, ni mara ngapi umekunywa Kinywaji chenye kilevi (standard drink);   1. **Kwa wanaume**: 5 au zaidi _______ 2. **Kwa wanawake**: 4 au zaidi _______   Kwenye mkupuo mmoja? |  | Qn75 |

**Sehemu E3: Taarifa za tabia– Lishe**

***Maswali yafuatayo yanauliza kuhusu matunda na mbogamboga ambazo unakula mara kwa mara. Hapa nina kadi ya maelezo ya lishe yenye mifano ya matunda na mbogamboga zinazopatikana katika maeneo yako. Kila picha inawakilisha kipimo kimoja. Unapojibu maswali haya tafadhali fikiria wiki moja ya kawaida katika mwaka uliopita***

| **Namba ya swali** | **Swali** | **Jibu** | **Code** |
| --- | --- | --- | --- |
| 76. | Kwa kawaida ni siku ngapi ndani ya wiki moja unakula **matunda**? *(TUMIA SHOWCARD*)  Idadi ya siku_____________Sifahamu (77) |  | Qn76 |
| 77. | Unakula matunda kipimo gani katika moja ya siku hizo? *(TUMIA SHOWCARD)*  Idadi ya siku_____________Sifahamu (77) |  | Qn77 |
| 78. | Kwa kawaida ni siku ngapi ndani ya wiki moja unakula **mbogamboga**? *(TUMIA SHOWCARD*)  Idadi ya siku_____________Sifahamu (77) |  | Qn78 |
| 79. | Unakula mbogamboga kipimo gani katika moja ya siku hizo? *(TUMIA SHOWCARD)*  Idadi ya siku_____________Sifahamu (77) |  | Qn79 |
| 80. | Ni aina gani ya **mafuta ya kupikia chakula** ambayo mnatumia mara kwa mara katika kaya yenu?   1. Mafuta ya wanyama 2. Mafuta ya mimea 3. Mafuta mchanganyiko 4. Sifuhamu |  | Qn80 |
| 81. | Kwa wastani huwa unakula milo mingapi kwa wiki ambayo haijaandaliwa nyumbani? Mlo hapa ni kifungua kinywa, mlo wa mchana na ule wa jioni?  Idadi ya siku_____________Sifahamu (77) |  | Qn81 |
| 82. | Je huwa unaongeza chumvi kwenye chakula kama haijakolea vizuri?   1. Hapana 2. Ndiyo |  | Qn82 |

**Sehemu E4: Taarifa za tabia– Mazoezi ya viungo**

*Sasa nitaendelea kukuuliza kuhusu muda unaotumia kufanya shughuli mbalimbali zinazohusu mazoezi ya viungo katika wiki moja ya kawaida. Tafadhali ujibu maswali haya hata kama unadhani wewe si mtu wa kufanya mazoezi mara kwa mara. Kwanza tafakari kuhusu muda unaotumia kufanya kazi. Tunaposema kazi tunamaanisha shughuli zozote unazofanya zikiwemo zinazokuingizia kipato na zisizokuingizia kipato, mfano kuvua samaki, masomo, shughuli za nyumbani, kilimo, kuvuna mazao, kutafuta kazi n.k. Katika kujibu maswali haya 'shughuli za kutumia nguvu – kasi sana ni shughuli ambazo zinahitaji nguvu nyingi na husababisha ongezeko kubwa katika kupumua au mapigo ya moyo, shughuli za kutumia nguvu-kasi kiasi ‘ ni shughuli zinazohitaji nguvu ya kiasi na husababisha ongezeko dogo katika kupumua na mapigo ya moyo.*

| **Namba ya swali** | **Swali** | **Jibu** | **Code** |
| --- | --- | --- | --- |
| **Mazoezi wakati wa kazi ya kipato chako cha kudumu** | | | |
| 83. | Je, kazi yako inahusisha shughuli za kutumia nguvu ambazo zinaongeza kasi ya kupumua na mapigo ya moyo kama vile kubeba mizigo mizito, kumwaga zege, kupiga kokoto, kuchota maji, kusomba mazao, kilimo au kazi za ujenzi kwa angalau dakika 10 mfululizo?*TUMIA SHOWCARD)*  1.Hapana 2. Ndiyo |  | Qn83 |
| 84. | Katika wiki ya kawaida, ni kwa siku ngapi unafanya shughuli hizo za kutumia nguvu nyingi katika kazi yako?  Idadi________________________Sifuhamu |  | Qn84 |
| 85. | Unatumia muda gani kufanya shughuli za nguvu katika siku moja ya kazi? | a) Hours : ___  b)Minutes:___ | Qn85a  Qn85b |
| 86. | azi yako inahusisha shughuli za kutumia nguvu kiasi ambazo zinaongeza kidogo kasi ya kupumua na mapigo ya moyo kama vile kutembea kwa haraka, kubeba mizigo isiyo mizito, kudeki, kufagia, kuosha vyombo, kufua, kupika, kupiga pasi, kuvuna mazao kwa angalau dakika 10 mfululizo? *(TUMIA SHOWCARD)*  1.Hapana 2. Ndiyo |  | Qn86 |
| 87. | Katika wiki ya kawaida, ni kwa siku ngapi unafanya shughuli hizo za kutumia nguvu kiasi katika kazi yako?  Idadi________________________Sifuhamu |  | Qn87 |
| 88. | Unatumia muda gani kufanya shughuli za nguvu kiasi katika siku moja ya kazi? | a) Hours : ___  b)Minutes:___ | Qn88a  Qn88b |
| **Mazoezi kupitia Safari ya kwenda na kurudi tokea sehemu moja hadi nyingine** | | | |
| Maswali yafuatayo hayahusishi shughuli unazofanya wakati wa kazi ambazo umeshazitaja.  Sasa ningependa kukuuliza kuhusu aina ya usafiri unaotumia kwenda sehemu mbalimbali kama vile sokoni, shambani, kanisani, msikitini n.k. | | | |
| 89. | Una kawaida ya kutembea kwa miguu au kwa kutumia baiskeli kwa angalau dakika 10 mfululizo wakati unapokwenda mahali fulani?  1.Hapana 2. Ndiyo |  | Qn89 |
| 90. | Katika wiki ya kawaida, unatumia siku ngapi kutembea kwa miguu au kwa kutumia baiskeli kwa angalau dakika 10 mfululizo wakati unapokwenda mahali fulani?  Idadi________________________Sifuhamu |  | Qn90 |
| 91. | Unatumia muda gani kutembea kwa miguu au kwa kutumia baiskeli katika siku moja ya kawaida? | a) Masaa: __  b)Dakika:___ | Qn91a  Qn91b |
| **Mazoezi ya viungo wakati wa mapumziko (ya hiari)** | | | |
| Sasa nitakuuliza maswali yahusuyo shughuli mbalimbali unazofanya wakati wako wa mapumziko, kwa mfano mazoezi ya viungo, michezo mbalimbali n.k. Usijumuishe shughuli unazofanya wakati wa kazi au kusafiri ambazo umeshajitaja hapo awali. | | | |
| 92. | Una kawaida ya kushiriki katika shughuli za michezo au mazoezi ambazo zinaongeza kwa kiasi kikubwa kasi ya kupumua au mapigo ya moyo kama vile kukimbia, kuruka kichura, kuinama na kuinuka, kuruka viunzi, kucheza nmpira kwa angalau dakika 10 mfululizo? (TUMIA SHOWCARD)  1.Ndiyo 2. Hapana |  | Qn92 |
| **Namba ya swali** | **Swali** | **Jibu** | **Code** |
| 93. | Katika wiki ya kawaida, unatumia siku ngapi kufanya shughuli hizo za michezo au mazoezi?  Idadi ya siku_______________________ |  | Qn93 |
| 94. | Katika siku ya kawaida, unatumia muda gani kufanya shughuli hizo za michezo au mazoezi? | a) Masaa: __  b)Dakika:___ | Qn94a  Qn94b |
| 95. | Una kawaida ya kushiriki katika shughuli za michezo au mazoezi ambazo zinaongeza kwa kiasi kidogo kasi ya kupumua au mapigo ya moyo kama vile kuendesha, baiskeli, kutembea, kuogelea, kuimba kwa vitendo kwa angalau dakika 10 mfululizo? *(TUMIA SHOWCARD)*  1.Ndiyo 2. Hapana |  | Qn95 |
| 96. | Katika wiki ya kawaida, unatumia siku ngapi kufanya shughuli hizo za michezo au mazoezi?  Idadi ya siku___________________________ |  | Qn96 |
| 97. | Katika siku ya kawaida, unatumia muda gani kufanya shughuli hizo za michezo au mazoezi? | a) Masaa: __  b) Dakika:___ | Qn97a  Qn97b |
| **Tabia ya kukaa pasipo kujishughulisha** | | | |
| Swali lifuatalo linahusu muda uliotumia kukaa au kujinyoosha/kujiegemeza kujilaza wakati wa kazi, katika usafiri, kukaa na marafiki, kuangalia televisheni n.k. lakini bila kujumuisha muda uliotumia kulala. *(TUMIA SHOWCARD)* | | | |
| 98. | Kwa kawaida unatumia muda gani kukaa au kujinyoosha kujilaza katika siku moja mfano ukiwa unaangalia TV, unasikiliza redio, unasoma gazeti au vitabu n.k? | a) Masaa: __  b) Dakika:___ | Qn98a  Qn98b |

**Sehemu E6: Step 1: Historia ya Ongezeko la Shinikizo la Damu**

| 99. | Je umeshawahi kupimwa na daktari au mtaalam wa afya?  1.Ndiyo 2. Hapana |  | Qn99 |
| --- | --- | --- | --- |
| 100. | Je umeshawahi kuambiwa na daktari au mtaalam wa afya kuwa una shinikizo la damu, au presha yako iko juu kuliko kawaida?  1.Ndiyo 2. Hapana |  | Qn100 |
| 101. | Je umeambiwa hivyo ndani ya miezi 12 iliyopita?  1.Ndiyo 2. Hapana |  | Qn101 |

**Sehemu E7: Step 1: Historia ya Kisukari**

| 102. | Umewahi kupimwa kiwango cha sukari katika damu?  1. Ndiyo 2. Hapana |  | Qn102 |
| --- | --- | --- | --- |
| 103. | Katika miezi 12 iliyopita, umewahi kuambiwa na daktari au mtaalamu mwingine wa afya kuwa una ugonjwa wa kisukari?  1.Ndiyo 2. Hapana |  | Qn103 |
| 104. | Je umeambiwa hivyo ndani ya miezi 12?  1.Ndiyo 2. Hapana |  | Qn104 |

**Sehemu F: Step 2 Vipimo vya mwili visivyohusisha kutoa damu**

| **Namba ya swali** | **Swali** | **Jibu** | **Code** |
| --- | --- | --- | --- |
| **Urefu na Uzito** | | | |
| 105. | Utambulisho wa Mhojaji | a)Urefu:_____ | Qn105a |
|  |  | b) Uzito:______ | Qn105b |
| 106. | Urefu (cm) | ______.____ | Qn106 |
| 107. | Uzito (kgs) *Ikiwa uzito umezidi uwezo wa mizani andika 666.6* | ______.____ | Qn107 |
| **Kiuno** | | | |
| 108. | Utambulisho wa kifaa cha kupimia kiuno | ___________ | Qn108 |
| 109. | Mzunguko wa Kiuno (cm) | ______.____ | Qn109 |
| Blood Pressure | | | |
| 110. | Utambulisho wa Mhojaji |  | Qn110 |
| 111. | Ukubwa wa cuffitakayotumika:  1. Ndogo 2. Ya kati 3. Kubwa |  | Qn111 |
| 112. | Kipimo cha 1 a) Sistoliki ( mmHg) | __________ | Qn112a |
|  | b) Diastoliki (mmHg) | __________ | Qn112b |
| 113. | Kipimo cha 2 a) Sistoliki ( mmHg) | __________ | Qn113a |
|  | b) Diastoliki (mmHg) | __________ | Qn113b |
| 114. | Kipimo cha 3 a) Sistoliki ( mmHg) | __________ | Qn114a |
|  | b) Diastoliki (mmHg) | __________ | Qn114b |
| 115. | Katika wiki 2 zilizopita, umetumia dawa yoyote ya ongezeko la shinikizo la damu kama ulivyoandikiwa na daktari au mtaalamu mwingine wa afya?  1. Ndiyo 2. Hapana |  | Qn115 |
| **Mzunguko wa Nyonga** | | | |
| 116. | Mzunguko wa Nyonga (sm) | __________ | Qn116 |

**Sehemu G: Step 3 Vipimo vya mwili vinavyohusisha kutoa damu**

| **Sukari katika Damu** | | | |
| --- | --- | --- | --- |
| 117. | Ndani ya masaa 12 yaliyopita, umekula au kunywa kitu chochote zaidi ya maji?  1. Ndiyo 2. Hapana |  | B1 |
| 118. | Majina ya mpimaji (vifupi) | ___________ | Qn117 |
| 119. | Utambulisho wa kifaa cha kupimia | ___________ | Qn118 |
| 120. | Muda kipimo kilipochukuliwa (kwa masaa 24) | a)Masaa______  b)Dakika______ | Qn119 |
| 121. | Kiwango cha sukari katika damu kabla ya kula: *(mmol/l)* |  | Qn120 |
| 122. | Kwa siku ya leo, je umetumia dawa yeyote ya kisukari uliyopewa na mtumishi wa afya kama insulin au vidonge vya kushusha sukari?  1. Ndiyo 2. Hapana |  | Qn121 |

| **Namba ya swali** | **Swali** | **Jibu** | **Code** |
| --- | --- | --- | --- |
| **Mafuta katika damu** | | | |
| 123. | Utambulisho wa Mpimaji |  | Qn123 |
| 124. | Lehemu *(TC) (mmol/l)* |  | Qn124 |
| 125. | Katika wiki mbili zilizopita, je umetumia dawa yeyote ya vidonge uliyopewa na mtumishi wa afya vya kushusha kiasi cha lehemu kwenye damu?  1. Ndiyo 2. Hapana |  | Qn125 |
| 126. | Triglycerides  *(mmol/l)* |  | Qn126 |
| 127. | Katika wiki mbili zilizopita, je umetumia dawa yeyote ya vidonge uliyopewa na mtumishi wa afya vya kushusha kiasi cha Triglycerides kwenye damu?  1. Ndiyo 2. Hapana |  | Qn127 |
| 128. | High Density Lipoprotein *(mmol/l)* |  | Qn128 |
| 129. | Katika wiki mbili zilizopita, je umetumia dawa yeyote ya vidonge uliyopewa na mtumishi wa afya vya kushusha kiasi cha High Density Lipoprotein kwenye damu?  1. Ndiyo 2. Hapana |  | Qn129 |
| 130. | Low Density Lipoprotein *mmol/l)* |  | Qn130 |
| 131. | Katika wiki mbili zilizopita, je umetumia dawa yeyote ya vidonge uliyopewa na mtumishi wa afya vya kushusha kiasi cha Low Density Lipoprotein kwenye damu?  1. Ndiyo 2. Hapana |  | Qn131 |

**Tathimini ya mwisho: Maswali yote juu yatakuwepo na nyongeza chini:**

| **Namba ya swali** | **Swali** | **Jibu** | **Code** |
| --- | --- | --- | --- |
| **Sahani ya afya (kwa kundi la jaribio tu)** | | | |
| 132. | Wastani wa sahani za kiafya alizokula kwa wiki? | _______ | Qn132 |

**Mambo ya kijamii na kiuchumi na kiutamaduni ambayo yanaweza kuhamasisha watu kuwa na muitikio na kuzingatia sahani ya kiafya**

| **Namba ya swali** | **Swali** | **Jibu** | **Code** |
| --- | --- | --- | --- |
| 133. | Kulingana na uzoefu wako unadhani ni changamoto zipi za kiuchumi zinaadhiri au zitaadhiri watu kutumia sahani ya kiafya? |  | Qn133 |
| 134. | In this place do you think there some cultural factors which may hinder you from not consuming healthy plate?  Katika eneo hili unafikiri kuna changamoto za kimila zinazoweza sababisha usitumie sahani ya afya? |  | Qn134 |
| 135. | Kama zipi. Je ni zipi? _______________________ |  | Qn135 |
| 136. | Unapendekeza nini kifanyike ili kuboresha uwepo, kuweza kununua na upatikanaji wa sahani ya afya kutoka kwa wauzaji wa chakula mitaani? |  |  |
|  | 1. Uwepo: |  | Qn136a |
|  | 1. Uwezo wa kununua: |  | Qn136b |
|  | 1. Upatikananji: |  | Qn136c |

**Maswali ya mjadala wa kina (Wauzaji wa chakula mtaani)**

1. Nini uelewa wenu kuhusiana na kisukari cha aina ya pili? (dodosa kuhusu: vihatarishi ,dalili, njia kujikinga na madhara ya kisukari)
2. Je kuna uhusiano gani kati ya kisukari cha aina ya pili na lishe ya mtu? (dodosa kuhusu: chakula chenye wanga mwingi, protini, sukari, chumvi, mafuta, mbogamboga na matunda kidogo)
3. Kama mtu ana vihatarishi kwa kiwango kikubwa vya kisukari mfano vya kitabia na vimengenywa (kimetaboliki), je mnadhani kuna nafasi ya pili ya kuweza kuzuia ugonjwa wa kisukari usimpate(kama ndiyo au hapana ni kwanini)
4. Ili kumkinga mtu hasipate vihatarishi vya vimengenywa/kimetaboliki vya kisukari; je ni kiasi gani kizuri cha mchanganyiko wa vyakula mbalimbali mtu anapaswa mumuuzie kwenye sahani moja anayokula? (dodosa kuhusu: kiasi cha wanga, protini, matunda na mbogamboga)
5. Mnafikiri kuna changamoto gani mtakumbana nazo iwapo serikali itatoa maelekezo kwenu ili muanze kuuza sahani ya afya yaani saheni yenye mchanganyiko wa vakula mbalimbali kwa kipimo kinachotakiwa cha wanga, protini, matunda na mbogamboga? (dodosa kuhusu: changamoto za kiuchumi, mazingira and kiutamaduni)

**Maswali ya mjadala wa kina (Wateja wa chakula mtaani)**

1. Nini uelewa wenu kuhusiana na kisukari cha aina ya pili? (dodosa kuhusu: vihatarishi ,dalili, njia kujikinga na madhara ya kisukari)
2. Je kuna uhusiano gani kati ya kisukari cha aina ya pili na lishe ya mtu? (dodosa kuhusu: chakula chenye wanga mwingi, protini, sukari, chumvi, mafuta, mbogamboga na matunda kidogo)
3. Kama mtu ana vihatarishi kwa kiwango kikubwa vya kisukari mfano vya kitabia na vimengenywa (kimetaboliki), je mnadhani kuna nafasi ya pili ya kuweza kuzuia ugonjwa wa kisukari usimpate(kama ndiyo au hapana ni kwanini)
4. Ili kumkinga mtu hasipate vihatarishi vya vimengenywa/kimetaboliki vya kisukari; je ni kiasi gani kizuri cha mchanganyiko wa vyakula mbalimbali mtu anapaswa mumuuzie kwenye sahani moja anayokula? (dodosa kuhusu: kiasi cha wanga, protini, matunda na mbogamboga)
5. Mnafikiri kuna changamoto gani mtakumbana nazo iwapo mtoa huduma za afya atawaagiza kuanza kununua ahani ya afya yaani saheni yenye mchanganyiko wa vakula mbalimbali kwa kipimo kinachotakiwa cha wanga, protini, matunda na mbogamboga kwa ajili ya kujikinga na vihatarishi vya kisukari? (dodosa kuhusu: changamoto za kiuchumi, mazingira and kiutamaduni)

**Dodoso la kina (Wadau wa lishe)**

1. Kutokana na uzoefu wako kwa sasa una maoni gani kuhusu mwenendo (trend) wa vihatarishi vya kitabia, vimengenyo/kimetabolic na vya kurithi vya kisukari katika kata, wilaya/nchi?
2. Nini maoni yako kuhusu ubora wa chakula kinachouzwa mtaani ukiusishanisha na vihatarishi vya vimengenyo/kimetaboliki vya kisukari (dodosa kuhusu:kiasi cha wanga kinachouzwa, sukari, chunvi, mafuta, kiasi cha matunda na mbogamboga)
3. Ni mikakati gani ipo ya kuhakikisha wauza chakula cha mtaani wanakuwa na kipaumbele cha ubora wa chakula wanachouza ukiusishanisa na kuzuia kisukari?
4. Kwa maoni yako ni changamoto zipi zinaweza kuadhiri mikakati ya uwepo, uwezo wa kununu na upatikanaji wa sahani ya afya yaani sahani yenye mnchanganyiko unaotakiwa wa kiasi cha wanga, protin, matunda na mbogamboga? (Dodosa kuhusu: sera, miongozo, changamoto za kiuchumi, kijamii, kimazingira na kiutamaduni)

#### Participant Feedback Form for physical measurements results

| We would like to provide you with an overview of your results from the physical measurements. |
| --- |

| Blood pressure | Systolic: ______________________ mmHg (average of reading 2 and 3) |
| --- | --- |
|  | Diastolic: _____________________ mmHg (average of reading 2 and 3) |
| Blood pressure classification | Normal (SBP<140 and DBP<90)  Elevated (SBP 140-159 and/or DBP 90-99)  Raised (SBP≥160 and/or DBP≥100)  Currently on medication |

| Height | Height: ______________________ cm |
| --- | --- |
| Weight | Weight: ______________________ kg |
| Body Mass Index | BMI: ________________________ kg/m^2^ (weight in kg divided by height in meters squared; ex. for height 170 cm and weight 68 kg BMI=(68/(1.7^2^))=23.5) |
| BMI classification | Underweight (BMI<18.5)  Normal weight (BMI 18.5-24.9)  Overweight (BMI 25-29.9)  Obese (BMI≥30) |

| Waist circumference | Waist: ______________________ cm |
| --- | --- |
| Hip circumference | Hip: ________________________ cm |

#### Participant Feedback for biochemical tests results

Dear Participant,

| We would like to provide you with an overview of your results from the biochemical measurements. |
| --- |

| Fasting blood glucose | Fasting blood glucose: ______________________ mmol/l |
| --- | --- |
| Fasting blood glucose classification | Normal (<7.0 mmol/l)  Raised (≥7.0 mmol/l)  Currently on medication |

| Total blood cholesterol | Total cholesterol: ______________________ mmol/l |
| --- | --- |
| Total blood cholesterol classification | Normal (<5.0 mmol/l)  Elevated (5.0-6.1 mmol/l)  High (≥6.2 mmol/l) |
| HDL cholesterol | HDL cholesterol: ______________________ mmol/l |
| HDL cholesterol classification | Normal (≥1.03 mmol/l for Men, ≥ 1.29 mmol/l for Women)  Low (<1.03 mmol/l for Men, <1.29 for Women) |
| Triglycerides | Triglycerides: ______________________ mmol/l |
| Triglycerides classification | Normal (<2.0 mmol/l)  Raised (≥2.0 mmol/l) |

#### Focus Group Discussion Questions (Street Foods vendors)

1. What do you know about type2 diabetes (probe on: risk factors, symptoms, prevention strategies, complications)
2. How diabetes is linked to nutrition values of food consumed by a person (probe on: foods with excess carbohydrate, protein, sugars, salt, fats, less fruits and vegetables)
3. If a person has increased behavioural, metabolic and family history risks for diabetes, do you think there is a second chance for preventing him/her from disease manifestation? (If Yes/No explain why?)
4. In order to prevent someone from increased metabolic risk factors of diabetes, what is the best amount of combination of food varieties to be consumed per each plate you sell to your customers (probe on: amount of carbohydrate, protein, sugars, salt, fats, less fruits and vegetables).
5. What are the factors think you will face if the government will instruct the food vendors to serve a plate with the best recommended amount of food varieties i.e. amount of carbohydrate, protein, sugars, salt, fats, less fruits and vegetables (probe on: socio-economic, environmental and cultural factors).

**Focus Group Discussion Questions (Street Food consumers)**

1. What do you know about type2 diabetes (probe on: risk factors, symptoms, prevention strategies, complications)
2. How diabetes is linked to nutrition values of food consumed by a person (probe on: foods with excess carbohydrate, protein, sugars, salt, fats, less fruits and vegetables)
3. If a person has increased behavioural, metabolic and family history risks for diabetes, do you think there is a second chance for preventing him/her from disease manifestation? (If Yes/No explain why?)
4. In order to prevent someone from increased metabolic risk factors of diabetes, what is the best amount of combination of food varieties to be consumed per each plate you sell to your customers (probe on: amount of carbohydrate, protein, sugars, salt, fats, less fruits and vegetables).
5. What are the factors think you will face if the health service providers will instruct you to consume an healthy food plate i.e. a plate with the best recommended amount of food varieties i.e. amount of carbohydrate, protein, sugars, salt, fats, less fruits and vegetables (probe on: socio-economic, environmental and cultural factors).

**In-depth interview (key government stakeholders)**

1. Based on your current information what can you comment on trend of behavioural, metabolic and family history risks for diabetes in this ward/district /county?
2. What is your opinion on the nutritional values of foods served by street food vendors in relation to metabolic risks for diabetes? (Probe on: amount of carbohydrate, protein, sugars, salt, fats, less fruits and vegetables served).
3. What strategies are in place to ensure that street foods vendors consider the nutritional values of foods they serve to their clients?
4. What are the factors think will affect the strategies for ensuring availability, accessibility and affordability of healthy food plate i.e. a plate with the best recommended amount of food varieties i.e. amount of carbohydrate, protein, sugars, salt, fats, less fruits and vegetables (probe on: policy, socio-economic, environmental and cultural factors)

# Appendix C: Consent forms

**Consent Form for Intervention Participants**

**Participant Identification (PID)____________________**

I would like to take this opportunity to thank you for taking your time and agreeing to talk to me today. I would also like to identify myself and inform you the purpose of our meeting here.

My name is ...................................................................................I work on behalf of PhD student who is registered at Kilimanjaro Christian Medical College and an employee of the National Institute for Medical Research in Tanzania.

Introduction: The world is experiencing unprecedented increase of type2 diabetes attributed to unhealthy diet, low income to afford nutritious food, and low knowledge on nutritional values of food. The significant number of people in urban areas relies on food supplied by Urban Street Food Vendors who rarely take into account nutritional value of food thus, putting urban dwellers at risk to get diabetes. The situation is also compromised by the fact that the available food quality control and enforcement mechanisms focus on monitoring risk factors linked to infectious diseases only. Despite of having substantial evidence on the burden of the problem; little has been invested on possible prevention interventions to reduce the problem which is currently imposing exorbitant costs to the country health systems and family level. The aim of this study is to implement and evaluate the effectiveness of type2 diabetes metabolic risk factors reduction intervention package and developing a model for predicting metabolic risk factors among urban street cooked food consumers in Tanzania. A total of 556 street food consumers, at least 50 food vendors and 10 stakeholders will be recruited to participate in this study. This research funded by Afrique One ASPIRE under One Health Consortium.

We ask you to allow us to ask you general questions regarding your knowledge and perception on type2 diabetes, your daily lifestyle habits (eating habits, body infections, participation in physical activities, smoking, alcohol drinking etc). Information on your history on diseases and physical measurements (height, weight, hips and waist circumference and blood pressure) will be also collected. We will also take a little blood from arm and finger to measure the level of sugar and fat in your blood. If you have not fasted for at least 8 hours; blood tests will be made tomorrow morning because we want to take blood before you plant something. You will receive health education (risk factors, symptoms, prevention, complication and diabetes and dietary) which will be delivered through brochures, group education and if possible through your mobile phone technology. You will be also required to consume a healthy plate model from your normal food vendor for the duration of not less than three months of trial. The project will cover the costs for fruits and vegetables and you will pay the normal price you are used to cover the prices for carbohydrate and protein. After three months of an intervention we are going to collect similar information, physical and blood measurements narrated above. This evaluation will enable us to assess the efficacy of the prevention package on behavioural and metabolic risk factors of type 2 diabetes. I would like to prove to you that you will not be taken for the extra tests apart from the ones I have mentioned above.

Findings from this study will be used in finding ways to improve the nutrition values of street cooked foods and thus contribute in reducing the risk factors of type2 diabetes among consumers.

The information you provide is totally confidential and will not be disclosed to anyone. It will only be used for research purposes. Your name, address, and other personal information will be removed from the instrument, and only a code will be used to connect your name and your answers without identifying you. You may be contacted by the survey team again only if it is necessary to complete the information on the survey.

Your participation is voluntary and you can withdraw from the survey after having agreed to participate. You are free to refuse to answer any question that is asked in the questionnaire. If you have any questions about this survey you may ask me or contact [name of institution and contact details] or [Principal Investigator at site].

This investigation has been approved and permission granted from all relevant levels including the National Institute of Medical Research (NIMR), the Ministry of Health, Regions, District, ward and market places.

If you have any inquiries or any questions associated with this study you can contact the principal investigator Mr. Gibson Kagaruki (0757 404142/0712136200/0784593870). For communication with the Medical Research Coordination Committee of the National Institute for Medical Research (NIMR), use the following address.; P.O.Box 9653, DSM, Tel: 022 2121400.

Signing this consent indicates that you understand what will be expected of you and are willing to participate in this survey.

Read by Participant Interviewer: 1. Agreed 2. Refused

**Consent to participate**: I hereby provide INFORMED CONSENT for the study.

**Name: Signature/Thumb**

Name of interviewer_________________________ __________________________

Name of participant__________________________ __________________________

Witness (for illiteracy participant)_________________ ____________________________

Date______________________

**Consent Form for Control Participants**

**Participant Identification (PID)____________________**

I would like to take this opportunity to thank you for taking your time and agreeing to talk to me today. I would also like to identify myself and inform you the purpose of our meeting here.

My name is ...................................................................................I work on behalf of PhD student who is registered at Kilimanjaro Christian Medical College and an employee of the National Institute for Medical Research in Tanzania.

Introduction: The world is experiencing unprecedented increase of type2 diabetes attributed to unhealthy diet, low income to afford nutritious food, and low knowledge on nutritional values of food. The significant number of people in urban areas relies on food supplied by Urban Street Food Vendors who rarely take into account nutritional value of food thus, putting urban dwellers at risk to get diabetes. The situation is also compromised by the fact that the available food quality control and enforcement mechanisms focus on monitoring risk factors linked to infectious diseases only. Despite of having substantial evidence on the burden of the problem; little has been invested on possible prevention interventions to reduce the problem which is currently imposing exorbitant costs to the country health systems and family level. The aim of this study is to implement and evaluate the effectiveness of type2 diabetes metabolic risk factors reduction intervention package and developing a model for predicting metabolic risk factors among urban street cooked food consumers in Tanzania. A total of 556 street food consumers, at least 50 food vendors and 10 stakeholders will be recruited to participate in this study. This research funded by Afrique One ASPIRE under One Health Consortium.

We ask you to allow us to ask you general questions regarding your knowledge and perception on type2 diabetes, your daily lifestyle habits (eating habits, body infections, participation in physical activities, smoking, alcohol drinking etc). Information on your history on diseases and physical measurements (height, weight, hips and waist circumference and blood pressure) will be also collected. We will also take a little blood from arm and finger to measure the level of sugar and fat in your blood. If you have not fasted for at least 8 hours; blood tests will be made tomorrow morning because we want to take blood before you plant something. You will receive health education (on risk factors, symptoms, prevention, complication and diabetes and dietary) which will be delivered through brochures, group education and if possible through your mobile phone technology. We request yo to remain getting food service from the same vendor for the duration of not less than three month. After three months of an intervention we are going to collect similar information, physical and blood measurements narrated above. This evaluation will enable us to assess the efficacy of the health education on behavioural and metabolic risk factors of type 2 diabetes. I would like to prove to you that you will not be taken for the extra tests apart from the ones I have mentioned above.

Findings from this study will be used in finding ways to improve the nutrition values of street cooked foods and thus contribute in reducing the risk factors of type2 diabetes among consumers.

The information you provide is totally confidential and will not be disclosed to anyone. It will only be used for research purposes. Your name, address, and other personal information will be removed from the instrument, and only a code will be used to connect your name and your answers without identifying you. You may be contacted by the survey team again only if it is necessary to complete the information on the survey.

Your participation is voluntary and you can withdraw from the survey after having agreed to participate. You are free to refuse to answer any question that is asked in the questionnaire. If you have any questions about this survey you may ask me or contact [name of institution and contact details] or [Principal Investigator at site].

This investigation has been approved and permission granted from all relevant levels including the National Institute of Medical Research (NIMR), the Ministry of Health, Regions, District, ward and market places.

If you have any inquiries or any questions associated with this study you can contact the principal investigator Mr. Gibson Kagaruki (0757 404142/0712136200/0784593870). For communication with the Medical Research Coordination Committee of the National Institute for Medical Research (NIMR), use the following address.; P.O.Box 9653, DSM, Tel: 022 2121400.

Signing this consent indicates that you understand what will be expected of you and are willing to participate in this survey.

Read by Participant Interviewer: 1. Agreed 2. Refused

**Consent to participate**: I hereby provide INFORMED CONSENT for the study.

**Name: Signature/Thumb**

Name of interviewer_________________________ __________________________

Name of participant__________________________ __________________________

Witness (for illiteracy participant)_________________ ____________________________

Date______________________

**Fomu ya ridhaa ya mshiriki-Kwenye jaribio**

**Namba ya Utambulisho ya Mshiriki**___________________________

Napenda kuchukuwa fursa hii kukushukuru wewe kwa kutenga muda wako na kukubali kuzungumza na mimi leo hii. Pia napenda kujitambulisha kwako na kukujulisha madhumuni ya kukutana kwetu hapa.

Jina langu ni …………………..…….………….. ni mfanyakazi katika Wizara ya Afya na Ustawi wa Jamii.

**Utangulizi**: Dunia inakabiliwa na ongezeko lisilo la kawaida la kisukari cha aina ya 2 kinachohusishwa ulaji usizingatia afya, kipato cha chini cha kumudu chakula chenye ubora, na uelewa mdogo juu ya chakula chenye ubora. Idadi kubwa ya watu katika maeneo ya mijini hutegemea chakula wauzaji wa mtaani ijapokuwa wauzaji na wanunuzi hawajazingatia mara kwa mara ubora wa chakula, hivyo kuweka watumiaji katika hatari ya kupata ugonjwa wa kisukari. Hali pia imeathiriwa na ukweli kwamba taasisi za kusimamia ubora wa chakula zimejikita kusimamia viatarishi vya magonjwa yanayoambukiza tu na kuweka kando vihatarishi vya magonjwa yasiyo ya kuambukiza. Japokuwa kuna taarifa za kutosha kuhusu kiwango cha tatizo la kisukari na vihatarishi vyake, taarifa za kitafiti kuhusu njia za kifanisi za kuzuia tatizo la kisukari na vihatarishi vyake; tatizo linalozidisha mzigo wa gharama kwa mifumo ya afya ya nchi na ngazi ya familia ni haba nchini. Lengo la utafiti huu ni kutekeleza na kutathmini njia ya kupunguza vimeng’enywa hatarishi vya aina ya pili ya ugonjwa wa kisukari, na kuandaa mwongozo wa kubaini hatari za vimeng’enywa miongoni mwa walaji wa vyakula vilivyopikwa vya mitaani nchini Tanzania. Jumla ya watumiaji wa chakula cha barabara 556, angalau wadau wa chakula 50 na wadau 10 watashiriki katika utafiti huu. Utafiti huu unafadhiliwa na **Afrique One ASPIRE Chini ya Mpango wa One Health.**

Tunaomba ruhusa kukuuliza maswali ya kiujumla, uelewa na mtazamo wako kuhusu ugonjwa wa kisukari, tabia zako za maisha ya kila siku (ulaji wako wa chakula, ushughulishaji wa mwili, uvutaji wa sigara, utumiaji wa kilevi n.k) historia yako juu ya magonjwa na vipimo vya mwili (urefu, uzito, ukubwa wa paja lako na ukubwa wa kiuno). Pia tutachukua damu kidogo kutoka kwenye mkono na kidole ili kuweza kupima kiwango cha sukari na mafuta ndani ya damu. Hili litafanyika kesho asubuhi kwa sababu tunataka kuchukua damu kabla hujala kitu. Utapaswa kushiriki katika elimu (juu ya vihatarishi vya kisukari, dalili, kujikinga, madhara na kisukari na lishe) itakayotolewa kwa njia ya vipeperushi na mafundisho ya vikundi na ikibidi hata kwa njia ya simu. Pia utapaswa kula sahani ya afya kwa muzaji wa chakula cha mtaani uliomzoea kwa kipindi cha miezi mitatu ya majaribio ambapo mradi utagharimia ghalama za matunda na mbogamboga na wewe utaendelea kulipia kiasi ulichozoea kulipa. Baada ya hapo utafanyiwa mahojiano na kufanyiwa vipimo kama hapo juu kuona kama elimu na sahani ya afya vina mchango katika kupunguza vihatarishi vya kitabia na kimetabolic vya kisukari. Napenda kukuthibitishia kuwa hautachukuliwa vipimo vya ziada ya nilivyokueleza.

Matokeo ya utafiti huu yatatumika kutafuta njia za kuboresha ubora wa chakula kinachopikwa na kuuzwa mitaani na hivyo kuchangia katika kupunguza viatarishi vya ugonjwa wa kisukari kwa watumiaji.

Taarifa utakzotoa ni siri kabisa na hazitatambulika kwa mtu yeyote. Zitatumika tu kwa madhumuni ya utafiti. Jina lako, anwani, na maelezo mengine ya kibinafsi yataondolewa kwenye kanzidata, ni namba ya utambulisho tu itatumika kuunganisha jina lako na majibu yako bila kukufahamu. Zaidi; timu ya watafiti yaweza kuwaliana nawe tena baadaye iwapo itatokea kuna taarifa zako zinaitajika.

Ushiriki wako ni wa hiari na unaweza kujiondoa kwenye utafiti baada ya kukubali kushiriki. Pia huko huru kukataa kujibu swali lolote ambalo linaulizwa katika dodoso hili. Ikiwa una maswali yoyote kuhusu utafiti huu unaweza kuniuliza mimi au kuwasiliana na [jina la taasisi na maelezo ya mawasiliano] au [Mtafiti Mkuu kwenye tovuti].

Uchunguzi huu umepitishwa na kuombewa ruhusa kwa ngazi zote zinazohusika ikiwemo Taasisi ya Taifa ya Utafiti wa Magonjwa ya Binadamu (NIMR), Wizara ya Afya, Mikoa, Wilaya, kata na ngazi ya uongozi wa soko.

Iwapo wakati wowote una maswali ya kuuliza au tatizo lolote linalohusiana na utafiti huu unaweza kuwasiliana na mtafiti mkuu (principle investigator) Mr. Gibson Benard Kagaruki (0757 404142/0784593870). Kwa mawasiliano na Kamati ya Maadili ya Utafiti, Taasisi ya Taifa ya Utafiti wa Magonjwa ya Binadamu (NIMR) wasiliana nao kwa anuani zifuatayo: P.O.Box 9653, DSM, namba ya simu: 022 2121400.

Ukiweka sahahi kwenye fomu hii ya ridhaa ni kuthibitisha kwamba unaelewa nini kinatatarajiwa kwako kwa upande wa taarifa na sampuli za vipimo na ni kuthihirisha kuwa una nia ya kushiriki katika utafiti huu.

Imesomwa na mhojaji, je mhojiwa amekubali kushiriki?: 1. Amekubali 2. Amekataa

Ridhaa ya kushiriki: Mimi hapa NIMETOA RIDHAA ya kushiriki utafiti huu.

**Jina: Sahihi/Dole Gumba**

Jila la mhojaji_________________________ ___________________

Jina la mshiriki__________________________ ___________________

Shahidi (kama mshiriki hajui kusoma)_________________ ___________________

Tarehe_______________________________

**Fomu ya ridhaa ya mshiriki-Asiye kwenye jaribio**

**Namba ya Utambulisho ya Mshiriki**___________________________

Napenda kuchukuwa fursa hii kukushukuru wewe kwa kutenga muda wako na kukubali kuzungumza na mimi leo hii. Pia napenda kujitambulisha kwako na kukujulisha madhumuni ya kukutana kwetu hapa.

Jina langu ni …………………..…….………….. ni mfanyakazi katika Wizara ya Afya na Ustawi wa Jamii.

**Utangulizi**: Dunia inakabiliwa na ongezeko lisilo la kawaida la kisukari cha aina ya 2 kinachohusishwa ulaji usizingatia afya, kipato cha chini cha kumudu chakula chenye ubora, na uelewa mdogo juu ya chakula chenye ubora. Idadi kubwa ya watu katika maeneo ya mijini hutegemea chakula wauzaji wa mtaani ijapokuwa wauzaji na wanunuzi hawajazingatia mara kwa mara ubora wa chakula, hivyo kuweka watumiaji katika hatari ya kupata ugonjwa wa kisukari. Hali pia imeathiriwa na ukweli kwamba taasisi za kusimamia ubora wa chakula zimejikita kusimamia viatarishi vya magonjwa yanayoambukiza tu na kuweka kando vihatarishi vya magonjwa yasiyo ya kuambukiza. Japokuwa kuna taarifa za kutosha kuhusu kiwango cha tatizo la kisukari na vihatarishi vyake, taarifa za kitafiti kuhusu njia za kifanisi za kuzuia tatizo la kisukari na vihatarishi vyake; tatizo linalozidisha mzigo wa gharama kwa mifumo ya afya ya nchi na ngazi ya familia ni haba nchini. Lengo la utafiti huu ni kutekeleza na kutathmini njia ya kupunguza vimeng’enywa hatarishi vya aina ya pili ya ugonjwa wa kisukari, na kuandaa mwongozo wa kubaini hatari za vimeng’enywa miongoni mwa walaji wa vyakula vilivyopikwa vya mitaani nchini Tanzania. Jumla ya watumiaji wa chakula cha barabara 556, angalau wadau wa chakula 50 na wadau 10 watashiriki katika utafiti huu. Utafiti huu unafadhiliwa na **Afrique One ASPIRE Chini ya Mpango wa One Health.**

Tunaomba ruhusa kukuuliza maswali ya kiujumla, uelewa na mtazamo wako kuhusu ugonjwa wa kisukari, tabia zako za maisha ya kila siku (ulaji wako wa chakula, ushughulishaji wa mwili, uvutaji wa sigara, utumiaji wa kilevi n.k) historia yako juu ya magonjwa na vipimo vya mwili (urefu, uzito, ukubwa wa paja lako na ukubwa wa kiuno) Pia tutachukua damu kidogo kutoka kwenye mkono na kidole ili kuweza kupima kiwango cha sukari na mafuta ndani ya damu. Hili litafanyika kesho asubuhi kwa sababu tunataka kuchukua damu kabla hujala kitu, Utapaswa kushiriki katika elimu ya kuhusu (juu ya vihatarishi vya kisukari, dalili, kujikinga, madhara na kisukari na lishe) itakayotolewa kwa njia ya vipeperushi na mafundisho ya vikundi na ikibidi hata kwa njia ya simu. Tunakuomba uendelee kupata huduma ya chakula kwa mtoa huduma wako kwa kipindi kisichopungua miezi mitatu. Baada ya hapo utafanyiwa mahojiano na kufanyiwa vipimo kama hapo juu kuona kama elimu na sahani ya afya vina mchango katika kupunguza vihatarishi vya kitabia na kimetabolic vya kisukari. Napenda kukuthibitishia kuwa hautachukuliwa vipimo vya ziada ya nilivyokueleza.

Matokeo ya utafiti huu yatatumika kutafuta njia za kuboresha ubora wa chakula kinachopikwa na kuuzwa mitaani na hivyo kuchangia katika kupunguza viatarishi vya ugonjwa wa kisukari kwa watumiaji.

Taarifa utakzotoa ni siri kabisa na hazitatambulika kwa mtu yeyote. Zitatumika tu kwa madhumuni ya utafiti. Jina lako, anwani, na maelezo mengine ya kibinafsi yataondolewa kwenye kanzidata, ni namba ya utambulisho tu itatumika kuunganisha jina lako na majibu yako bila kukufahamu. Zaidi; timu ya watafiti yaweza kuwaliana nawe tena baadaye iwapo itatokea kuna taarifa zako zinaitajika.

Ushiriki wako ni wa hiari na unaweza kujiondoa kwenye utafiti baada ya kukubali kushiriki. Pia huko huru kukataa kujibu swali lolote ambalo linaulizwa katika dodoso hili. Ikiwa una maswali yoyote kuhusu utafiti huu unaweza kuniuliza mimi au kuwasiliana na [jina la taasisi na maelezo ya mawasiliano] au [Mtafiti Mkuu kwenye tovuti].

Uchunguzi huu umepitishwa na kuombewa ruhusa kwa ngazi zote zinazohusika ikiwemo Taasisi ya Taifa ya Utafiti wa Magonjwa ya Binadamu (NIMR), Wizara ya Afya, Mikoa, Wilaya, kata na ngazi ya uongozi wa soko.

Iwapo wakati wowote una maswali ya kuuliza au tatizo lolote linalohusiana na utafiti huu unaweza kuwasiliana na mtafiti mkuu (principle investigator) Mr. Gibson Benard Kagaruki (0757 404142/0784593870). Kwa mawasiliano na Kamati ya Maadili ya Utafiti, Taasisi ya Taifa ya Utafiti wa Magonjwa ya Binadamu (NIMR) wasiliana nao kwa anuani zifuatayo: P.O.Box 9653, DSM, namba ya simu: 022 2121400.

Ukiweka sahahi kwenye fomu hii ya ridhaa ni kuthibitisha kwamba unaelewa nini kinatatarajiwa kwako kwa upande wa taarifa na sampuli za vipimo na ni kuthihirisha kuwa una nia ya kushiriki katika utafiti huu.

Imesomwa na mhojaji, je mhojiwa amekubali kushiriki?: 1. Amekubali 2. Amekataa

Ridhaa ya kushiriki: Mimi hapa NIMETOA RIDHAA ya kushiriki utafiti huu.

**Jina: Sahihi/Dole Gumba**

Jila la mhojaji_________________________ ___________________

Jina la mshiriki__________________________ ___________________

Shahidi (kama mshiriki hajui kusoma)_________________ ___________________

Tarehe_______________________________

# Appendix D

**CRERC FORM 13**

**TUMAINI UNIVERSITY**

**KILIMANJARO CHRISTIAN MEDICAL COLLEGE**

**P. O. Box 2240, Moshi Tel. 027-27-53909**

**CURRICULUM VITAE OF INVESTIGATORS**

1. **1.1 Name: Gibson B. Kagaruki**

1.2. Nationality: Tanzanian

1. Contact :
   1. Address: KCMUCo, P.O.Box 2240, MOSHI, Tanzania
   2. Phone: +255757404142
   3. Email: gkagaruki@gmail.com
2. Academic Qualifications (University degrees)
   1. BSc. In Applied Statistic-Mzumbe University
   2. MSc. In Health Informatics-Dar es Salaam University
   3. PhD candidate-KCMUCo
3. Employment
   1. Employer (Institution): National Institute For Medical Research Tanzania
   2. Current position: Senior Research Scientist
   3. Profession: Statistician
4. Not more than 10 publications, within past 5 years.
   1. **Gibson B Kagaruki**, Mary T Mayige, Ester S Ngadaya, Godfather D Kimaro, Akili K Kalinga, Amos M Kahwa, Godlisten S Materu and Sayoki G Mfinanga, Magnitude and risk factors of non-communicable diseases among people living with HIV in Tanzania: a cross sectional study from Mbeya and Dar es Salaam regions; BMC Public Health 2014, 14:904
   2. **Gibson B Kagaruki,** Godfather D. Kimaro, Clement N. Mweya, Andrew M. Kilale, Ray M. Mrisho, Amani F. Shao, Akili K. Kalinga, Amos M. Kahwa, Esther S. Ngadaya, Godlisten S. Materu, Sayoki G. Mfinanga, Mary T. Mayige, Prevalence and risk factors of metabolic syndrome among individuals living with HIV and receiving antiretroviral treatment in Tanzania. British Journal of Medicine & Medical Research 5(10): 1317-1327, 2015, Article no.BJMMR.2015.150 ISSN: 2231-0614
   3. Andrew Martin Kilale, Esther Ngadaya, **Gibson Benard Kagaruki**, Yakobo Leonard Lema, Julius Muhumuza, Bernard James Ngowi, Sayoki Godfrey Mfinanga, Sven Gudmund Hinderaker; Experienced and Perceived Risks of Mycobacterial Diseases: A Cross Sectional Study among Agropastoral Communities in Northern Tanzania (2015), PLoS ONE 10(6): e0130180. doi:10.1371/journal.pone.0130180.
   4. Andrew Martin Kilale, Esther Ngadaya, **Gibson Benard Kagaruki**, Yakobo Leonard Lema, Julius Muhumuza, Bernard James Ngowi, Sayoki Godfrey Mfinanga and Sven Gudmund Hinderaker; Knowledge and Perceptions about Tuberculosis in Agropastoral Communities in Northern Tanzania(2015): A Cross-Sectional Study; 10(3): 1-9, 2015, Article no.BJMMR.18973
   5. Andrew Martin Kilale,Esther Ngadaya, Julius Muhumuza, **Gibson Benard Kagaruki**, Yakobo Leonard Lema, Bernard James Ngowi, Sayoki Godfrey Mfinanga, Sven Gudmund Hinderake; Who Has Mycobacterial Disease? Across Sectional Study in Agropastoral Communities in Tanzania (2016); PLoSONE 11(5):e0153711.doi:10.1371/journal.pone.0153
   6. Jennifer Manne-Goehler, Rifat Atun, Andrew Stokes, Alexander Goehler, Dismand Houinato, Corine Houehanou, Mohamed Msaidie Salimani Hambou, Benjamin Longo Mbenza, Eugène Sobngwi, Naby Balde, Joseph Kibachio Mwangi, Gladwell Gathecha, Paul Waweru Ngugi, C Stanford Wesseh, Albertino Damasceno, Nuno Lunet, Pascal Bovet, Demetre Labadarios, Khangelani Zuma, Mary Mayige, **Gibson Kagaruki**, Kaushik Ramaiya, Kokou Agoudavi, David Guwatudde, Silver K Bahendeka, Gerald Mutungi, Pascal Geldsetzer, Naomi S Levitt, Joshua A Salomon, John S Yudkin, Sebastian Vollmer, Till Bärnighausen; Diabetes diagnosis and care in sub-Saharan Africa: pooled analysis of individual data from 12 countries, Lancet Diabetes-Endocrinol 2016 Published Online October 7, 2016 http://dx.doi.org/10.1016/ S2213-8587(16)30181-4
   7. Deus S Ishengoma, Mathias L Kamugisha, Acleus SM Rutta, **Gibson B Kagaruki**, Andrew M Kilale, Amos Kahwa, Erasmus Kamugisha, Vito Baraka, Celine I Mandara, Godlisten S Materu, Julius J Massaga, Stephen M Magesa, Martha M Lemnge, Leonard EG Mboera, [Performance of health laboratories in provision of HIV diagnostic and supportive services in selected districts of Tanzania](https://bmchealthservres.biomedcentral.com/articles/10.1186/s12913-017-2030-9) (2017), BMC Health Services Research 17(1) DOI: 10.1186/s12913-017-2030-9
   8. Ntuli A. Kapologwe, **Gibson B. Kagaruki**, Albino Kalolo, Mariam Ally, Amani Shao, Manoris Meshack, Manfred Stoermer, Amena Briet7, Karin Wiedenmayer, and Axel Hoffman: Barriers and facilitators to enrollment and re-enrollment into the community health funds/Tiba Kwa Kadi (CHF/TIKA) in Tanzania: a cross-sectional inquiry on the effects of socio-demographic factors and social marketing strategies, BMC Health Services Research, (2017) 17:308
   9. Fredirick L. Mashili , **Gibson B. Kagaruki**, Joseph Mbatia, Alphoncina Nanai, Grace Saguti, Sarah Maongezi, Ayoub Magimba, Janneth Mghamba, Mathias Kamugisha, Eric Mgina, Clement N. Mweya, Ramaiya Kaushik, and Mary T. Mayige; Physical Activity and Associated Socioeconomic Determinants in Rural and Urban Tanzania: Results from the 2012 WHO-STEPS Survey International Journal of Population Research Volume 2018 (2018), Article ID 4965193, 10 pages <https://doi.org/10.1155/2018/4965193>
   10. **Gibson B. Kagaruki**, Mathias L. Kamugisha, Andrew M. Kilale, Erasmus Kamugisha, Acleus S.M Rutta, Vito Baraka, Celine I. Mandara, Stephen M. Magesa, Godlisten Materu, Amos M. Kahwa, Rashid Madebe, Julius J. Massaga, Martha M. Lemnge, Leonard E.G. Mboera, Deus I. Ishengoma; Supply chain management of laboratory supportive services and its potential implications on the quality of HIV diagnostic services in Tanzania; Tanzania Journal of Health Research; 20(1) 2018.
5. **1.1 Name: Johnson M. Mahande**

1.2. Nationality: Tanzanian

1. Contact :
   1. Address: KCMUCo, P.O.Box 2240, MOSHI, Tanzania
   2. Phone: +255759929965
   3. Email: jmmahande@gmail.com>
2. Academic Qualifications (University degrees)
   1. Animal Science & production –Sokoine University of Agriculture
   2. Masters in Public Health-KCMUCo
   3. PhD - University of Bergen, Norway
3. Employment
   1. Employer (Institution): KCMUCo
   2. Current position: Senior Lecturer
   3. Profession: Statistician
4. Not more than 10 publications, within past 5 years.
   1. Chuwa FS, Mwanamsangu AH, Brown BG, Msuya SE, Senkoro EE, Mnali OP, et al. Maternal and fetal risk factors for stillbirth in Northern Tanzania: A registry-based retrospective Cohort study. PLoS ONE. 2017, 12(8): e0182250.
   2. **Mahande Michael Johnson**, Obure Joseph, Mvunta, Miriam Hamisi. Incidence, recurrence and predictors associated with recurrence of low birth weight in Northern Tanzania. *International Journal of Population Data Science* (2017) 1:001
   3. Birjna A. Hirani, Bariki L. Mchome, Nicholaus S. Mazuguni, **Michael J. Mahande**. The decision delivery interval in emergency caesarean section and its associated maternal and fetal outcomes at a referral hospital in northern Tanzania: a cross-sectional study. BMC Pregnancy and Child birth, 2017. 17(1):411. doi: 10.1186/s12884-017-1608-x.
   4. Tasilo Kamenya, Damian Jeremia Dami, James Samwel Ngocho, Rune Nathanael Philemon, **Michael Johnson Mahande**, Sia Emmanueli Msuya. The prevalence of hepatitis B virus among HIV-positive patients at Kilimanjaro Christian Medical Centre Referral Hospital, Northern Tanzania. *The Pan African Medical Journal*. 2017; 28:275. [doi:10.11604/pamj.2017.28.275.11926](https://www.dx.doi.org/10.11604/pamj.2017.28.275.11926)
   5. Hassan I. Njete, Pendo Mlay, **Michael Johnson Mahande**, Sia E. Msuya. Prevalence, predictors and challenges of gestational diabetes mellitus screening among pregnant women in northern Tanzania. Tropical Medicine and International Health (doi/10.1111/tmi.13018).
   6. Masanja Robert, Majige Selemani, **Michael Johnson Mahande.** Awareness and Use of Modern Contraceptives among University Students in Kilimanjaro Region, Tanzania. *International Journal of Recent Research in Social Sciences and Humanities*, 2017, 4 (4): 109-119.
   7. Osward Mwalukasa, Elizabeth K. Danstan, Clifford S. Tarimo, Blandina T. Mmbaga, **Michael J. Mahande**. Frequency and factors associated with low birth weight among neonates delivered at a tertiary hospital in Northern Tanzania. *Archive*. *Pediatrics*, 2018. JPED-141.DOI.10.29011/257X.100041
   8. Dorah Mrema; Rolv Terje Lie; Truls Ostbye; **Michael Johnson Mahande**; Anne Kjersti Daltveit. The association between pre pregnancy body mass index and risk of preeclampsia: a registry based study from Tanzania" [BMC Pregnancy Childbirth.](https://www.ncbi.nlm.nih.gov/pubmed/29466949) 2018; 18(1):56.
   9. Neema R. Mosha, **Michael Mahande**, Adinan Juma, Innocent Mboya, Rob Peck, Mark Urassa, Denna Michael & Jim Todd (2017) Prevalence, awareness and factors associated with hypertension in North West Tanzania, Global Health Action. 2017, 10 (1321279).
   10. Rugaimukam JJ, **Mahande MJ**, Msuya SE, Philemon RN. Risk Factors for Preterm Birth among Women Who Delivered Preterm Babies at Bugando Medical Centre, Tanzania. SOJ Gynecol Obstet Womens Health, 2017, 3(2):1-7.
5. **1.1 Name: Sayoki G. Mfinanga**

1.2. Nationality: Tanzanian

1. Contact :
   1. Address: NIMR-Muhimbili, P.O.Box 3436, Dar es Salaam, Tanzania
   2. Phone: +25522 2152232
   3. Email: [gsmfinanga@yahoo.com](mailto:gsmfinanga@yahoo.com); [gsmfinanga@nimr.or.tz](mailto:gsmfinanga@nimr.or.tz)
2. Academic Qualifications (University degrees)
   1. Medical Doctor- Muhimbili University College of Health Sciences
   2. PhD - University of Bergen,
3. Employment
   1. Employer (Institution): NIMR
   2. Current position: Centre Director
   3. Profession: Medical Doctor
4. Not more than 10 publications, within past 5 years.
   1. Zumla A, Chakaya J, Hoelscher M, Ntoumi F, Rustomjee R, Vilaplana C, Yeboah-Manu D, Rasolof V, Munderi P, Singh N, Aklillu E, Padayatchi N, Macete E, Kapata N, Mulenga M, Kibiki G, Mfinanga S, Nyirenda T, Maboko L, Garcia-Basteiro A, Rakotosamimanana N, Bates M, Mwaba P, Reither K, Gagneux S, Edwards **S, Mfinanga** E, Abdulla S, Cardona PJ, Russell JB, Gant V, Noursadeghi M, Elkington P, Bonnet M, Menendez C, Dieye TN, Diarra B, Maiga A, Aseffa A, Parida S, Wejse C, Petersen E, Kaleebu P, Oliver M, Craig G, Corrah T, Tientcheu L, Antonio M, Rao M, McHugh TD, Sheikh A, Ippolito G, Ramjee G, Kaufmann SH, Churchyard G, Steyn A, Grobusch M, Sanne I, Martinson N, Madansein R, Wilkinson RJ, Mayosi B, Schito M, Wallis RS, Maeurer M. Towards host-directed therapies for tuberculosis. Nature Reviews Drug Discovery (2015) doi:10.1038/nrd4696
   2. **Sayoki Mfinanga**, Duncan Chanda, Sokoine L Kivuyo, Lorna Guinness, Christian Bottomley, Victoria Simms, Carol Chijoka, Ayubu Masasi, Godfather Kimaro, Bernard Ngowi, Amos Kahwa, Peter Mwaba, Thomas S Harrison, Saidi Egwaga, Shabbar Jaffar, on behalf of the REMSTART trial team* Cryptococcal meningitis screening and community-based early adherence support in people with advanced HIV infection starting antiretroviral therapy in Tanzania and Zambia: an open-label, randomised controlled trial. www.THELANCET.com Published online March 10, 2015 http://dx.doi.org/10.1016/S0140-6736(15)60164-7 (The Lancet , Volume 385 , Issue 9983 , 2173 – 2182)
   3. **Sayoki G Mfinanga**, Bruce J Kirenga, Duncan M Chanda, Beatrice Mutayoba, Thuli Mthiyane, Getnet Yimer, Oliver Ezechi, Cathy Connolly, Vincent Kapotwe, Catherine Muwonge, Julius Massaga, Edford Sinkala, Wanze Kohi, Lucinda Lyantumba, Grace Nyakoojo, Henry Luwaga, Basra Doulla, Judith Mzyece, Nathan Kapata, Mahnaz Vahedi, Peter Mwaba, Saidi Egwaga, Francis Adatu, Alex Pym, Moses Joloba, Roxana Rustomjee, Alimuddin Zumla, Philip Onyebujoh. Early versus delayed initiation of highly active antiretroviral therapy for HIV-positive adults with newly diagnosed pulmonary tuberculosis (TB-HAART): a prospective, international, randomised, placebo-controlled trial. THELANCET, [www.thelancet.com/infection Vol 14 July 2014](http://www.thelancet.com/infection%20Vol%2014%20July%202014)
   4. Mboera, L.E.G., **Mfinanga, S.G**., Karimuribo, E.D., Rumisha, S.F. & Sindato, C., 2014, ‘The changing landscape of public health in sub-Saharan Africa: Control and prevention of communicable diseases needs rethinking’, Onderstepoort Journal of Veterinary Research 81(2) Art. #734, 6 pages. http:// dx.doi.org/10.4102/ojvr. v81i2.734
   5. Abubakar s. Hoza, Athumani M. Lupindu, **Sayoki G.M. Mfinanga**, Irmgard Moser and Brigitte König. The role of nontuberculous mycobacteria in the diagnosis, management and quantifying risks of tuberculosis in Tanga, Tanzania. Tanzania Journal of Health Research Doi: http://dx.doi.org/10.4314/thrb.v18i2.5, Volume 18, Number 2, April 2016
   6. **Sayoki G.M**. Mfinanga, Rob M. Warren, Rudovick Kazwala, Amos Kahwa, Thecla Kazimoto, Godfather Kimaro, Said Mfaume, Timothy Chonde, Esther Ngadaya, Said Egwaga, Elizabeth M. Streicher, Gey N.C. Van Pittius, Odd Morkve & Sarah Cleaveland. Genetic profile of Mycobacterium tuberculosis and treatment outcomes in human pulmonary tuberculosis in Tanzania. Tanzania Journal of Health Research, Doi: http://dx.doi.org/10.4314/thrb.v16i2.1, Volume 16, Number 2, April 2014
   7. Molloy S. F., Tom C., Greene G. S., Burry J., Govender N. P., Kanyama C., **Mfinanga S**., Lesikari S., Mapoure Y. N., Kouanfack C., Sini V., Temfack E., Boulware D. R., Dromer F., Denning D. W., Day J., Stone N. R. H., Bicanic T., Jarvis J. N., Lortholary O., Harrison T. S., Jaffar S., Loyse A. Cryptococcal meningitis: A neglected NTD? Cryptococcal meningitis: A neglected NTD? (June 29, 2017) PLoSNegl Trop Dis 11(6): e0005575. <https://doi.org/10.1371/journal.pntd.0005575>
   8. Lawson DW, James S, Ngadaya E, Ngowi B, **Mfinanga SG**, Borgerhoff Mulder M. [Reply to Rieger and Wagner: Context matters when studying purportedly harmful cultural practices.](http://www.ncbi.nlm.nih.gov/pubmed/26936949) [www.pnas.org/cgi/doi/10.1073/pnas.1601420113/](http://www.pnas.org/cgi/doi/10.1073/pnas.1601420113/) Proc Natl Acad Sci U S A. 2016 Mar 2. pii: 201601420. [Epub ahead of print] PMID: 26936949
   9. Titus Mlengeya Kamani, Rudovick Kazwala, **Sayoki Mfinanga**, Dan Haydon, Julius Keyyu, Felix Lankester and Joram Buza. One Health: a concept led by Africa, with global benefits. Veterinary Record 2015 176: 496-497 doi: 10.1136/vr.h2461
   10. [**Mfinanga SG**](http://www.ncbi.nlm.nih.gov/pubmed/?term=Mfinanga%20SG%5BAuthor%5D&cauthor=true&cauthor_uid=25541163)**,** [Kirenga BJ](http://www.ncbi.nlm.nih.gov/pubmed/?term=Kirenga%20BJ%5BAuthor%5D&cauthor=true&cauthor_uid=25541163), [Chanda DM](http://www.ncbi.nlm.nih.gov/pubmed/?term=Chanda%20DM%5BAuthor%5D&cauthor=true&cauthor_uid=25541163), [Connolly C](http://www.ncbi.nlm.nih.gov/pubmed/?term=Connolly%20C%5BAuthor%5D&cauthor=true&cauthor_uid=25541163), [Mwaba P](http://www.ncbi.nlm.nih.gov/pubmed/?term=Mwaba%20P%5BAuthor%5D&cauthor=true&cauthor_uid=25541163), [Joloba M](http://www.ncbi.nlm.nih.gov/pubmed/?term=Joloba%20M%5BAuthor%5D&cauthor=true&cauthor_uid=25541163), [Zumla A](http://www.ncbi.nlm.nih.gov/pubmed/?term=Zumla%20A%5BAuthor%5D&cauthor=true&cauthor_uid=25541163); [TB-HAART Study Group](http://www.ncbi.nlm.nih.gov/pubmed/?term=TB-HAART%20Study%20Group%5BCorporate%20Author%5D). TB-HAART trial - Authors' reply. [LANCET Infect Dis.](http://www.ncbi.nlm.nih.gov/pubmed?cmd=historysearch&querykey=1) 2015 Jan;15(1):15-6. doi: 10.1016/S1473-3099(14)71051-5.
5. **1.1** **Name: Esther S. Ngadaya**

1.2. Nationality: Tanzanian

1. Contact :
   1. Address: NIMR-Muhimbili, P.O.Box 3436, Dar es Salaam, Tanzania
   2. Phone: +25522 2152232
   3. Email: [engadaya@yahoo.com](mailto:engadaya@yahoo.com)
2. Academic Qualifications (University degrees)
   1. Medical Doctor- Muhimbili University College of Health Sciences
   2. PhD: University of Bergen
3. Employment
   1. Employer (Institution): NIMR-Muhimbili
   2. Current position: Principal Research Scientist
   3. Profession: Medical Doctor
4. Not more than 10 publications, within past 5 years.
   1. N. P. Mnyambwa & D.-J. Kim1 & E. **S. Ngadaya** & R. Kazwala & P. Petrucka & S. G. Mfinanga. Clinical implication of novel drug resistance-conferring mutations in resistant tuberculosis. Eur J Clin Microbiol Infect Dis. 07 June 2017. DOI 10.1007/s10096-017-3027-3-
   2. Lawson DW, James S, **Ngadaya E**, Ngowi B, Mfinanga SG, Borgerhoff Mulder M. [Reply to Rieger and Wagner: Context matters when studying purportedly harmful cultural practices.](http://www.ncbi.nlm.nih.gov/pubmed/26936949) [www.pnas.org/cgi/doi/10.1073/pnas.1601420113/](http://www.pnas.org/cgi/doi/10.1073/pnas.1601420113/) Proc Natl Acad Sci U S A. 2016 Mar 2. pii: 201601420. [Epub ahead of print] PMID: 26936949
   3. Kilale AM, **Ngadaya E**, Muhumuza J, Kagaruki GB, Lema YL, Ngowi BJ, et al. (2016) Who Has Mycobacterial Disease? A Cross Sectional Study in Agropastoral Communities in Tanzania. PLoS ONE 11(5): e0153711. doi:10.1371/journal.pone.0153711
   4. Lawson DW, James S, **Ngadaya E**, Ngowi B, Mfinanga SG, Borgerhoff Mulder M. [Reply to Rieger and Wagner: Context matters when studying purportedly harmful cultural practices.](http://www.ncbi.nlm.nih.gov/pubmed/26936949) [www.pnas.org/cgi/doi/10.1073/pnas.1601420113/](http://www.pnas.org/cgi/doi/10.1073/pnas.1601420113/) Proc Natl Acad Sci U S A. 2016 Mar 2. pii: 201601420. [Epub ahead of print] PMID: 26936949
   5. Keogh SC, Kimaro G, Muganyizi P, Philbin J, Kahwa A, **Ngadaya E**, et al. (2015) Incidence of Induced Abortion and Post-Abortion Care in Tanzania. PLoS ONE 10(9): e0133933. doi:10.1371/journal.pone.0133933
   6. Andrew Martin Kilale1, **Esther Ngadaya**, Gibson Benard Kagaruki, Yakobo Leonard Lema, Julius Muhumuza1, Bernard James Ngowi, Sayoki Godfrey Mfinanga and Sven Gudmund Hinderaker. Knowledge and Perceptions about Tuberculosis in Agropastoral Communities in Northern Tanzania: A Cross-Sectional Study. British Journal of Medicine & Medical Research 10(3): 1-9, 2015, Article no.BJMMR.18973, ISSN: 2231-0614
   7. Kilale AM, **Ngadaya E**, Kagaruki GB, Lema YL, Muhumuza J, Ngowi BJ, et al. (2015) Experienced and Perceived Risks of Mycobacterial Diseases: A Cross Sectional Study among Agropastoral Communities in Northern Tanzania. PLoS ONE 10(6): e0130180. doi:10.1371/journal.pone.0130180
   8. Gibson B. Kagaruki, Godfather D. Kimaro, Clement N. Mweya, Andrew M. Kilale, Ray M. Mrisho, Amani F. Shao, Akili K. Kalinga, Amos M. Kahwa, **Esther S. Ngadaya**, Godlisten S. Materu, Sayoki G. Mfinanga and Mary T. Mayige. Prevalence and Risk Factors of Metabolic Syndrome among Individuals Living with HIV and Receiving Antiretroviral Treatment in Tanzania. British Journal of Medicine & Medical Research 5(10): 1317-1327, 2015, Article no.BJMMR.2015.150 ISSN: 2231-0614
   9. [Alison Lee](http://www.pubfacts.com/author/Alison+Lee), [Paul R O C Adobamen](http://www.pubfacts.com/author/Paul+R+O+C+Adobamen), [Orighomisan Agboghoroma](http://www.pubfacts.com/author/Orighomisan+Agboghoroma), [Fahmi Oumer Ahmed](http://www.pubfacts.com/author/Fahmi+Oumer+Ahmed), [Adesuwa Aigbokhaode](http://www.pubfacts.com/author/Adesuwa+Aigbokhaode), [Ganiyu Adeniyi Amusa](http://www.pubfacts.com/author/Ganiyu+Adeniyi+Amusa), [Euripide Avokpaho](http://www.pubfacts.com/author/Euripide+Avokpaho), [Babatunde Awokola](http://www.pubfacts.com/author/Babatunde+Awokola), [Joy Ibeh](http://www.pubfacts.com/author/Joy+Ibeh), [Godsent Isiguzo](http://www.pubfacts.com/author/Godsent+Isiguzo), [Jacqueline Kagima](http://www.pubfacts.com/author/Jacqueline+Kagima), [Bankole Peter Kuti](http://www.pubfacts.com/author/Bankole+Peter+Kuti), [Hervé Lawin](http://www.pubfacts.com/author/Herve+Lawin), [Norman Lufesi](http://www.pubfacts.com/author/Norman+Lufesi), [Ndubuisi Mokogwu](http://www.pubfacts.com/author/Ndubuisi+Mokogwu), [**Esther Ngadaya**](http://www.pubfacts.com/author/Esther+Ngadaya), [Motto Malea Nganda](http://www.pubfacts.com/author/Motto+Malea+Nganda), [Ogonna Nwota Nwankwo](http://www.pubfacts.com/author/Ogonna+Nwota+Nwankwo),[Perpetua Obiajunwa](http://www.pubfacts.com/author/Perpetua+Obiajunwa), [Sunday Oghuvwu](http://www.pubfacts.com/author/Sunday+Oghuvwu), [Obianuju Ozoh](http://www.pubfacts.com/author/Obianuju+Ozoh). [Household air pollution: a call to action.](http://www.pubfacts.com/detail/25593091/Household-air-pollution-a-call-to-action) Lancet Respir Med 2015 Jan; 3(1):e1-2.
   10. Gibson B Kagaruki, Mary T Mayige, **Esther S Ngadaya**, Godfather D Kimaro, Akili K Kalinga, Andrew M Kilale, Amos M Kahwa, Godlisten S Materu and Sayoki G Mfinanga. Magnitude and risk factors of non-communicable diseases among people living with HIV in Tanzania: a cross sectional study from Mbeya and Dar es Salaam regions. BMC Public Health Sept. 2, 2014, 14:904 doi:10.1186/1471-2458-14-904
5. **1.1 Name: Daniel T. Haydon**

1.2. Nationality: British

1. Contact :
   1. Address: University of Glasgow, Glasgow, UK
   2. Phone: (0)141 330 5843
   3. Email: Haydon@glasgow.ac.uk
2. Academic Qualifications (University degrees)
   1. BSc (Hons) Biology and Oceanography- University of Southampton
   2. PhD- University of Texas
3. Employment
   1. Employer (Institution): University of Glasgow
   2. Current position: Professor and Senior Modeller
   3. Profession: Zoologist
4. Not more than 10 publications, within past 5 years.
   1. Checchi, F., Funk, S., Chandramohan, D., Chappuis, F., and **Haydon, D.T**. (2018). The impact of passive case detection on the transmission dynamics of gambiense Human African Trypanosomiasis. PLoS neglected tropical diseases 12 (4), e0006276
   2. Viana, M. , Faust, C. L. , **Haydon, D**. T., Webster, J. P. and Lamberton, P. H.L. (2017) The effects of sub-curative praziquantel treatment on life-history traits and trade-offs in drug-resistant Schistosoma mansoni. Evolutionary Applications, (doi:10.1111/eva.12558) (Early Online Publication).
   3. Trewby, H., Wright, D. M., Skuce, R. A., McCormick, C., Mallon, T. R., Presho, E. L., Kao, R. R., **Haydon, D. T**. and Biek, R. (2017) Relative abundance of Mycobacterium bovis molecular types in cattle: a simulation study of potential epidemiological drivers. BMC Veterinary Research, 13, 268.
   4. Halliday, J. E.B. , Hampson, K. , Hanley, N., Lembo, T., Sharp, J. P., **Haydon, D. T**. and Cleaveland, S. (2017) Driving improvements in emerging disease surveillance through locally-relevant capacity strengthening. Science, 357(6347), pp. 146-148.
   5. Cleaveland, S., Sharp, J., Abela-Ridder, B., Allan, K.J., Buza, J., Crump, J.A., Davis, A., Del Rio Vilas, V.J., de Glanville, W.A., Kazwala, R.R., Kibona, T., Lankester, F.J., Lugelo, A.L., Mmbaga, B.T., Rubach, M.P., Swai, E.S., Waldman, **L., Haydon**, D.T., Hampson, K., and Halliday, J.E.B. (2017) One Health contributions towards more effective and equitable approaches to health in low- and middle-income countries. Philosophical Transactions of the Royal Society B: Biological Sciences, 372(1725), 20160168.
   6. Ladbury, G., Allan, K.J., Cleaveland, S., Davis, A., de Glanville, W.A., Forde, T. L., Halliday, J.E.B., **Haydon, D.T**., Kibiki, G., Kiwelu, I., Lembo, T., Maro, V., Mmbaga, B.T., Ndyetabura, T., Sharp, J., Thomas, K., Zadok, R.N. (2017). One Health Research in Northern Tanzania–Challenges and Progress. Health Research J. (in press).
   7. King, D.J., Freimanis, G.L., Orton, R.J., Waters, R.A., **Haydon, D.T**., King, D.P. (2016). Investigating intra-host and intra-herd sequence diversity of foot-and-mouth disease virus Infection, Genetics and Evolution. 44, 286-292.
   8. Mather, A.E., Reeve, R., Mellor, D.J., Matthews, L., Reid-Smith, R.J. Dutil, L., **Haydon, D.T**., Reid, S.W.J. (2016). Detection of rare antimicrobial resistance profiles by active and passive surveillance approaches. PLoS One 11 (7), e0158515
   9. Mtema, Z., Changalucha, J., Cleaveland, S., Elias, M., Ferguson, H.M., Halliday, J.E.B., **Haydon, D.T**., Jaswant, G., Kazwala, R., Killeen, G.F., Lembo, T., Lushasi, K., Mancy, R., Maziku, M., Mbunda, E.M., Mchau, G.J.M., Murray-Smith, R., Rysava, K., Said, K., Sambo, M., Shayo, E., Sikana, L., Townsend, S.E., Urassa, H., and Hampson, K. (2016). Mobile Phones As Surveillance Tools: Implementing and Evaluating a Large-Scale Intersectoral Surveillance System for Rabies in Tanzania. PLoS Medicine. e1002002.
   10. Harvey, W.T., Benton, D.J., Gregory, V., Hall, J.P.J., Daniels, R.S., Bedford**, T., Haydon**, D.T., Hay, A.J., McCauley, J.W., Reeve, R. (2016). Identification of low-and high-impact hemagglutinin amino acid substitutions that drive antigenic drift of Influenza A (H1N1) viruses. PLoS Pathogens. e1005526.
5. **1.1 Name: Bassirou Bonfoh**

1.2. Nationality: Togolese

1. Contact :
   1. Address: CSRS Adiopodoumé, 01 BP 1303 Abidjan 01
   2. Phone: +225 23 47 27 90/ +225 23 47 27 92
   3. Email: bassirou.bonfoh@csrs.ci or [bassirou.bonfoh@gmail.com](mailto:bassirou.bonfoh@gmail.com)
2. Academic Qualifications (University degrees)
   1. Medical Doctor- Muhimbili University College of Health Sciences
   2. PhD: University of Bergen
3. Employment
   1. Employer: Centre Suisse de Recherches Scientifiques en Côte d’Ivoire (CSRS)
   2. Current position: Director General
   3. Profession: Veterinary Medicene
4. Not more than 10 publications, within past 5 years.
   1. Dindé O.A., Mobio A.J., Konan A.G., Fokou G., Yao K., Esso E.L.J.C., Fantodji A., Koussemon M., **Bonfoh B.** (**2017**). Response to the Ebola-related bushmeat consumption ban in rural Côte d’Ivoire. Agriculture & Food Security 6:28, DOI: 10.1186/s40066-017-0105-9.
   2. Schelling E, Greter H, Kessely H, Abakar MF, Ngandolo BNR, Crump L, Bold B, Kasymbekov J, Baljinnyam Z, Fokou G, Zinsstag J, **Bonfoh B**, Hattendorf J and Béchir M (**2016**) Human and animal health surveys among pastoralists. Revue scientifique et technique de l'OIE 35 (2): 659-671. doi: 10.20506/rst/35.2.2547.
   3. Zinsstag J, Abakar MF, Ibrahim M, Tschopp R, Crump L, **Bonfoh B** and Schelling E (**2016**) Cost-effective control strategies for animal and zoonotic diseases in pastoralist populations. Revue scientifique et technique de l'OIE 35 (2): 673-681. doi: 10.20506/rst.35.2.2548.
   4. Kanouté YB, Gragnon GB, Schindler C, **Bonfoh B** and Schelling E (**2016**) Epidemiology of Brucellosis, Q Fever and Rift Valley Fever at the Human and Livestock Interface in Northern Côte d'Ivoire. Acta Tropica In press.
   5. Dassi C, Mosi L, Akpatou KB, Narh CA, Quaye C, Konan DO, Djaman JA and **Bonfoh B** (**2015**) Detection of Mycobacterium ulcerans in Mastomys natalensis and Potential Transmission in Buruli ulcer endemic Areas in Côte d'Ivoire. Mycobacterial Diseases 5: 1-7.
   6. Kanouté YB, Gragnon GB, Schindler C, **Bonfoh B** and Schelling E (**2015**) Neglected zoonoses at the human and livestock interface in Northern Cote d'Ivoire. Tropical Medicine and International Health 20: 108.
   7. Koffi M, Courtin F, Kouakou L, Sepe B, Jamonneau V, **Bonfoh B** and Schelling E (**2015**) Migration between Cote d'Ivoire and bordering countries and human African trypanosomiasis. Tropical Medicine and International Health 20: 50-51.
   8. Mahamat MB, Crump L, Tidjani A, Jaeger F, Ibrahim A and **Bonfoh B** (**2015**) Food Security, Nutrition and the One Health Nexus. One Health: The Theory and Practice of Integrated Health Approaches 272.
   9. Sow I, Roesel K, Makita K, Grace D, Costard S and **Bonfoh B** (**2015**) Are malarial symptoms mistaken mistaken for brucellosis in Mali? Food safety and informal markets: Animal products in sub-saharan Africa, pp 171-175. International Livestock Research Institute (ILRI), New York (USA).
   10. **Bonfoh B**, Béchir M, Schelling E, Ouattara K, Cailleau A, Haydon D, Cleaveland S, Zinsstag J and Tanner M (**2015**) Individual and institutionnal capacity building in global health research in Africa. In: Zinsstag J and and al (eds) One health: The theory and parctice of integrated health appoaches, pp 357-365. CAB International, Wallingford (Royaume-Uni).
