# Supplementary material for: The effectiveness of interventions to reduce cardio-metabolic risk factors among regular street food consumers in Dar es Salaam, Tanzania: The pre-post findings from a cluster randomized trial (Registered by Pan African clinical trial registry with trial # PACTR202208642850935)
Source: PLoS One. 2023 Nov 15;18(11):e0289289. doi: 10.1371/journal.pone.0289289 (PMC10650998; doi:10.1371/journal.pone.0289289)
Supplement: S2 File — (DOCX) [file pone.0289289.s003.docx]

**APPENDIX I: Knowledge assessment**

| **Item** | **Item** |
| --- | --- |
| **Risk factors** | **Complications** |
| Physical inactivity | Amputation |
| Raised blood glucose | Stroke |
| Raised cholesterol | Blindness |
| Unhealthy eating | Impotence |
| Alcohol consumption | **Healthy diet (recommended amount)*** |
| Excessive body weight | Vegetables |
| **Symptoms** | Sugary foods |
| Frequent thirst | Meat |
| Frequent urination | Starchy foods |
| Extreme weight loss | Fatty foods |
| Blurred vision | High fibre foods |
| Fatigue | Fruit |
| Poor wound healing |  |

**Responses: 1.More 2.Same 3.Less and 4.Not sure*

**APPENDIX II: Assessment of positive** attitude **by using the Likert scale**

| **Statement** |
| --- |
| It is possible to prevent diabetes by dietary management |
| Family history of diabetes influences to follow disciplined life |
| Obesity increases the risk of diabetes |
| Regular exercise needs lots of effort |
| If there is increased chance of getting diabetes e.g., having family history then there is nothing to do to prevent or delay occurrences of the disease. |
| You have low efforts to prevent diabetes risks factors |
| If a person is rich and fat if the suddenly becomes thin and slender is a sign of running bankrupt |
| If somebody is fat has to maintain his/her body structure otherwise can be considered to be HIV positive |
| To become thin and slender is not good because people will consider one HIV positive. |
| A glass of unsweetened juice is better than chewing the actual fruits |
| It is very difficult to yourself to avoid overfeeding if delicious foods are plenty |
| If salt is low teste low in foods, it is better to add when you are eating in order to maintain the food teste |

Appendix III: Between marketplaces and within subject Interclass Correlation Coefficient: Using

Linear Mixed Model

| Outcome | Between sites ICC, 95%CI | Within subject ICC, 95%CI |
| --- | --- | --- |
| Waist circumference(cm) | 0.013(0.001-0.145) | 0.68(0.62-0.74) |
| Body mass index(kg/m2) | 0.019(0.002-0.17) | 0.88(0.00-1.00) |
| Diastolic BP (mmHg) | 0.022(0.003-0.127) | 0.77(0.73-0.82) |
| Systolic  BP(mmHg) | 0.017(0.002-0.129) | 0.81(0.76-0.84) |
| Fasting Blood glucose (mmol/L) | 0.001(0.000-0.003) | 0.60(0.53-0.67) |
| Fasting Triglycerides (mmol/L) | 0.023(0.004-0.124) | 0.39(0.30-0.49) |
| Attitude scores (%) | 0.024(0.005-0.116) | 0.21(0.13-0.34) |
| Knowledge scores (%) | 0.008(0.001-0.108) | 0.31(0.22-0.42) |

Appendix IV: Graphs for residual analysis to assess goodness of fit of each **parsimonious model** using both **GLMM and Bayesian Models**

| **GLMM Model** | **Bayesian Model** |
| --- | --- |
| Body Mass Index BMI | |
| 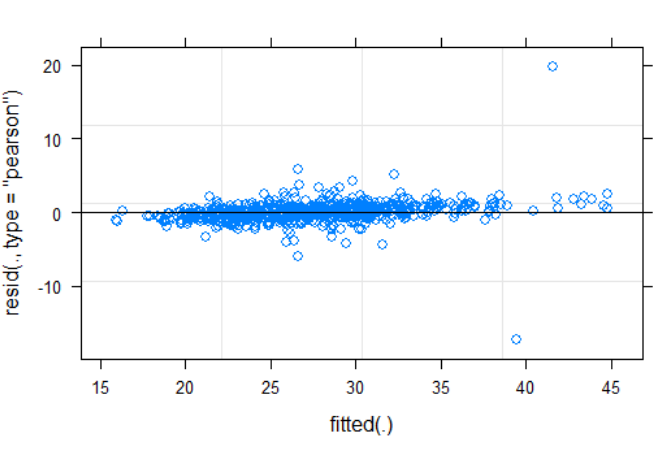 | 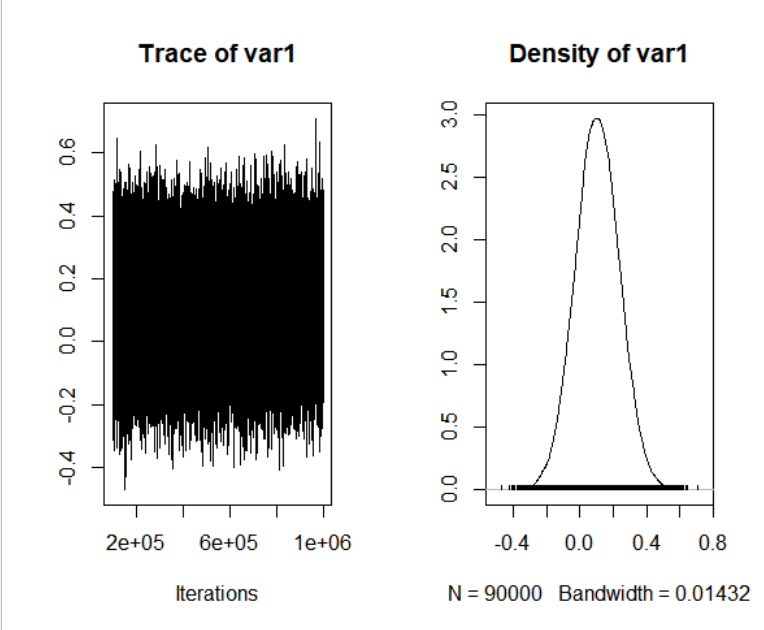 |
| Waist circumference | |
| 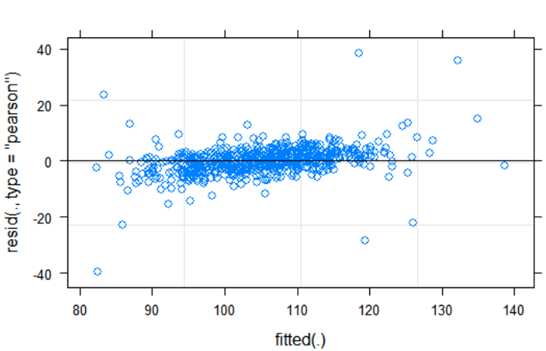 | 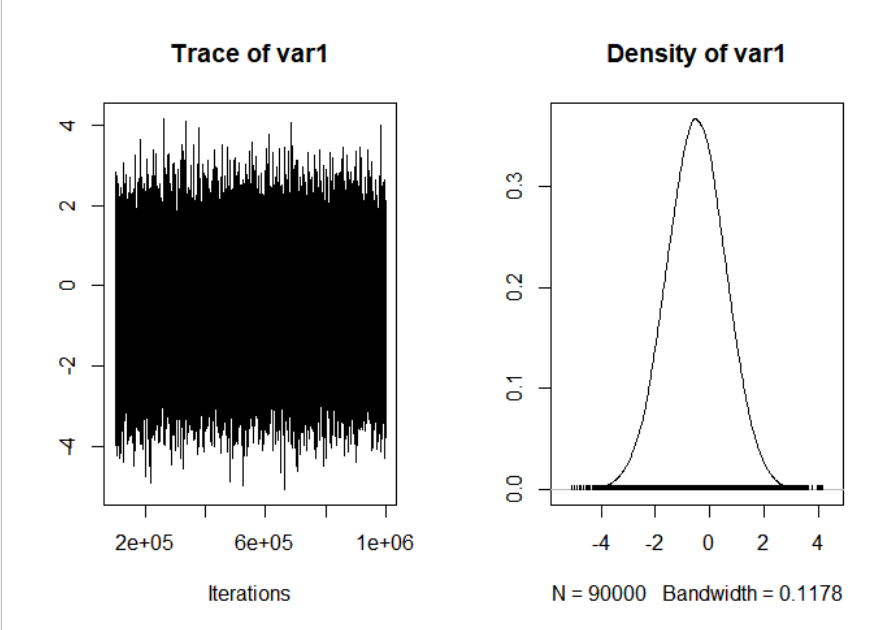 |
| Systolic BP |  |
| 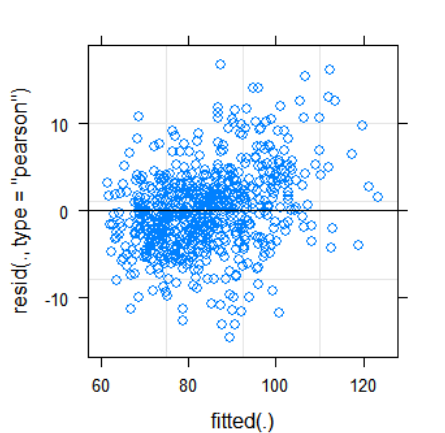 | 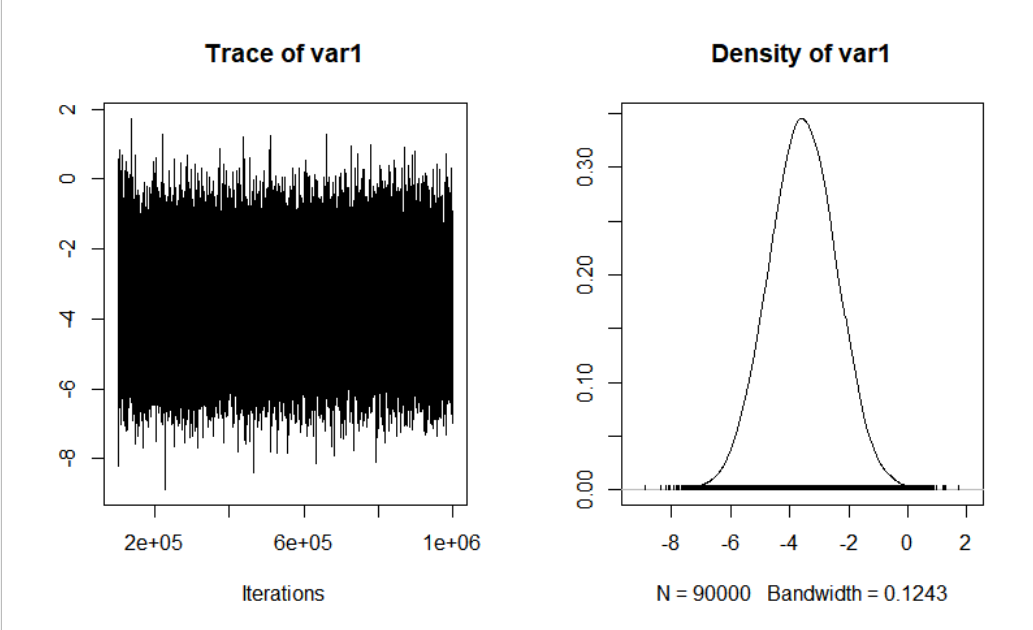 |
| Diastolic BP | |
| 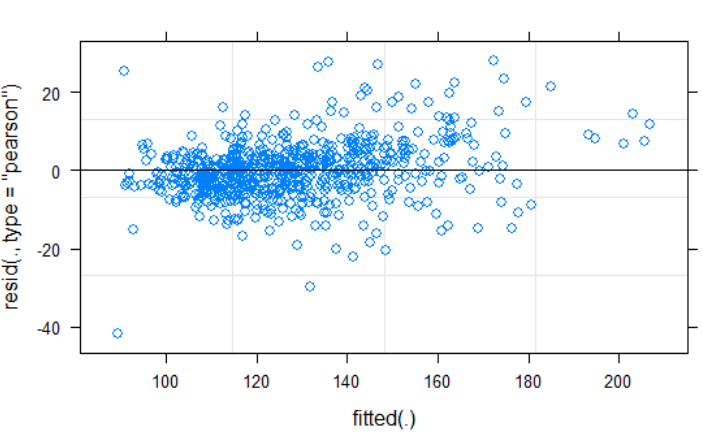 | 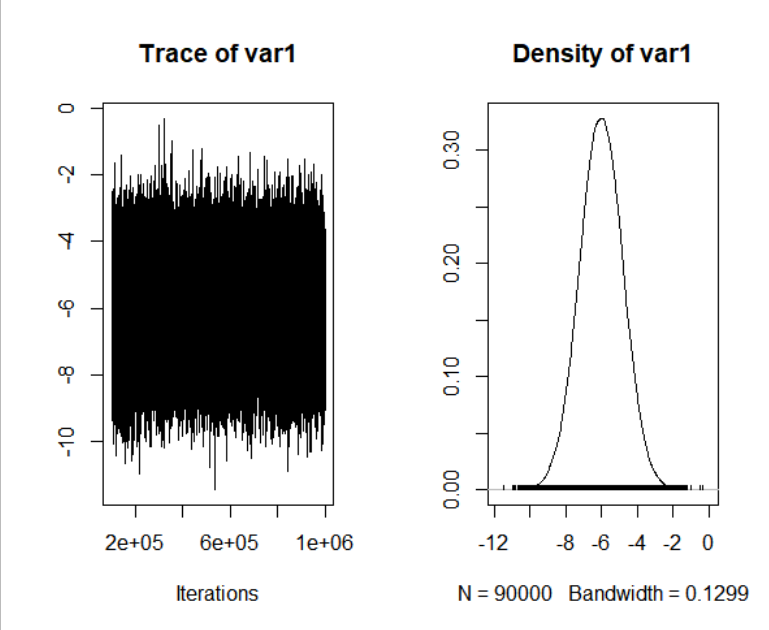 |
| **GLMM** | **Bayesian** |
| Fasting Blood Glucose (mmol/L) | |
| 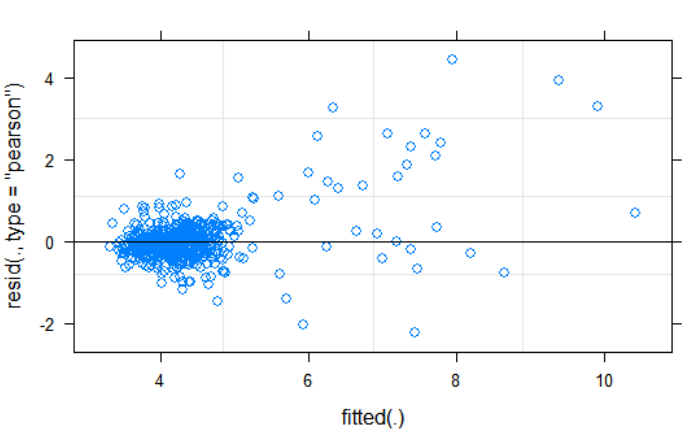 | 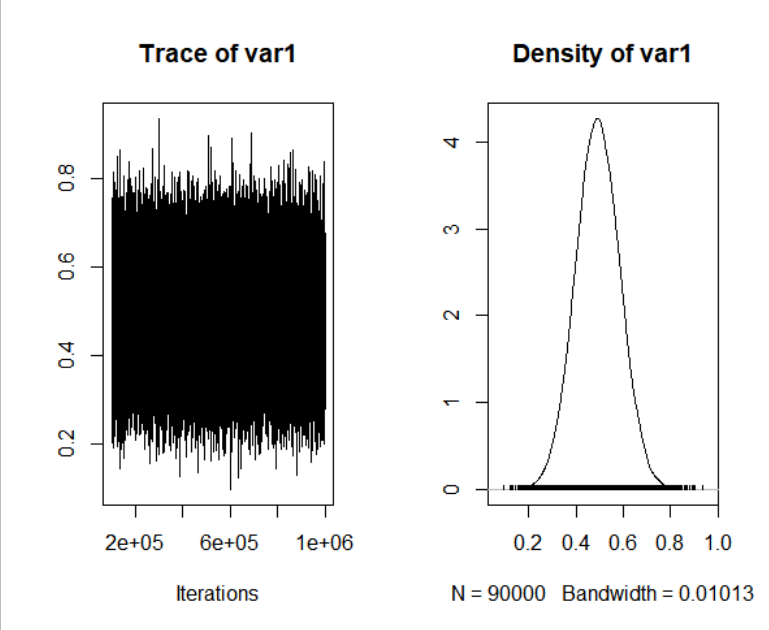 |
| Positive perception scores (%) | |
| 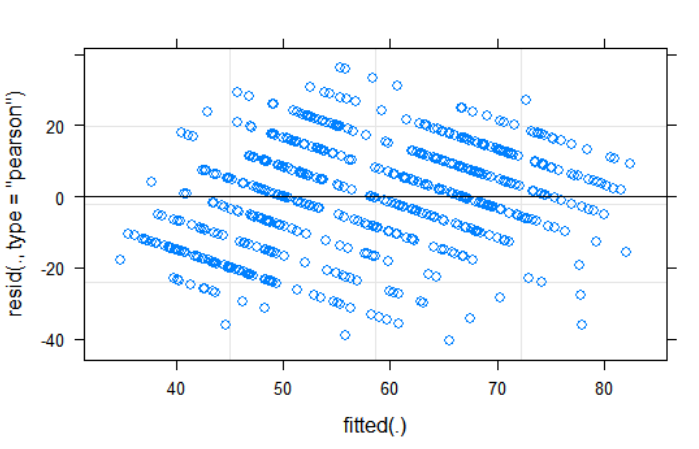 | 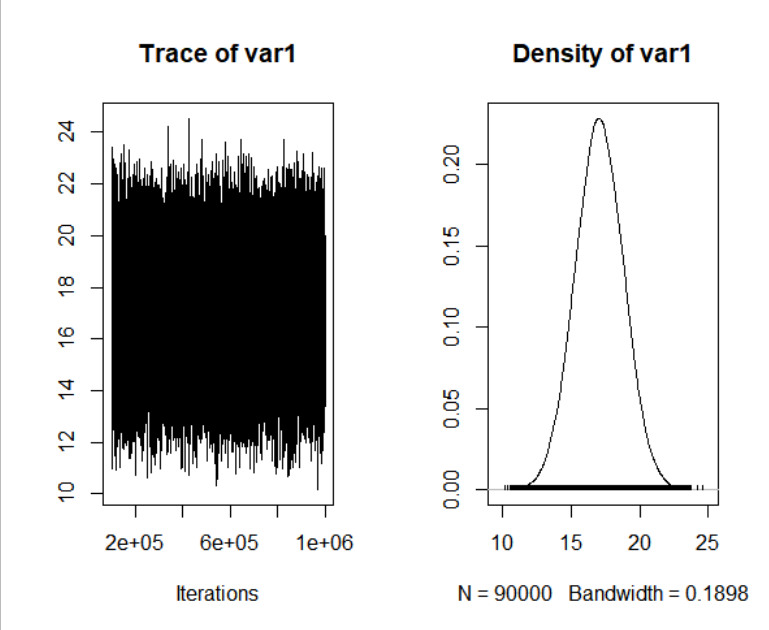 |
| Correct Knowledge % Scores | |
| 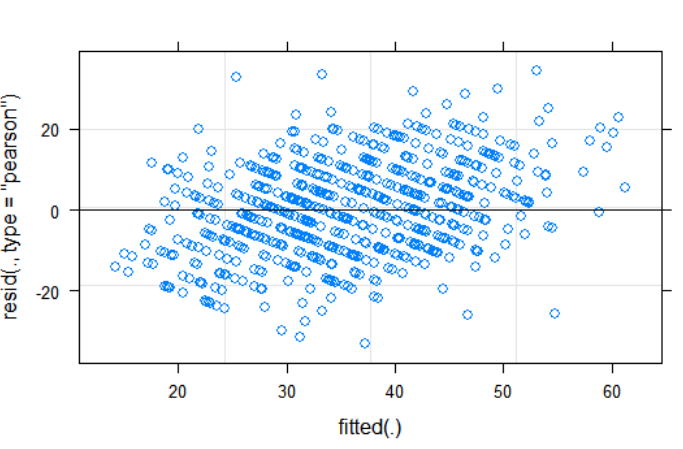 | 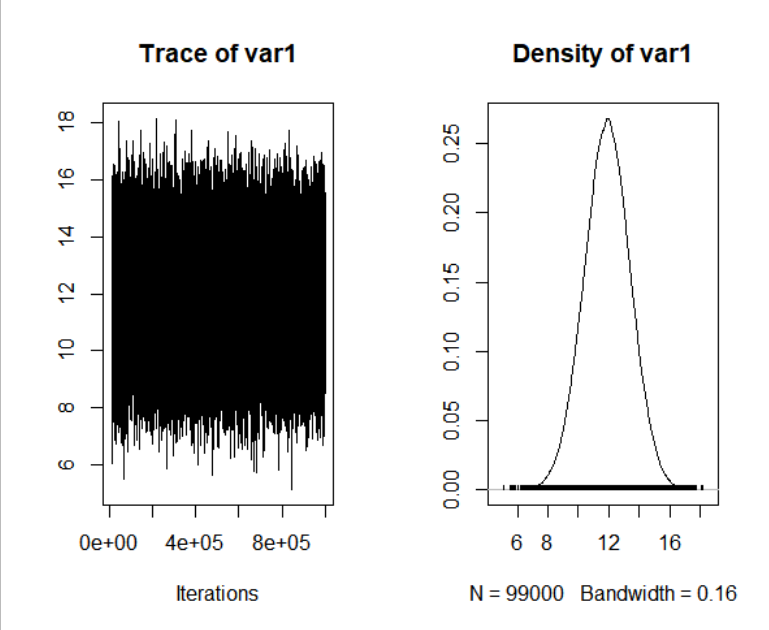 |
|  |  |

**APPENDIX V**

| Table A: Predicted estimates of the effectiveness of intervention on cardio-metabolic risk factors using Generalized Linear Mixed Models (GLMM) and Bayesian Modelling for sensitivity analysis | | | | | | | | | |
| --- | --- | --- | --- | --- | --- | --- | --- | --- | --- |
| Response Variable | Adjusted model | GLMM Model | | | Bayesian Model | | | | |
|  | Fixed factors | Estimates | SE | t-value* | Post. Mean | 1-95%CI | u-95%CI | pMCMC |  |
| Body mass index (kg/m2) | Intercept | 29.28 | 0.47 | 62.00 | 29.30 | 28.40 | 30.07 | <0.001 |  |
|  | Type of treatment |  |  |  |  |  |  |  |  |
|  | Baseline (before intervention) | Ref | Ref | Ref | Ref | Ref | Ref | Ref |  |
|  | T2DM health information only (IP1) | -0.21 | 0.24 | -0.87 | -0.21 | -0.68 | 0.27 | 0.392 |  |
|  | IP1 &Plate model (IP2) | -0.65 | 0.23 | -2.85 | -0.64 | -1.07 | -0.19 | 0.007 |  |
|  | Age | 1.22 | 0.25 | 4.79 | 1.20 | 0.71 | 1.70 | <0.001 |  |
|  | Income | 0.10 | 0.13 | 0.79 | 0.11 | -0.15 | 0.37 | 0.426 |  |
|  | Sex(male) | -2.55 | 0.50 | -5.12 | -2.51 | -2.51 | -3.46 | <0.001 |  |
|  | T2DM health information only (IP1): Age | -0.24 | 0.24 | -1.01 | -0.24 | -0.71 | 0.22 | 0.307 |  |
|  | IP1 &Plate model (IP2): Age | -0.70 | 0.24 | -2.96 | -0.71 | -1.18 | -0.25 | 0.003 |  |
|  | T2DM health information only (IP1): Income | 0.27 | 0.33 | 0.84 | 0.27 | -0.36 | 0.91 | 0.406 |  |
|  | IP1 &Plate model (IP2): Income | 0.73 | 0.33 | 2.22 | 0.77 | 0.11 | 1.41 | 0.02 |  |
| Variance for the Random effect | Individual (PID) | 22.00 | 0.26 |  |  |  |  |  |  |
|  | Site/Cluster | 0.33 | 0.03 |  |  |  |  |  |  |
| Waist Circumference (cm) | Intercept | 110.41 | 0.99 | 111.13 | 110.90 | 109.10 | 112.70 | <0.001 |  |
|  | Type of treatment |  |  |  |  |  |  |  |  |
|  | Baseline (before intervention) | Ref | Ref | Ref | Ref | Ref | Ref | Ref |  |
|  | T2DM health information only (IP1) | -1.06 | 0.74 | -1.45 | -0.49 | -2.59 | 1.68 | 0.651 |  |
|  | IP1 &Plate model (IP2) | -0.97 | 0.68 | -1.42 | -1.39 | -3.37 | 0.66 | 0.177 |  |
|  | Age | 1.61 | 0.53 | 3.06 | 1.06 | 0.18 | 1.91 | 0.018 |  |
|  | Income | 0.14 | 0.39 | 0.36 | 0.06 | -0.94 | 1.01 | 0.911 |  |
|  | Education secondary/college | 2.51 | 1.32 | 1.87 | 1.44 | -1.30 | 4.16 | 0.299 |  |
|  | Sex(male) | -7.64 | 1.34 | -6.89 | -8.25 | -10.09 | -6.42 | <0.001 |  |
|  | Marital status(Others) | -2.58 | 1.11 | -2.35 | -2.66 | -4.92 | -0.43 | 0.02 |  |
|  | T2DM health information only (IP1): income | -0.07 | 0.97 | -0.08 | 1.04 | -1.56 | 3.58 | 0.428 |  |
|  | IP1 &Plate model (IP2): income | 2.07 | 0.98 | 2.11 | 3.43 | 0.83 | 5.97 | 0.009 |  |
| Variance for the Random effect | Individual (PID) | 75.86 | 0.48 |  |  |  |  |  |  |
|  | Site/Cluster | 0.16 | 0.02 |  |  |  |  |  |  |
| Systolic Blood Pressure(mmHg) | Intercept | 124.51 | 1.96 | 63.39 | 124.80 | 121.40 | 128.30 | <0.001 |  |
|  | Type of treatment |  |  |  |  |  |  |  |  |
|  | Baseline (before intervention) | Ref | Ref | Ref | Ref | Ref | Ref | Ref |  |
|  | T2DM health information only (IP1) | -3.51 | 1.16 | -3.04 | -3.56 | -5.79 | -1.31 | 0.003 |  |
|  | IP1 &Plate model (IP2) | -0.98 | 1.07 | -0.91 | -0.96 | -3.02 | 1.13 | 0.364 |  |
|  | Age | 10.31 | 1.01 | 10.22 | 10.42 | 8.43 | 12.40 | <0.001 |  |
|  | Education (secondary/above) | -4.26 | 2.20 | -1.94 | -4.31 | -8.55 | 0.03 | 0.049 |  |
|  | Sex (male) | 7.66 | 2.01 | 3.81 | 7.08 | 3.15 | 10.98 | <0.001 |  |
|  | Marital status (Others) | 2.79 | 1.80 | 1.55 | 2.91 | 2.91 | -0.62 | 0.108 |  |
|  | T2DM health information only (IP1): Age | -1.58 | 1.11 | -1.43 | -1.59 | -3.77 | 0.59 | 0.153 |  |
|  | T2DM health information only (IP1) | 2.25 | 1.10 | 2.05 | 2.30 | 0.14 | 4.45 | 0.036 |  |
| Variance for the Random effect | Individual (PID) | 274.07 | 0.90 |  |  |  |  |  |  |
|  | Site/Cluster | 5.41 | 0.13 |  |  |  |  |  |  |
| Diastolic Blood Pressure(mmHg) | Intercept | 84.58 | 1.26 | 67.18 | 85.30 | 83.60 | 87.10 | <0.001 |  |
|  | Type of treatment |  |  |  |  |  |  |  |  |
|  | Baseline (before intervention) | Ref | Ref | Ref | Ref | Ref | Ref | Ref |  |
|  | T2DM health information only (IP1) | -5.89 | 0.74 | -8.00 | -5.99 | -8.33 | -3.623 | <0.001 |  |
|  | IP1 &Plate model (IP2) | -4.09 | 0.68 | -6.01 | -3.94 | -6.13 | -1.679 | <0.001 |  |
|  | Age | 5.61 | 0.58 | 9.75 | 5.99 | 5.05 | 6.927 | <0.001 |  |
|  | Sex(male) | 2.66 | 1.23 | 2.17 | 1.27 | -0.67 | 3.202 | 0.198 |  |
| Variance for the Random effect | Individual (PID) | 100.15 | 0.55 |  |  |  |  |  |  |
|  | Site/Cluster | 3.75 | 0.11 |  |  |  |  |  |  |
| Fasting Blood Glucose (mmol/L) | Intercept | 4.14 | 0.07 | 58.07 | 4.14 | 4.00 | 4.281 | <0.001 |  |
|  | Type of treatment |  |  |  |  |  |  |  |  |
|  | Baseline (before intervention) | Ref | Ref | Ref | Ref | Ref | Ref | Ref |  |
|  | T2DM health information only (IP1) | 0.490 | 0.090 | 5.280 | 0.493 | 0.309 | 0.676 | <0.001 |  |
|  | IP1 &Plate model (IP2) | 0.390 | 0.080 | 4.810 | 0.393 | 0.233 | 0.556 | <0.001 |  |
|  | Age | 0.210 | 0.060 | 3.380 | 0.211 | 0.086 | 0.331 | <0.001 |  |
| Variance for the Random effect | Individual (PID) | 0.840 | 0.050 |  |  |  |  |  |  |
|  | Site/Cluster | 0.010 | 0.001 |  |  |  |  |  |  |
| Fasting Triglycerides(mmol/L) | Intercept | 1.080 | 0.060 | 17.660 | 1.080 | 1.026 | 1.142 | <0.001 |  |
|  | Age | 0.100 | 0.030 | 3.150 | 0.101 | 0.042 | 0.161 | 0.001 |  |
| *If absolute of t-value is greater than two (\|2\|) then the estimate value is significant, CI: Credible Interval; “:” stand for interaction between two variables | | | | | | | | | |

| Table B: Mean predicted estimates of effectiveness of intervention arms on correct knowledge scores (%) and positive perception scores (%) using Bayesian Modelling for sensitivity analysis | | | | | | | | | | | |
| --- | --- | --- | --- | --- | --- | --- | --- | --- | --- | --- | --- |
| Response Variable | Adjusted model | GLMM Model | | | | Bayesian Model | | | | |  |
|  | Fixed factors | Estimates | SE | t-value* | Post. Mean | | 1-95%CI | u-95%CI | pMCMC |  |  |
| Positive perception scores (%) | Intercept | 48.69 | 1.81 | 26.89 | 48.2 | | 46.138 | 50.253 | <0.001 |  |  |
|  | Type of treatment |  |  |  |  | |  |  |  |  |  |
|  | Baseline (before intervention) | Ref | Ref | Ref | Ref | | Ref | Ref | Ref |  |  |
|  | T2DM health information only (IP1) | 17.73 | 1.75 | 10.1 | 17.067 | | 13.633 | 20.494 | <0.001 |  |  |
|  | IP1 &Plate model (IP2) | 18.9 | 1.64 | 11.5 | 19.433 | | 16.204 | 22.68 | <0.001 |  |  |
|  | Income | 1.41 | 0.70 | 2.01 | 1.843 | | 0.445 | 3.216 | 0.009 |  |  |
|  | Education(secondary/above) | 9.37 | 2.30 | 4.07 | 9.684 | | 5.329 | 14.055 | <0.001 |  |  |
|  | Marital status(Others) | -1.37 | -1.61 | -0.85 | -1.657 | | -5.054 | 1.716 | 0.339 |  |  |
| Variance for the Random effect | Individual (PID) | 59.54 | 0.397 |  |  | |  |  |  |  |  |
|  | Site/Cluster | 2.79 | 0.159 |  |  | |  |  |  |  |  |
| Correct knowledge scores (%) | Intercept | 31.48 | 1.50 | 21.06 | 31.199 | | 28.704 | 33.657 | <0.001 |  |  |
|  | Type of treatment |  |  |  |  | |  |  |  |  |  |
|  | Baseline (before intervention) | Ref | Ref | Ref | Ref | | Ref | Ref | Ref |  |  |
|  |  | 12.63 | 1.41 | 8.95 | 11.889 | | 8.963 | 14.889 | <0.001 |  |  |
|  | IP1 &Plate model (IP2) | 13.34 | 1.32 | 10.11 | 14.009 | | 11.201 | 16.787 | <0.001 |  |  |
|  | Age | 1.09 | 0.67 | 1.62 | 1.089 | | -0.091 | 2.284 | 0.0703 |  |  |
|  | Income | 0.46 | 0.60 | 0.77 | 0.655 | | -0.545 | 1.82 | 0.277 |  |  |
|  | Education(secondary/above) | 8.11 | 2.00 | 4.06 | 8.449 | | 4.753 | 12.271 | <0.001 |  |  |
|  | Sex(male) | -3.46 | 1.48 | -2.34 | -2.863 | | -5.463 | -0.377 | 0.0271 |  |  |
|  | Marital status (Others) | -5.23 | 1.63 | -3.21 | -5.449 | | -8.592 | -2.434 | <0.001 |  |  |
| Variance for the Random effect | Individual (PID) | 52.93 | 0.421 |  |  | |  |  |  |  |  |
|  | Site/Cluster | 8.5 | 0.091 |  |  | |  |  |  |  |  |
| **If absolute of t-value is greater than two (\|2\|) then an estimate value is significant, CI: Credible Interval* | | | | | | | | | | | |
